# Synthesis of Azuleno[2,1-*b*]quinolones and Quinolines via Brønsted Acid-Catalyzed Cyclization of 2-Arylaminoazulenes

**DOI:** 10.3390/molecules28155785

**Published:** 2023-07-31

**Authors:** Taku Shoji, Mutsumi Takeuchi, Mayumi Uda, Yukino Ariga, Akari Yamazaki, Ryuta Sekiguchi, Shunji Ito

**Affiliations:** 1 Department of Chemical Biology and Applied Chemistry, College of Engineering, Nihon University, Koriyama 963-8642, Japan; 2 Graduate School of Science and Technology, Shinshu University, Matsumoto 390-8621, Japan; 3 Department of Chemistry, School of Science, Tokyo Institute of Technology, 2-12-1 Ookayama, Meguro-ku, Tokyo 152-8551, Japan; 4 Graduate School of Science and Technology, Hirosaki University, Hirosaki 036-8561, Japan; rsekiguchi@hirosaki-u.ac.jp (R.S.); itsnj@hirosaki-u.ac.jp (S.I.)

**Keywords:** azulene, quinolone, quinoline, cyclization, aromatic nucleophilic substitution

## Abstract

Quinolone and quinoline derivatives are frequently found as substructures in pharmaceutically active compounds. In this paper, we describe a procedure for the synthesis of azuleno[2,1-*b*]quinolones and quinolines from 2-arylaminoazulene derivatives, which are readily prepared via the aromatic nucleophilic substitution reaction of a 2-chloroazulene derivative with several arylamines. The synthesis of azuleno[2,1-*b*]quinolones was established by the Brønsted acid-catalyzed intramolecular cyclization of 2-arylaminoazulene derivatives bearing two ester groups at the five-membered ring. The halogenative aromatization of azuleno[2,1-*b*]quinolones with POCl_3_ yielded azuleno[2,1-*b*]quinolines with a chlorine substituent at the pyridine moiety. The aromatic nucleophilic substitution reaction of azuleno[2,1-*b*]quinolines bearing chlorine substituent with secondary amines was also investigated to afford the aminoquinoline derivatives. These synthetic methodologies reported in this paper should be valuable in the development of new pharmaceuticals based on the azulene skeleton.

## 1. Introduction

**Figure 1:**
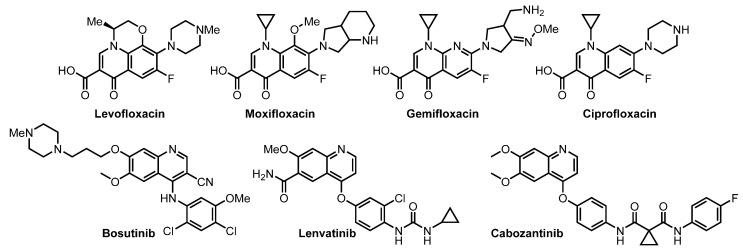
Quinolone and quinoline derivatives in clinical use.

Quinolone derivatives are compounds used as a range of antibiotics [1,2]. Thus, a number of antibiotics have been developed based on them, such as levofloxacin [3,4], moxifloxacin [5], gemifloxacin [6,7], ciprofloxacin [8,9], and so on (Figure 1). Quinoline derivatives are also compounds used as antimalarial drugs, and chloroquine [10], quinine [11], and primaquine [12] have traditionally been used. In recent years, quinoline derivatives such as bosutinib [13], lenvatinib [14], and cabozantinib [15] have been employed clinically as anticancer agents (Figure 1). Hence, synthetic methods for a variety of quinolone and quinoline derivatives have been developed to date [16].

Azulene is one of the 10π-electron non-benzenoid aromatic hydrocarbons having a fused structure of five- and seven-membered rings. To elucidate the physicochemical properties, especially optical and electrochemical properties, a variety of synthetic methods have been developed for azulene-fused derivatives [17,18,19,20,21,22,23]. However, despite the fact that azulene derivatives fused by several heterocycles, such as pyridine [24], thiophene [25], pyrrole, and furan [26,27], have been reported, the preparation of quinolone- and quinoline-fused derivatives have not been conducted so far. Since anti-inflammatory and anti-ulcer properties have been revealed in azulene derivatives [28,29], the incorporation of a quinolone and quinoline framework could create compounds with unique chemical, physical, and pharmacological characteristics that are not present in the individual compounds, potentially leading to new pharmaceutical discoveries. In this context, we sought to develop a new synthetic method for azulene-fused quinolones and quinolines, i.e., azuleno[2,1-*b*]quinolones and quinolines.

Herein, we describe a novel synthetic procedure for the synthesis of azuleno[2,1-*b*]quinolones via Brønsted acid-catalyzed intramolecular cyclization of 1,3-diethoxycarbonyl-2-arylaminoazulene derivatives. The azuleno[2,1-*b*]quinolones obtained were converted into azuleno[2,1-*b*]quinolines via halogenative aromatization with phosphoryl chloride (POCl_3_). The aromatic nucleophilic substitution reaction with several amines at the chlorine substituent of a quinoline derivative was investigated, allowing the conversion to the corresponding 4-aminoquinolines.

## 2. Results and Discussion

The acid-catalyzed intramolecular cyclization of diarylamine derivatives bearing ester function represents a valuable strategy for the construction of quinolone frameworks via Friedel–Crafts-type reaction [30]. Hence, we decided to adopt this methodology for the synthesis of azuleno[2,1-*b*]quinolones.

Initially, we investigated the preparation of 2-arylaminoazulenes, which serve as the precursors for azuleno[2,1-*b*]quinolones. In the synthesis of 2- and 6-aminoazulene derivatives, the aromatic nucleophilic substitution (S_N_Ar) reaction of haloazulenes with aliphatic amines has already been reported by our group [31,32]. In the early years of azulene chemistry, Nozoe et al. reported that the S_N_Ar reaction of diethyl 2-chloroazulene-1,3-dicarboxylate (**1**) with aniline yields the corresponding substituted product **2a** at a 59% yield [33]. However, there have been few reports on the S_N_Ar reaction of haloazulenes with aniline derivatives so far, except for an unsuccessful case [34]. In addition, since the reaction of **1** with aromatic amines with a function on the benzene ring has not been studied, we investigated the synthesis of 2-arylaminoazulene derivatives via S_N_Ar reaction with several aniline derivatives.

The yields and structures of the 2-arylaminoazulene derivatives obtained via S_N_Ar reaction are summarized in Table 1. The S_N_Ar reaction of **1** with anilines was examined by using EtOH as a solvent. The yield of the product significantly depended on the substituent on the benzene ring of arylamines, as shown in Table 1. The S_N_Ar reaction of **1** with aniline afforded 2-phenylaminoazulene derivative **2a** at a 96% yield (entry 1). The melting point of **2a** was consistent with the reported value. Moreover, even though the same reaction condition was employed, the extension of the reaction time (15 h) resulted in higher product yield compared to Nozoe’s result. Similar to aniline, arylamines bearing electron-donating group on the benzene ring also reacted with **1** to produce the desired 2-arylaminoazulene derivatives **2a**–**2e** in excellent yields (89–96%, entries 2–5). In these cases, pure products could be obtained via simple filtration protocols because the products were precipitated by cooling the reaction mixture, whereas the reaction with arylamine derivatives substituted with an electron-withdrawing group and sterically bulky 1-naphthylamine resulted in lower product yields (**2f**: 72% and **2g**: 61%; entries 6 and 8). In the S_N_Ar reaction with *p*-nitroaniline, no reaction was observed even under the reflux temperature conditions in *N*-methylpyrolidone (NMP), which was attributed to the strong electron-withdrawing nature of the nitro group (entry 7). The reaction with *N*-methylaniline afforded the desired product **2h**, but with a low yield (33%), which was probably due to the steric hindrance of the methyl group on the amine moiety (entry 9).

**Figure 2:**
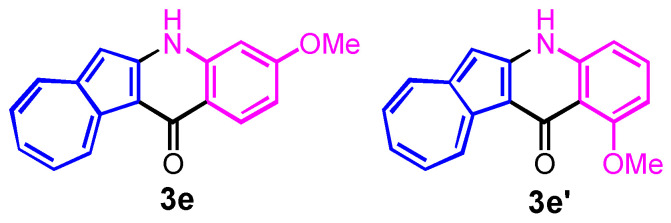
Structure of azuleno[2,1-*b*]quinolones **3e** and **3e′**.

The synthesis of azuleno[2,1-*b*]quinolone derivatives via intramolecular cyclization of 2-arylaminoazulenes **2a**–**2g** was investigated using several Brønsted acids. No reaction was observed when **2a** was treated with trifluoroacetic acid, but the reaction with 100% phosphoric acid led to complete decomposition. We found that the reaction of **2a** with polyphosphoric acid (PPA) leads to concomitant decarboxylation of the ester function to produce azulenoquinolone **3a** in excellent yield (96%) (Table 2, entry 1). Thus, the reaction condition utilizing PPA as a Brønsted acid was selected for further investigations. As shown in Table 2, all the cyclization with PPA afforded the target products **3b**–**3g** in excellent yields (85–100%), but the cyclization reaction of **2e** having a *m*-methoxy group on the arylamine moiety afforded regioisomers **3e** and **3e′** (**3e**:**3e′** = 3:2 ratio from ^1^H NMR spectrum) in 93% yield as an inseparable mixture (entry 5 and Figure 2). The resulting quinolones **3a**–**3g** were stable enough to be stored for several months under ambient conditions but had rather poor solubility in common organic solvents.

**Scheme 1:**
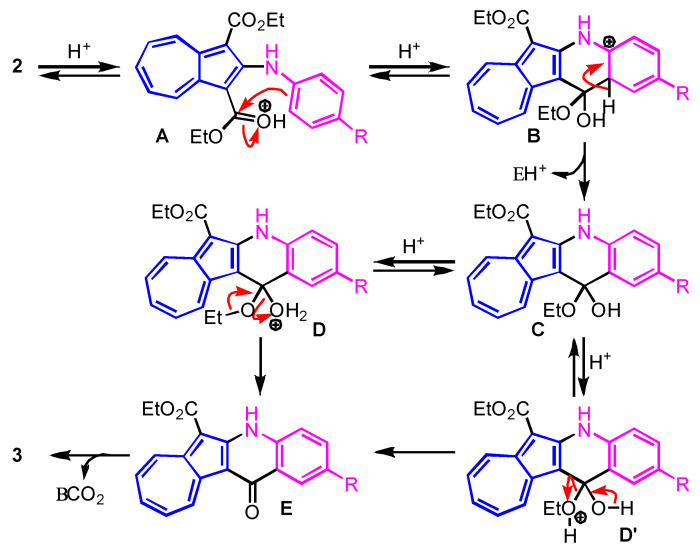
Plausible reaction mechanism for the formation of azuleno[2,1-*b*]quinolone **3**.

The plausible reaction mechanism for the formation of **3** is shown in Scheme 1. Initially, the protonation of carbonyl group of **2** by PPA forms intermediate **A**. Subsequently, the intramolecular Friedel–Craft reaction of the substituted arylamine moiety to the activated carbonyl group affords the hemiacetal intermediate **C** through the cationic intermediate B. Hemiacetal **C** is further protonated to form intermediates **D** or **D’**, which upon the elimination of water or ethanol provide the ester product **E**. Finally, the ester group on the azulene ring should be decarboxylated to afford azuleno[2,1-*b*]quinolone **3**.

Halogen substituents hold significant importance as functional groups in modern organic synthesis because they can be transformed into a diverse range of substituents through cross-coupling and nucleophilic substitution reactions [35,36,37]. Regarding the halogenation of aromatic compounds, electrophilic substitution reactions with halogens in the presence of Lewis or Brønsted acids can be utilized, or *N*-halosuccinimide (NXS) is also frequently employed for electron-rich aromatic rings. However, the introduction of halogen substituents through the electrophilic substitution reactions in electron-deficient nitrogen-containing aromatic compounds, such as pyridine and quinoline derivatives, is often difficult due to their inherently low reactivity. Therefore, we undertook an investigation into the conversion of quinolone derivatives into azulene-fused quinolines with a halogen substituent via halogenated aromatization.

**Figure 3:**
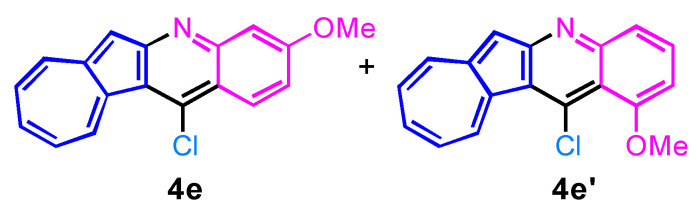
Structure of azuleno[2,1-*b*]quinolines **4e** and **4e′**.

The conversion of quinolone derivatives into azulene-fused quinolines was effectively accomplished through the reaction with phosphoryl chloride (POCl_3_). The structures and yields of the resultant products are represented in Table 3. Since the solubility of the quinolone derivatives was relatively high, POCl_3_ was employed as both solvent and reagent in this reaction. The reaction of **3a** with POCl_3_, conducted at reflux temperature, yielded the desired compound **4a** as the sole product with a 89% yield (entry 1). The quinolone derivatives **3b**–**3d** with an electron-donating group also underwent a similar reaction, leading to the formation of the corresponding quinolines **4b**–**4d** in excellent yields (**4b**: 94%, **4c**: 93%, and **4d**: 83%, entries 2–4). The mixture of **3e** and **3e′** was subjected to the reaction to yield **4e** and **4e′** at a 72% yield (entry 5, Figure 3). However, the separation of the resulting mixture proved difficult via chromatography or recrystallization. Quinolone **3f**, possessing an iodine substituent, produced **4f** along with trace amounts of **4a**; thus, the product yield of **4f** was only 61% due to the competing decomposition reaction (entry 6). Furthermore, quinolone **3g** with an extended conjugated system exhibited reactivity with POCl_3_ to afford **4g** at an 80% yield (entry 7).

In contrast to the rather low solubility of quinolones **3a**–**3g** in common organic solvents, quinolines **4a**–**4g** showed high solubility. These differences are probably due to intermolecular hydrogen bonds between the NH protons and the carbonyl groups, which causes **3a**–**3g** to exist as multimers in the solid state, resulting in low solubility, whereas the high solubility of **4a**–**4g** is attributable to the absence of such interactions.

As discussed in the Introduction section, multiply functionalized quinolines are included in a significant cohort of relevant moieties related to biologically and pharmaceutically active compounds. Considering the proficiency of the chlorine function within the aromatic ring as an efficacious leaving group in the S_N_Ar reaction, it is anticipated that the chlorine substituent of quinoline shall undergo substitution reactions with a diverse range of nucleophiles, thereby facilitating the conversion into an array of substituents. Therefore, the amination of **5a** by the S_N_Ar reaction was investigated.

Recently, Poole et al. disclosed that the S_N_Ar reaction of 4-chloroquinoline with pyrrolidine occurs at 150 °C with microwave irradiation, forming 4-pyrrolidinylquinoline in 89% yield [38]. Furthermore, Cirujano, Martí-Gastaldo, and their collaborators demonstrated that the reaction of 4-chloroquinoline with morpholine in the presence of a zinc catalyst yields 4-morpholylquinoline. However, this transformation resulted in a low product yield (25%) and required a prolonged reaction time of 24 h [39].

**Scheme 2:**
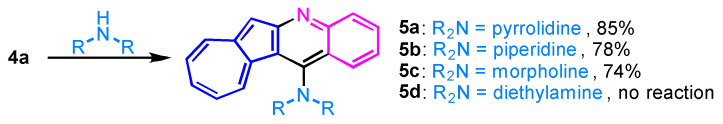
S_N_Ar reactions of **4a** with secondary amines.

It was noted that the S_N_Ar reaction of **4a** with pyrrolidine was completed at ambient temperature to give 4-pyrrolidinylquinoline **5a** in excellent yield (85%, Scheme 2). Furthermore, the utilization of piperidine and morpholine in the reaction with **4a** yielded the corresponding substituted products **5b** (78%) and **5c** (74%) with satisfactory yields, although slight heating conditions (50 °C) were required. These findings contrast with the requirements of higher temperature and prolonged reaction time for the reaction involving 4-chloroquinoline. These results might probably be attributed to the extension of the conjugated system by the fused azulene ring, which reduces the HOMO-LUMO energy gap, thereby lowering the LUMO energy level and facilitating the substitution with nucleophilic reagents. Meanwhile, the reaction with diethylamine, which is less nucleophilic than cyclic amines [40], did not proceed, and **4a** was recovered.

These new compounds **2a**–**2i**, **3a**–**3g**, **4a**–**4h**, and **5a**–**5d** were fully characterized based on their spectroscopic data, as summarized in the Experimental Section and Supplementary Materials. The ^1^1H NMR spectroscopic assignments of the reported compounds were confirmed by COSY. The HRMS spectra of the new compounds ionized by ESI showed the expected molecular ion peaks. These results are consistent with the given structures of these products (Figures S1–S50).

## 3. Materials and Methods

**General**: Melting points were determined with a Yanagimoto MPS3 micromelting apparatus. The HRMS data were obtained with a Waters Xevo G2-XS QTof spectrometer. IR spectrum was recorded with JASCO FTIR-4100 spectrophotometer. ^1^H and ^13^C NMR spectra were recorded with a JEOL ECA500 spectrometer at 500 MHz and 125 MHz, respectively.

Diethyl 2-(phenylamino)azulene-1,3-dicarboxylate (**2a**): A solution of **1** (1.59 g, 5.19 mmol) and aniline (1.46 g, 15.7 mmol) in EtOH (16 mL) was refluxed for 15 h. After the reaction mixture was cooled, the generated precipitate was collected via filtration to afford **2a** (1.79 g, 95%) as an orange solid. M.p. 138–139 °C (lit. 141–142 °C); IR (ATR): ν_max_ = 1674 (C=O) cm^−1^; ^1^H NMR (500 MHz, CDCl_3_): δ_H_ = 10.03 (br.s, 1H, NH), 9.06 (dd, 2H, *J* = 10.2, 1.6 Hz, H_4,8_), 7.57–7.51 (m, 3H, H_6_, *m*-Ph), 7.32–7.29 (m, 2H, H_5,7_), 7.23 (dd, 2H, *J* = 8.6, 1.1 Hz, *o*-Ph), 7.08 (t, 1H, *J* = 7.3 Hz, *p*-Ph), 4.09 (q, 4H, *J* = 7.2 Hz, CO_2_Et), 1.17 (t, 6H, *J* = 7.2 Hz, CO_2_Et) ppm; ^13^C NMR (125 MHz, CDCl_3_): δ_C_ = 166.5, 155.3, 144.5, 142.8, 134.1, 132.1, 131.8, 129.3, 123.9, 119.6, 103.8, 60.3, 14.3 ppm; HRMS (ESI-TOF): calcd for C_22_H_21_NO_4_ + Na^+^ [M + Na]^+^ 386.1363; found: 386.1373.

Diethyl 2-(*p*-tolylamino)azulene-1,3-dicarboxylate (**2b**): A solution of **1** (616 mg, 2.01 mmol) and *p*-toluidine (648 mg, 6.05 mmol) in EtOH (6 mL) was refluxed for 16 h. After the reaction mixture was cooled, the generated precipitate was collected via filtration to afford **2b** (679 mg, 89%) as an orange solid. M.p. 148–149 °C; IR (ATR): ν_max_ = 1690 (C=O), 1645 (s), 1587 (m), 1555 (s), 1513 (s), 1475 (m), 1432 (m), 1385 (m), 1302 (m), 1268 (w), 1230 (m), 1206 (m), 1167 (s), 1111 (m), 1031 (m), 987 (w), 943 (w), 928 (w), 903 (w), 880 (w), 858 (w), 823 (m), 802 (w), 784 (m), 761 (w), 734 (m), 701 (w), 668 (w), cm^−1^; ^1^H NMR (500 MHz, CDCl_3_): δ_H_ = 9.97 (br.s, 1H, NH), 9.02–9.00 (m, 2H, H_4,8_), 7.46–7.55 (m, 3H, H_5,6,7_), 7.13–7.10 (m, 4H, *o,m*-Ph), 4.09 (q, 4H, *J* = 7.1 Hz, CO_2_Et), 2.32 (s, 3H, Me), 1.18 (t, 6H, *J* = 7.1 Hz, CO_2_Et) ppm; ^13^C NMR (125 MHz, CDCl_3_): δ_C_ = 166.6, 155.7, 144.6, 140.2, 133.8, 133.6, 131.73, 131.71, 129.8, 119.8, 103.6, 60.3, 21.0, 14.3 ppm; HRMS (ESI-TOF): calcd for C_23_H_23_NO_4_ + Na^+^ [M + Na]^+^ 400.1519; found: 400.1527.

Diethyl 2-[(*p*-butylphenyl)amino]azulene-1,3-dicarboxylate (**2c**): A solution of **1** (617 mg, 2.01 mmol) and *p*-butylaniline (905 mg, 6.06 mmol) in EtOH (6 mL) was refluxed for 16.5 h. After the reaction mixture was cooled, the generated precipitate was collected via filtration to afford **2c** (752 mg, 89%) as an orange solid. M.p. 87–88 °C; IR (ATR): ν_max_ = 1685 (C=O) cm^−1^; ^1^H NMR (500 MHz, CDCl_3_): δ_H_ = 9.99 (br.s, 1H, NH), 9.01 (d, 2H, *J* = 10.0 Hz, H_4,8_), 7.55–7.48 (m, 3H, H_5,6,7_), 7.13 (m, 4H, *o,m*-Ph), 4.08 (q, 4H, *J* = 7.1 Hz, CO_2_Et), 2.58 (t, 2H, *J* = 7.4 Hz, *n*-Bu), 1.57 (sext., 2H, *n*-Bu), 1.36 (quint., 2H, *J* = 7.4 Hz, *n*-Bu), 1.17 (t, 6H, *J* = 7.1 Hz, CO_2_Et), 0.93 (t, 3H, *J* = 7.4 Hz, *n*-Bu) ppm; ^13^C NMR (125 MHz, CDCl_3_): δ_C_ = 166.6, 155.6, 144.6, 140.4, 138.8, 133.8, 131.73, 131.71, 129.2, 119.7, 103.6, 60.3, 35.2, 34.0, 22.3, 14.3, 14.0 ppm; HRMS (ESI-TOF): calcd for C_26_H_29_NO_4_ + Na^+^ [M + Na]^+^ 442.1989; found: 442.1995.

Diethyl 2-[(*p*-methoxyphenyl)amino]azulene-1,3-dicarboxylate (**2d**): A solution of **1** (616 mg, 2.01 mmol) and *p*-anisidine (742 mg, 6.02 mmol) in EtOH (6 mL) was refluxed for 21 h. After the reaction mixture was cooled, the generated precipitate was collected via filtration to afford **2d** (760 mg, 96%) as an orange solid. M.p. 156–157 °C; IR (ATR): ν_max_ = 1688 (C=O) cm^−1^; ^1^H NMR (500 MHz, CDCl_3_): δ_H_ = 9.94 (br.s, 1H, NH), 8.97 (d, 2H, *J* = 10.0 Hz, H_4,8_), 7.53–7.46 (m, 3H, H_5,6,7_), 7.16 (d, 2H, *J* = 8.9 Hz, *m*-Ph), 6.85 (d, 2H, *J* = 8.9 Hz, *o*-Ph), 4.10 (q, 4H, *J* = 7.0 Hz, CO_2_Et), 3.80 (s, 3H, OMe), 1.21 (t, 6H, *J* = 7.0 Hz, CO_2_Et) ppm; ^13^C NMR (125 MHz, CDCl_3_): δ_C_ = 166.6, 156.6, 156.1, 144.6, 136.1, 133.6, 131.7, 131.5, 121.5, 114.5, 103.3, 60.3, 55.7, 14.3 ppm; HRMS (ESI-TOF): calcd for C_23_H_23_NO_5_ + Na^+^ [M + Na]^+^ 416.1468; found: 416.1483.

Diethyl 2-[(*m*-methoxyphenyl)amino]azulene-1,3-dicarboxylate (**2e**): A solution of **1** (615 mg, 2.00 mmol) and *m*-anisidine (757 mg, 6.15 mmol) in EtOH (6 mL) was refluxed for 17.5 h. The reaction mixture was extracted with AcOEt/hexane, and the organic layer was washed with aq. HCl and brine, dried over Na_2_SO_4_. The solvent was concentrated under reduced pressure, and the residue was purified by recrystallization with ethanol to afford **2e** (723 mg, 92%) as an orange solid. M.p. 105–106 °C; IR (ATR): ν_max_ = 1673 (C=O) cm^−1^; ^1^H NMR (500 MHz, CDCl_3_): δ_H_ = 10.02 (s, 1H, NH), 9.05 (d, *J* = 10.3 Hz, 2H, H_4,8_), 7.57–7.51 (m, 3H, H_5,6,7_), 7.20 (t, 1H, *J* = 8.2 Hz, H_5_ of Ph), 6.80–6.83 (m, 2H, H_2,4_ of Ph), 6.64 (dd, 1H, *J* = 8.2, 2.3 Hz, H_6_ of Ph), 4.12 (q, 4H, *J* = 7.2 Hz, CO_2_Et), 3.77 (s, 3H, OMe), 1.20 (t, 6H, *J* = 7.2 Hz, CO_2_Et) ppm; ^13^C NMR (125 MHz, CDCl_3_): δ_C_ = 166.5, 160.7, 155.0, 144.4, 144.0, 134.2, 132.2, 131.7, 130.0, 112.1, 110.4, 104.6, 103.9, 60.3, 55.4, 14.3 ppm; HRMS (ESI-TOF): calcd for C_23_H_23_NO_5_ + Na^+^ [M + Na]^+^ 416.1468; found: 416.1483.

Diethyl 2-[(*p*-iodophenyl)amino]azulene-1,3-dicarboxylate (**2f**): A solution of **1** (615 mg, 2.00 mmol) and *p*-iodoaniline (1.31 g, 6.00 mmol) in EtOH (6 mL) was refluxed for 45 h. After the reaction mixture was cooled, the generated precipitate was collected via filtration to afford **2f** (707 mg, 72%) as an orange solid. M.p. 152–153 °C; IR (ATR): ν_max_ = 1677 (C=O) cm^−1^; ^1^H NMR (500 MHz, CDCl_3_): δ_H_ = 10.01 (s, 1H, NH), 9.11–9.08 (m, 2H, H_4,8_), 7.56–7.60 (m, 5H, H_5,6,7_ and *m*-Ph), 6.99 (d, 2H, *J* = 8.6 Hz, *o*-Ph), 4.14 (q, 4H, *J* = 7.1 Hz, CO_2_Et), 1.20 (t, 6H, *J* = 7.1 Hz, CO_2_Et) ppm; ^13^C NMR (125 MHz, CDCl_3_): δ_C_ = 166.4, 154.7, 144.4, 142.7, 138.1, 134.7, 132.6, 131.9, 121.4, 103.9, 86.1, 60.4, 14.3 ppm; HRMS (ESI-TOF): calcd for C_22_H_20_NO_4_I + Na^+^ [M + Na]^+^ 512.0329; found: 512.0345.

Diethyl 2-(naphthalen-1-ylamino)azulene-1,3-dicarboxylate (**2g**): A solution of **1** (616 mg, 2.01 mmol) and 1-naphthylamine (867 mg, 6.05 mmol) in EtOH (6 mL) was refluxed for 15 h. After the reaction mixture was cooled, the generated precipitate was collected via filtration to afford **2g** (507 mg, 61%) as an orange solid. M.p. 164–165 °C; IR (ATR): ν_max_ = 1693 (C=O) cm^−1^; ^1^H NMR (500 MHz, CDCl_3_): δ_H_ = 10.31 (s, 1H, NH), 9.03 (dd, 2H, *J* = 10.0, 1.4 Hz, H_4,8_), 8.34 (d, 1H, *J* = 8.2 Hz, H_8_ of Naph.), 7.89–7.87 (m, 1H, H_5_ of Naph.), 7.64 (d, *J* = 8.2 Hz, 1H, H_4_ of Naph.), 7.59–7.49 (m, 5H, H_5,6,7_ and H_6,7_ of Naph.), 7.44 (d, 1H, *J* = 7.9 Hz, H_2_ of Naph.), 7.38 (t, 1H, *J* = 7.9 Hz, H_3_ of Naph.), 3.92 (d, 4H, *J* = 6.9 Hz, CO_2_Et), 0.89 (t, 6H, *J* = 6.9 Hz, CO_2_Et) ppm; ^13^C NMR (125 MHz, CDCl_3_): δ_C_ = 166.6, 156.8, 144.5, 139.3, 134.6, 134.0, 131.9, 131.7, 128.3, 128.2, 126.6, 126.4, 125.9, 124.7, 122.4, 115.4, 103.8, 60.2, 13.9 ppm; HRMS (ESI-TOF): calcd for C_26_H_23_NO_4_ + Na^+^ [M + Na]^+^ 436.1519; found: 436.1540.

Diethyl 2-(*N*-methyl-*N*-phenylamino)azulene-1,3-dicarboxylate (**2h**): A solution of **1** (309 mg, 1.01 mmol) and *N*-methylaniline (324 mg, 3.02 mmol.) was refluxed for 1.5 h. The reaction mixture was poured into 5% HCl and extracted with AcOEt. The organic layer was washed with brine and dried over Na_2_SO_4_. The solvent was concentrated under reduced pressure, and the residue was purified by silica gel column chromatography with hexane/AcOEt (4:1) to afford **2h** (124 mg, 33%) as an orange solid. M.p. 99–100 °C; IR (ATR): ν_max_ = 1676 (C=O) cm^−1^; ^1^H NMR (500 MHz, CDCl_3_): δ_H_ = 9.34 (d, 2H, *J* = 10.0 Hz, H_4,8_), 7.73 (d, 1H, *J* = 10.0 Hz, H_6_), 7.62 (t, 2H, *J* = 10.0 Hz, H_5,7_), 7.17 (dd, 2H, *J* = 8.2, 7.3 Hz, *m*-Ph), 6.82–6.79 (m, 3H, *o,p*-Ph), 4.13 (q, 4H, *J* = 7.2 Hz, CO_2_Et), 3.45 (s, 3H, Me), 1.18 (t, 6H, *J* = 7.2 Hz, CO_2_Et) ppm; ^13^C NMR (125 MHz, CDCl_3_): δ_C_ = 165.1, 157.9, 148.8, 143.1, 137.9, 136.0, 130.6, 128.9, 119.1, 114.3, 112.9, 60.3, 41.3, 14.3 ppm; HRMS (ESI-TOF): calcd for C_23_H_23_NO_4_ + Na^+^ [M + Na]^+^ 400.1519; found: 400.1527.

Azuleno[2,1-*b*]quinolin-12(5*H*)-one (**3a**): To a solution of **2a** (614 mg, 1.69 mmol) in polyphosphoric acid (17 mL) was stirred at 140 °C for 6 h. After the reaction mixture was cooled, it was poured into water. The generated precipitate was collected via filtration to afford **3a** (396 mg, 96%) as a reddish-brown solid. M.p. 245 °C (decomp.); IR (ATR): ν_max_ = 1698 (C=O) cm^−1^; ^1^H NMR (500 MHz, DMSO-*d*_6_): δ_H_ = 12.30 (s, 1H, NH), 9.56–9.54 (m, 1H, H_11_), 8.36–8.33 (m, 2H, H_2,7_), 7.72–7.69 (m, 1H), 7.62–7.58 (m, 3H, H_11_), 7.46–7.42 (m, 1H), 7.34–7.31 (m, 1H, H_8_), 7.11 (s, 1H, H_6_) ppm; ^13^C NMR (125 MHz, DMSO-*d*_6_): δ_C_ = 175.5, 151.8, 144.9, 140.4, 140.1, 134.9, 134.5, 132.5, 131.9, 130.4, 129.0, 126.3, 124.1, 122.2, 118.0, 110.3, 104.3 ppm; HRMS (ESI-TOF): calcd for C_17_H_11_NO + H^+^ [M + H]^+^ 246.0913; found: 246.0927.

2-Methylazuleno[2,1-*b*]quinolin-12(5*H*)-one (**3b**): To a solution of **2b** (382 mg, 1.01 mmol) in polyphosphoric acid (10 mL) was stirred at 140 °C for 2.5 h. After the reaction mixture was cooled, it was poured into water. The generated precipitate was collected via filtration to afford **3b** (224 mg, 85%) as a reddish-brown solid. M.p. > 300 °C; IR (ATR): ν_max_ = 1629 (C=O) cm^−1^; ^1^H NMR (500 MHz, DMSO-*d*_6_): δ_H_ = 12.21 (s, 1H, NH), 9.53–9.51 (m, 1H, H_11_), 8.31 (d, 1H, *J* = 10.3 Hz, H_7_), 8.15 (s, 1H, H_1_), 7.51–7.57 (m, 4H, H_3,4,9,10_), 7.40–7.44 (m, 1H, H_8_), 7.09 (s, 1H, H_6_), 2.44 (s, 3H, Me) ppm; ^13^C NMR (125 MHz, DMSO-*d*_6_): δ_C_ = 175.4, 151.7, 144.8, 140.2, 138.3, 134.6, 134.3, 133.7, 131.7, 131.3, 130.4, 128.9, 125.7, 124.0, 117.9, 110.2, 104.2, 21.3 ppm; HRMS (ESI-TOF): calcd for C_18_H_13_NO + H^+^ [M + H]^+^ 260.1070; found: 260.1071.

2-Butylazuleno[2,1-*b*]quinolin-12(5*H*)-one (**3c**): To a solution of **2c** (420 mg, 1.00 mmol) in polyphosphoric acid (10 mL) was stirred at 140 °C for 2 h. After the reaction mixture was cooled, it was poured into water. The generated precipitate was collected via filtration to afford **3c** (260 mg, 86%) as a reddish-brown solid. M.p. 239–240 °C; IR (ATR): ν_max_ = 1627 (C=O) cm^−1^; ^1^H NMR (500 MHz, DMSO-*d*_6_): δ_H_ = 12.22 (s, 1H, NH), 9.53–9.51 (m, 1H, H_11_), 8.31 (d, 1H, *J* = 10.6 Hz, H_7_), 8.14 (s, 1H, H_1_), 7.57–7.52 (m, 4H, H_3,4,9,10_), 7.44–7.40 (m, 1H, H_8_), 7.09 (s, 1H, H_6_), 2.72 (t, 2H, *J* = 7.4 Hz, *n*-Bu), 1.57–1.63 (m, 2H, *n*-Bu), 1.31 (sext., 2H, *J* = 7.4 Hz, *n*-Bu), 0.89 (t, 3H, *J* = 7.4 Hz, *n*-Bu) ppm; ^13^C NMR (125 MHz, DMSO-*d*_6_): δ_C_ = 175.5, 151.7, 144.8, 140.2, 138.5, 136.3, 134.6, 134.3, 133.0, 131.6, 130.4, 128.9, 125.1, 124.0, 118.0, 110.3, 104.3, 35.0, 33.9, 22.2, 14.4 ppm; HRMS (ESI-TOF): calcd for C_21_H_19_NO + H^+^ [M + H]^+^ 302.1539; found: 302.1522.

2-Methoxyazuleno[2,1-*b*]quinolin-12(5*H*)-one (**3d**): To a solution of **2d** (394 mg, 1.00 mmol) in polyphosphoric acid (10 mL) was stirred at 140 °C for 3 h. After the reaction mixture was cooled, it was poured into water. The generated precipitate was collected via filtration to afford **3d** (250 mg, 91%) as a reddish-brown solid. M.p. 240 °C (decomp.); IR (ATR): ν_max_ = 1663 (C=O) cm^−1^; ^1^H NMR (500 MHz, DMSO-*d*_6_): δ_H_ = 12.28 (s, 1H, NH), 9.50 (d, 1H, *J* = 9.5 Hz, H_11_), 8.28 (d, 1H, *J* = 10.6 Hz, H_7_), 7.80 (d, 1H, *J* = 2.9 Hz, H_1_), 7.60–7.51 (m, 3H, H_4,9,10_), 7.41–7.35 (m, 2H, H_3_,_8_), 7.08 (s, 1H, H_6_), 3.86 (s, 3H, OMe) ppm; ^13^C NMR (125 MHz, DMSO-*d*_6_): δ_C_ = 174.9, 155.1, 151.3, 144.7, 140.3, 134.7, 134.4, 134.2, 131.4, 130.3, 128.8, 125.0, 122.1, 119.6, 110.0, 106.6, 104.4, 56.0 ppm; HRMS (ESI-TOF): calcd for C_18_H_13_NO_2_ + H^+^ [M + H]^+^ 276.1019; found: 276.1018.

Cyclization reaction of **2e**: To a solution of **2e** (396 mg, 1.01 mmol) in polyphosphoric acid (10 mL) was stirred at 140 °C for 2.5 h. After the reaction mixture was cooled, it was poured into water. The generated precipitate was collected via filtration to afford the mixture of **3e** and **3e′** (257 mg, 93%) as a reddish-brown solid. M.p. 171 °C (decomp.); IR (ATR): ν_max_ = 1645 (C=O) cm^−1^; ^1^H NMR (500 MHz, DMSO-*d*_6_): δ_H_ = 12.19 (s), 9.53–9.55 (m), 8.24–8.33 (m), 7.50–7.58 (m), 7.38–7.44 (m), 7.14 (d), 7.09 (s), 7.00–7.03 (m), 6.92 (dd), 6.80 (d), 3.86 (d) 2.47 (t) ppm; ^13^C NMR (125 MHz, DMSO-*d*_6_): δ_C_ = 175.7, 175.2, 162.8, 161.2, 151.8, 150.7, 144.8, 144.5, 143.0, 142.3, 139.9, 134.8, 134.5, 134.4, 133.1, 131.9, 131.8, 130.1, 128.7, 128.1, 118.3, 113.8, 111.3, 110.3, 110.1, 104.6, 104.0, 103.4, 99.8, 56.4, 56.0 ppm; HRMS (ESI-TOF): calcd for C_18_H_13_NO_2_ + H^+^ [M + H]^+^ 276.1019; found: 276.1018.

2-Iodoazuleno[2,1-*b*]quinolin-12(5*H*)-one (**3f**): To a solution of **2f** (490 mg, 1.00 mmol) in polyphosphoric acid (10 mL) was stirred at 140 °C for 4.5 h. After the reaction mixture was cooled, it was poured into water. The generated precipitate was collected via filtration to afford **3f** (309 mg, 63%) as a reddish-brown solid. M.p. 240 °C (decomp.); IR (ATR): ν_max_ = 1622 (C=O) cm^−1^; ^1^H NMR (500 MHz, DMSO-*d*_6_): δ_H_ = 12.41 (s, 1H, NH), 9.52–9.50 (m, 1H, H_11_), 8.59 (d, 1H, *J* = 2.0 Hz, H_1_), 8.35 (d, 1H, *J* = 10.6 Hz, H_7_), 7.95 (dd, 1H, *J* = 8.6, 2.0 Hz, H_3_), 7.61–7.59 (m, 2H, H_4,9_), 7.48–7.42 (m, 2H, H_8,10_), 7.10 (s, 1H, H_6_) ppm; ^13^C NMR (125 MHz, DMSO-*d*_6_): δ_C_ = 173.9, 151.5, 145.2, 140.2, 140.0, 139.6, 135.3, 134.9, 134.7, 132.4, 130.7, 129.3, 125.9, 120.5, 110.3, 104.3, 85.8 ppm; HRMS (ESI-TOF): calcd for C_17_H_10_NOI + H^+^ [M + H]^+^ 371.9880; found: 371.9889.

Azuleno[2,1-*b*]benzo[*h*]quinolin-7(14*H*)-one (**3g**): To a solution of **2g** (416 mg, 1.01 mmol) in polyphosphoric acid (10 mL) was stirred at 140 °C for 2.5 h. After the reaction mixture was cooled, it was poured into water. The generated precipitate was collected via filtration to afford **3g** (297 mg, 100%) as a reddish-brown solid. M.p. 208 °C (decomp.); IR (ATR): ν_max_ = 1631 (C=O) cm^−1^; ^1^H NMR (500 MHz, DMSO-*d*_6_): δ_H_ =12.60 (s, 1H, NH), 9.65 (d, 1H, *J* = 9.7 Hz, H_8_), 8.93 (d, 1H, *J* = 8.0 Hz, H_6_), 8.47 (d, 1H, *J* = 10.3 Hz, H_12_), 8.42 (d, 1H, *J* = 8.6 Hz, H_4_), 8.08 (d, 1H, *J* = 7.4 Hz, H_1_), 7.80–7.74 (m, 3H, H_2,3,10_), 7.68–7.61 (m, 2H, H_5,9_), 7.48 (t, 1H, *J* = 10.0 Hz, H_11_), 7.39 (s, 1H, H_13_) ppm; ^13^C NMR (125 MHz, DMSO-*d*_6_): δ_C_ = 175.3, 151.1, 144.1, 139.5, 137.3, 135.6, 135.4, 135.2, 132.8, 130.1, 129.2, 128.9, 128.6, 127.0, 123.9, 123.2, 122.9, 122.4, 120.4, 111.4, 105.2 ppm; HRMS (ESI-TOF): calcd for C_21_H_13_NO + H^+^ [M + H]^+^ 296.1070; found: 296.1084.

12-Chloroazuleno[2,1-*b*]quinoline (**4a**): To a solution of **3a** (613 mg, 2.50 mmol) was added POCl_3_ (20 mL) and the resulting mixture was refluxed for 2 h. The reaction mixture was diluted with MeCN, poured into ice-cooled aq. NaOH, and generated precipitate was collected via filtration. The residue was purified by silica gel column chromatography with CHCl_3_/AcOEt (5:1) as the eluent to afford **4a** (590 mg, 89%) as a greenish-brown solid. M.p. 206–207 °C; ^1^H NMR (500 MHz, CDCl_3_): δ_H_ = 9.00–8.98 (m, 1H, H_11_), 8.59 (dd, *J* = 8.6, 0.9 Hz, 1H, H_1_), 8.28 (d, *J* = 8.6 Hz, 1H, H_4_), 7.89–7.85 (m, 1H, H_3_), 7.75 (d, *J* = 11.2 Hz, 1H, H_7_), 7.68–7.65 (m, 1H, H_2_), 7.27 (s, 1H, H_6_), 7.04–7.02 (m, 2H, H_9,10_), 6.85–6.81 (m, 1H, H_8_) ppm; ^13^C NMR (125 MHz, CDCl_3_): δ_C_ = 159.9, 148.7, 147.8, 139.5, 137.8, 135.1, 134.8, 131.9, 130.2, 128.7, 128.5, 127.9, 124.9, 124.6, 123.0, 121.2, 117.6 ppm; HRMS (MALDI-TOF): calcd for C_17_H_10_NCl + H^+^ [M + H]^+^ 264.0575; found: 264.0586.

12-Chloro-2-methylazuleno[2,1-*b*]quinoline (**4b**): To a solution of **3b** (130 mg, 0.503 mmol) was added POCl_3_ (5 mL) and the resulting mixture was refluxed for 2 h. The reaction mixture was diluted with MeCN, poured into ice-cooled aq. NaOH, and generated precipitate was collected via filtration. The residue was purified by silica gel column chromatography with CHCl_3_/AcOEt (5:1) as the eluent to afford **4b** (132 mg, 94%) as a greenish-brown solid. M.p. 172–174 °C; ^1^H NMR (500 MHz, CDCl_3_): δ_H_ = 8.93 (m, 1H, H_11_), 8.31 (s, 1H, H_1_), 8.16 (d, 1H, *J* = 8.6 Hz, H_4_), 7.67–7.72 (m, 2H, H_3,7_), 7.23 (s, 1H, H_6_), 6.97–6.99 (m, 2H, H_8,10_), 6.78 (m, 1H, H_9_), 2.65 (s, 3H, Me) ppm; ^13^C NMR (125 MHz, CDCl_3_): δ_C_ = 159.6, 147.7, 147.4, 139.8, 137.2, 135.4, 135.0, 134.9, 132.7, 131.9, 128.7, 128.4, 127.6, 123.4, 123.1, 121.3, 117.8, 22.0 ppm; HRMS (MALDI-TOF): calcd for C_18_H_12_NCl + H^+^ [M + H]^+^ 278.0731; found: 278.0737.

2-Butyl-12-chloroazuleno[2,1-*b*]quinoline (**4c**): To a solution of **3c** (200 mg, 0.664 mol) was added POCl_3_ (7 mL) and the resulting mixture was refluxed for 2 h. The reaction mixture was diluted with MeCN, poured into ice-cooled aq. NaOH, and generated precipitate was collected via filtration. The residue was purified by silica gel column chromatography with CHCl_3_/AcOEt (5:1) as the eluent to afford **4c** (197 mg, 93%) as a greenish-brown solid. M.p. 126–128 °C; ^1^H NMR (500 MHz, CDCl_3_): δ_H_ = 8.94–8.92 (m, 1H, H_11_), 8.31 (d, 1H, *J* = 1.1 Hz, H_1_), 8.18 (d, 1H, *J* = 8.6 Hz, H_4_), 7.71 (m, 2H, H_3,7_), 7.23 (s, 1H, H_6_), 7.00–6.94 (m, 2H, H_8,10_), 6.79–6.75 (m, 1H, H_9_), 2.91 (t, 2H, *J* = 7.7 Hz, *n*-Bu), 1.77 (sext., 2H, *J* = 7.7 Hz, *n*-Bu), 1.45 (sept., 2H, *J* = 7.7 Hz, *n*-Bu), 0.99 (t, 3H, *J* = 7.7 Hz, *n*-Bu) ppm; ^13^C NMR (125 MHz, CDCl_3_): δ_C_ = 159.6, 147.9, 147.4, 139.9, 137.4, 135.3, 134.4, 132.0, 128.8, 128.6, 128.0, 123.1, 122.8, 121.3, 118.3, 117.8, 117.4, 36.1, 33.8, 22.6, 14.1 ppm; HRMS (MALDI-TOF): calcd for C_21_H_18_NCl + H^+^ [M + H]^+^ 320.1201; found: 320.1190.

12-Chloro-2-methoxyazuleno[2,1-*b*]quinoline (**4d**): To a solution of **3d** (228 mg, 0.829 mmol) was added POCl_3_ (8 mL) and the resulting mixture was refluxed for 2 h. The reaction mixture was diluted with MeCN, poured into ice-cooled aq. NaOH, and generated precipitate was collected via filtration. The residue was purified by silica gel column chromatography with CHCl_3_/AcOEt (5:1) as the eluent to afford **4d** (202 mg, 83%) as a greenish-brown solid. M.p. 201–203 °C; ^1^H NMR (500 MHz, CDCl_3_): δ_H_ = 8.90 (d, 1H, *J* = 8.0 Hz, H_11_), 8.15 (d, 1H, *J* = 9.2 Hz, H_4_), 7.74 (d, 1H, *J* = 2.9 Hz, H_1_), 7.70 (d, 1H, *J* = 11.5 Hz, H_7_), 7.50 (dd, 1H, *J* = 9.2, 2.6 Hz, H_3_), 7.22 (s, 1H, H_6_), 7.00–6.93 (m, 2H, H_8,10_), 6.79–6.75 (m, 1H, H_9_), 4.02 (s, 3H, OMe) ppm; ^13^C NMR (125 MHz, CDCl_3_): δ_C_ = 158.4, 157.1, 146.4, 145.2, 139.5, 136.2, 135.5, 135.0, 132.1, 130.4, 128.1, 127.2, 124.0, 123.5, 121.3, 117.7, 102.1, 55.8 ppm; HRMS (MALDI-TOF): calcd for C_18_H_12_NOCl + H^+^ [M + H]^+^ 294.0680; found: 294.0699.

Reaction of **3e** and **3e′**: To a solution of **3e** and **3e′** (201 mg, 0.730 mmol) was added POCl_3_ (7 mL) and the resulting mixture was refluxed for 2 h. The reaction mixture was diluted with MeCN, poured into ice-cooled aq. NaOH, and generated precipitate was collected via filtration. The residue was purified by silica gel column chromatography with CHCl_3_/AcOEt (5:1) as the eluent to afford the mixture of **4e** and **4e′** (156 mg, 72%) as a greenish-brown solid. M.p. 191–195 °C; ^1^H NMR (500 MHz, CDCl_3_): δ_H_ = 9.17 (m), 8.97–8.99 (m), 8.45 (d), 7.87 (dd, 7.79 (d), 7.70–7.75 (m), 7.58 (d), 7.29 (dd), 7.21–7.25 (m), 7.01–7.07 (m), 6.97 (d), 6.80–6.89 (m), 4.06 (s), 4.02 (s) ppm; ^13^C NMR (125 MHz, CDCl_3_): δ_C_ = 161.8, 160.4, 159.7, 157.7, 150.9, 150.7, 148.2, 147.6, 140.4, 139.6, 138.1, 135.2, 135.2, 134.8, 134.7, 132.3, 131.5, 130.2, 128.7, 128.5, 127.9, 127.9, 125.9, 121.9, 121.8, 119.4, 118.4, 118.3, 117.1, 117.0, 115.3, 106.3, 105.1, 56.2, 55.8 ppm; HRMS (MALDI-TOF): calcd for C_18_H_12_NOCl + H^+^ [M + H]^+^ 294.0680; found: 294.0699.

12-Chloro-2-iodoazuleno[2,1-*b*]quinoline (**4f**): To a solution of **3f** (259 mg, 0.698 mmol) was added POCl_3_ (7 mL) and the resulting mixture was refluxed for 2 h. The reaction mixture was diluted with MeCN, poured into ice-cooled aq. NaOH, and generated precipitate was collected via filtration. The residue was purified by silica gel column chromatography with CHCl_3_/AcOEt (5:1) as the eluent to afford **4f** (165 mg, 61%) as a greenish-brown solid. M.p. 201–203 °C; ^1^H NMR (500 MHz, CDCl_3_): δ_H_ = 8.92 (dd, 1H, *J* = 9.2, 1.1 Hz, H_11_), 8.88 (d, 1H, *J* = 1.7 Hz, H_1_), 8.04 (dd, 1H, *J* = 8.9, 2.0 Hz, H_4_), 7.96 (d, 1H, *J* = 8.9 Hz, H_3_), 7.73 (d, 1H, *J* = 11.5 Hz, H_7_), 7.20 (s, 1H, H_6_), 7.07–7.00 (m, 2H, H_8,10_), 6.86–6.82 (m, 1H, H_9_) ppm; ^13^C NMR (125 MHz, CDCl_3_): δ_C_ = 158.1, 149.1, 148.0, 140.3, 137.8, 136.4, 135.1, 130.6, 129.8, 129.6, 129.5, 125.6, 124.6, 123.9, 120.6, 84.4 ppm, one signal is overlapped with other signal; HRMS (ESI-TOF): calcd for C_17_H_9_NICl + H^+^ [M + H]^+^ 389.9541; found: 389.9540.

7-Chloroazuleno[2,1-*b*]benzo[h]quinoline (**4g**): To a solution of **3g** (246 mg, 0.823 mmol) was added POCl_3_ (8 mL) and the resulting mixture was refluxed for 2 h. The reaction mixture was diluted with MeCN, poured into ice-cooled aq. NaOH, and generated precipitate was collected via filtration. The residue was purified by silica gel column chromatography with CHCl_3_/AcOEt (5:1) as the eluent to afford **4g** (206 mg, 80%) as a greenish-brown solid. M.p. 209–210 °C; ^1^H NMR (500 MHz, CDCl_3_): δ_H_ = 9.55 (d, 1H, *J* = 7.7 Hz, H_1_), 9.12 (dd, 1H, *J* = 8.3, 1.1 Hz, H_8_), 8.39 (d, 1H, *J* = 9.2 Hz, H_6_), 7.94–7.90 (m, 2H, H_3,12_), 7.83 (d, 1H, *J* = 9.2 Hz, H_5_), 7.79–7.72 (m, 2H, H_2,4_), 7.48 (s, 1H, H_13_), 7.16–7.06 (m, 2H, H_9,11_), 6.90 (m, 1H, H_10_) ppm; ^13^C NMR (125 MHz, CDCl_3_): δ_C_ = 147.8, 146.1, 139.0, 137.8, 135.9, 135.5, 133.8, 132.9, 131.2, 128.9, 128.0, 127.8, 127.1, 127.1, 126.3, 125.8, 121.7, 120.6, 120.5, 117.6 ppm, one signal is overlapped with other signal; HRMS (ESI-TOF): calcd for C_21_H_12_NCl + H^+^ [M + H]^+^ 314.0731; found: 314.0749.

12-(Pyrrolidin-1-yl)azuleno[2,1-*b*]quinoline (**5a**): A solution of **4a** (136 mg, 0.516 mmol) in pyrrolidine (3 mL) was stirred at room temperature for 1 h. The amine was removed under reduced pressure. The crude product was purified by silica gel column chromatography with CHCl_3_/AcOEt (5:1) as the eluent to afford **5a** (131 mg, 85%) as a brown oil. ^1^H NMR (500 MHz, CDCl_3_): δ_H_ = 8.24 (d, *J* = 8.7 Hz, 1H, H_11_), 8.19–8.15 (m, 2H, H_2,4_), 7.72–7.65 (m, 2H, H_1,7_), 7.43–7.39 (m, 1H, H_3_), 7.21 (s, 1H, H_6_), 6.92–6.88 (m, 2H, H_8,10_), 6.74 (dd, *J* = 11.1, 7.2 Hz, 1H, H_9_), 3.66–3.62 (m, 4H, pyrrolidine), 2.21–2.18 (m, 4H, pyrrolidine)ppm; ^13^C NMR (125 MHz, CDCl_3_): δ_C_ = 161.3, 150.8, 150.5, 146.5, 140.5, 134.4, 132.9, 129.4, 129.3, 129.1, 128.6, 127.2, 124.5, 123.0, 122.4, 120.0, 117.7, 52.8, 26.7 ppm; HRMS (ESI-TOF): calcd for C_21_H_18_N_2_ + H^+^ [M + H]^+^ 299.1543; found: 299.1545.

12-(Piperidin-1-yl)azuleno[2,1-*b*]quinoline (**5b**): A solution of **4a** (132 mg, 0.501 mmol) in piperidine (3 mL) was stirred at 50 °C for 4 h. The amine was removed under reduced pressure. The crude product was purified by silica gel column chromatography with CHCl_3_/AcOEt (5:1) as the eluent to afford **5b** (122 mg, 78%) as a brown oil. ^1^H NMR (500 MHz, CDCl_3_): δ_H_ = 9.03 (d, 1H, *J* = 8.0 Hz, H_11_), 8.44 (dd, 1H, *J* = 8.6, 0.9 Hz, H_4_), 8.29 (dd, 1H, *J* = 8.4, 0.7 Hz, H_2_), 7.76–7.69 (m, 2H, H_1,7_), 7.49–7.46 (m, 1H, H_3_), 7.25 (s, 1H, H_6_), 6.99–6.91 (m, 2H, H_8,10_), 6.76 (m, 1H, H_9_), 3.54 (t, 4H, *J* = 5.2 Hz, piperidine), 1.92–1.84 (m, 6H, piperidine) ppm; ^13^C NMR (125 MHz, CDCl_3_): δ_C_ = 161.5, 154.2, 150.5, 147.2, 140.7, 134.9, 133.8, 130.7, 129.6, 129.2, 128.6, 127.4, 125.6, 125.1, 122.8, 120.7, 118.1, 52.7, 27.0, 24.6 ppm; HRMS (ESI-TOF): calcd for C_22_H_20_N_2_ + H^+^ [M + H]^+^ 313.1699; found: 313.1690.

12-(Morpholin-4-yl)azuleno[2,1-*b*]quinoline (**5c**): A solution of **4a** (135 mg, 0.512 mmol) in morpholine (3 mL) was stirred at 50 °C for 4 h. The amine was removed under reduced pressure. The crude product was purified by silica gel column chromatography with CHCl_3_/AcOEt (5:1) as the eluent to afford **5c** (118 mg, 74%) as a brown solid. M.p. 130–132 °C; ^1^H NMR (500 MHz, CDCl_3_): δ_H_ = 9.21–9.19 (m, 1H, H_11_), 8.49 (d, 1H, *J* = 8.6 Hz, H_4_), 8.32–8.30 (m, 1H, H_2_), 7.80–7.77 (m, 1H, H_1_), 7.73 (d, 1H, *J* = 11.2 Hz, H_7_), 7.55–7.52 (m, 1H, H_3_), 7.27 (s, 1H, H_6_), 7.02–6.97 (m, 2H, H_8,10_), 6.82–6.78 (m, 1H, H_9_), 4.09 (t, 4H, *J* = 4.4 Hz, morpholine), 3.65 (t, 4H, *J* = 4.4 Hz, morpholine) ppm; ^13^C NMR (125 MHz, CDCl_3_): δ_C_ = 161.5, 152.0, 150.7, 147.4, 140.6, 135.1, 134.3, 130.9, 129.9, 129.4, 128.6, 127.6, 125.1, 124.6, 123.3, 121.3, 118.4, 68.0, 51.0 ppm, one signal is overlapped with other signal; HRMS (ESI-TOF): calcd for C_21_H_18_N_2_O + H^+^ [M + H]^+^ 315.1492; found: 315.1490.

## 4. Conclusions

In conclusion, we have demonstrated herein a procedure for the synthesis of azuleno [2,1-*b*]quinolones and quinolines from 2-arylaminoazulene derivatives, which are prepared via the S_N_Ar reaction of 2-chloroazulenes with arylamines. The synthesis of azuleno [2,1-*b*]quinolones was successfully accomplished through the intramolecular cyclization of 2-arylaminoazulene derivatives bearing ester groups, employing PPA as a Brønsted acid catalyst. Furthermore, halogenative aromatization of the corresponding azuleno [2,1-*b*]quinolones with POCl_3_ afforded azuleno [2,1-*b*]quinolines with a chlorine substituent. Additionally, the S_N_Ar reaction of azuleno [2,1-*b*]quinolines with several secondary amines was also investigated. Consequently, the reactions proceeded expeditiously under the low-temperature conditions, yielding the respective aminoquinoline derivatives, except for the reaction with diethylamine.

As discussed in the Introduction section, quinolones and quinolines possess considerable potential in the fields of pharmaceuticals and medicinal chemistry. Therefore, the exploration of additional avenues to functionalize these derivatives may lead to practical pharmaceutical agents. The development of an additional synthetic methodology for azuleno [2,1-*d*]quinolones and quinolines is currently underway in our laboratory.

## Figures and Tables

**Table 1:** Synthesis of 2-Arylaminoazulenes **2a**–**2i** via S_N_Ar reaction of 2-Chloroazulene derivative **1** with anilines.

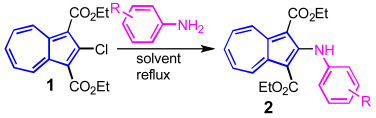				
Entry	Aniline Derivative	Solvent	Product	Yield ^a^
1	aniline	EtOH	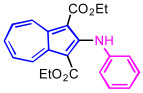	**2a**, 96%
2	*p*-toluidine	EtOH	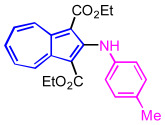	**2b**, 89%
3	*p*-*n*-butylaniline	EtOH	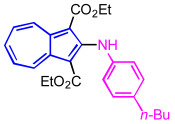	**2c**, 89%
4	*p*-methoxyaniline	EtOH	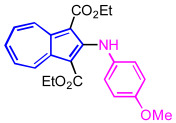	**2d**, 96%
5	*m*-methoxyaniline	EtOH	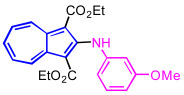	**2e**, 92%
6	*p*-iodoaniline	EtOH	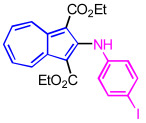	**2f**, 72%
7	*p*-nitroaniline	NMP	No reaction	
8	α-naphthylamine	EtOH	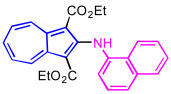	**2g**, 61%
9	*N*-methylaniline	without solvent	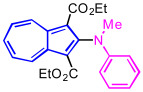	**2h**, 33%

^a^ Isolated yield.

**Table 2:** Intramolecular Cyclization of **2a**–**2g** into Azuleno[2,1-*b*]quinolones **3a**–**3g**.

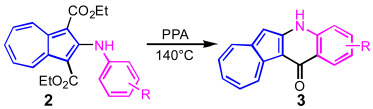			
Entry	Substrate	Product	Yield ^a^
1	**2a**	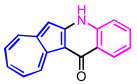	**3a**, 96%
2	**2b**	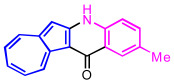	**3b**, 85%
3	**2c**	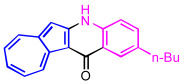	**3c**, 86%
4	**2d**	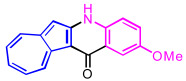	**3d**, 85%
5	**2e**	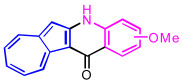	**3e** + **3e′**, 93% ^b^
6	**2f**	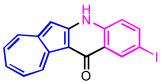	**3f**, 89%
7	**2g**	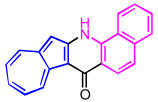	**3g**, 100%

^a^ Isolated yield. ^b^ inseparable mixture.

**Table 3:** Synthesis of Azuleno[2,1-*b*]quinolines **4a**–**4h** via halogenative aromatization with POCl_3_.

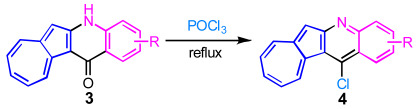			
Entry	Substrate	Product	Yield ^a^
1	**3a**	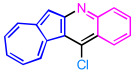	**4a**, 89%
2	**3b**	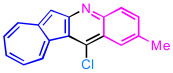	**4b**, 94%
3	**3c**	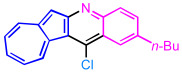	**4c**, 93%
4	**3d**	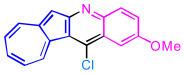	**4d**, 83%
5	**3e** + **3e′**	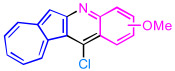	**4e** + **4e′**, 72% ^b^
6	**3f**	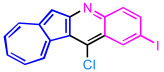	**4f**, 61%
7	**3g**	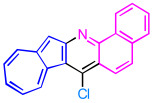	**4g**, 80%

^a^ Isolated yield. ^b^ inseparable mixture.

## Data Availability

Data are available from the corresponding author.

## Supplementary Materials

The following supporting information can be downloaded at: https://www.mdpi.com/article/10.3390/molecules28155785/s1, Figures: ^1^H and ^13^C NMR spectra of new compounds.

## Sample Availability

Not available.
