# Supplementary material for: Synthesis of Azuleno[2,1-b]quinolones and Quinolines via Brønsted Acid-Catalyzed Cyclization of 2-Arylaminoazulenes
Source: Molecules. 2023 Jul 31;28(15):5785. doi: 10.3390/molecules28155785 (PMC10420867; doi:10.3390/molecules28155785)
Supplement: Supplementary file 1 [file molecules-28-05785-s001.zip › molecules-2518411-supplementary.pdf]

molecules

MDPI

Supplementary Materials

Synthesis of Azuleno[2,1-*b*]quinolones and Quinolines via Brønsted Acid-catalyzed Cyclization of 2-Arylaminoazulenes

Taku Shoji *, Mutsumi Takeuchi, Mayumi Uda, Yukino Ariga, Akari Yamazaki and Ryuta Sekiguchi, Shunji Ito

➢ *Contents*

1. Copies of ^{1}H NMR and ^{13}C NMR spectra of reported compounds. Figures S1−S50

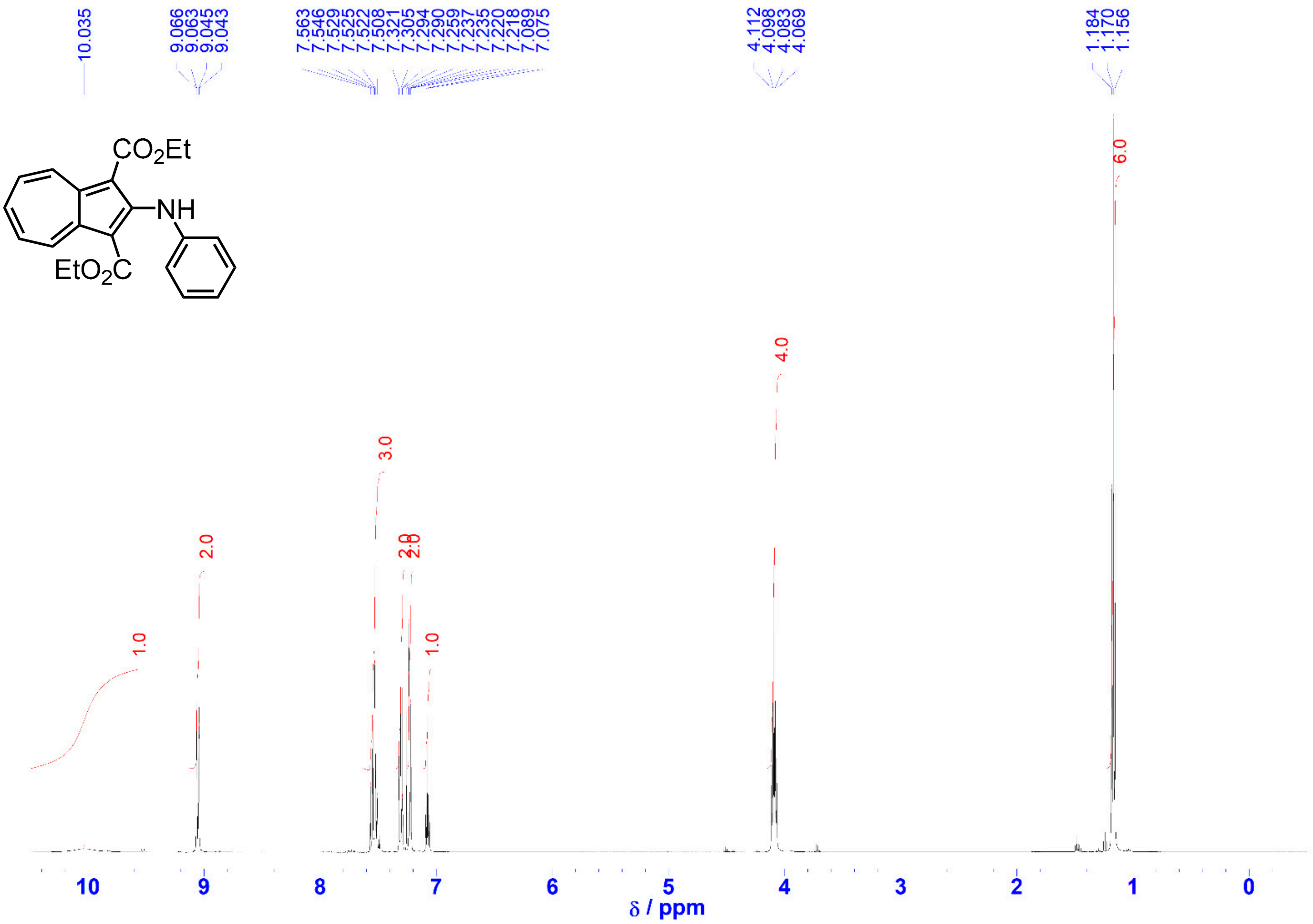

Figure S1. ^{1}H NMR spectrum of **2a** in $CDCl_3$ (500 MHz).

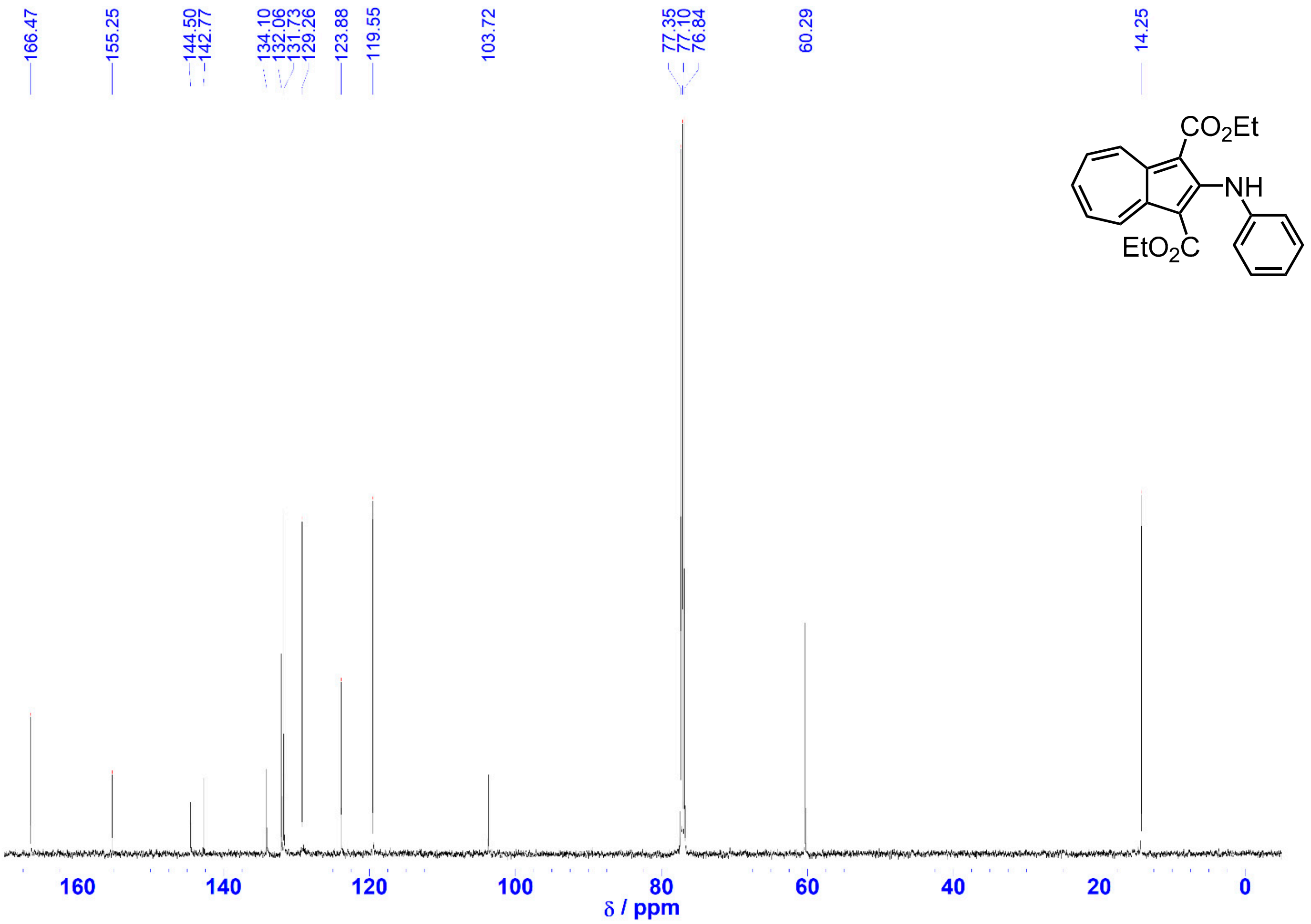

Figure S2. ^{13}C NMR spectrum of **2a** in $CDCl_3$ (125 MHz).

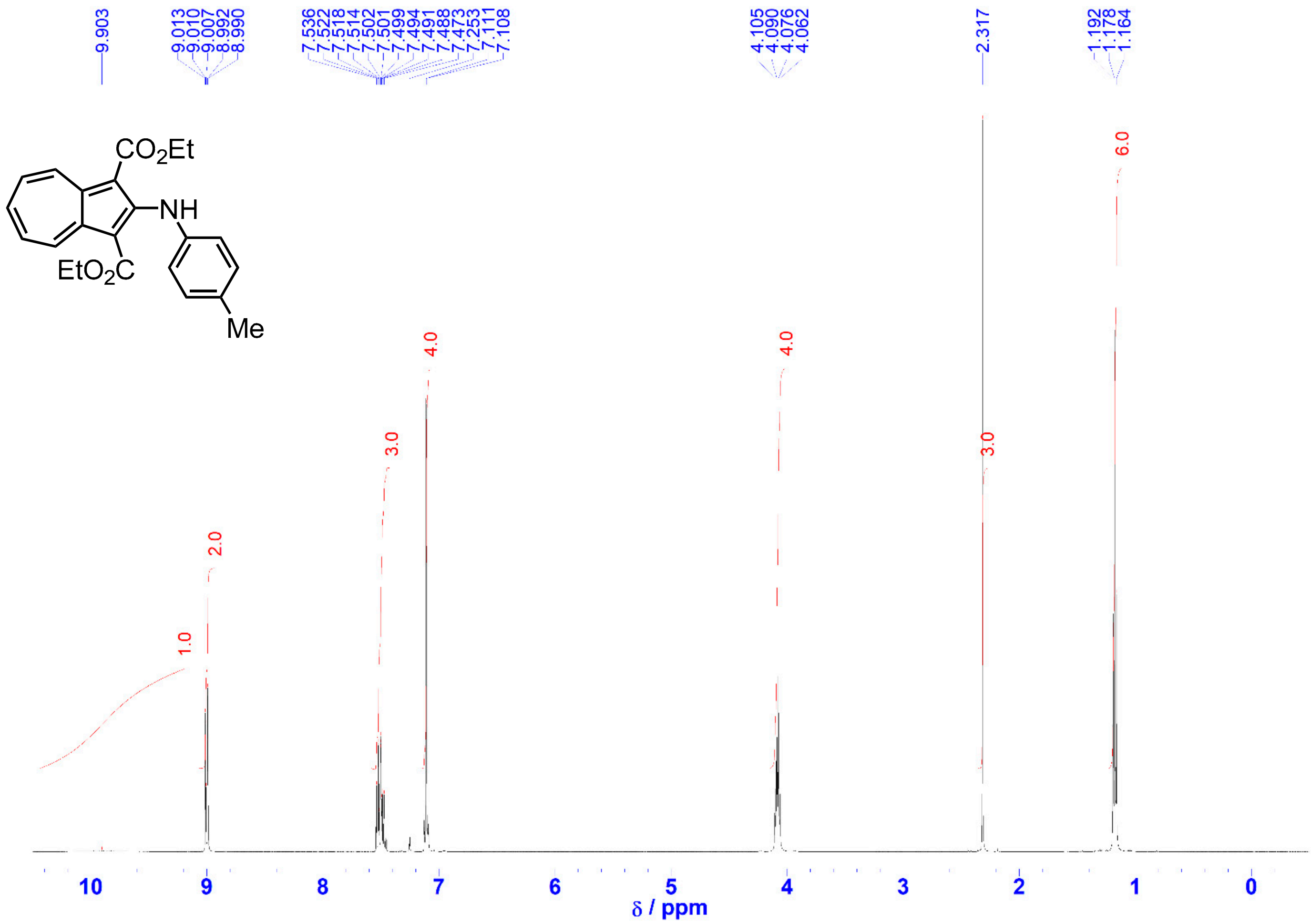

Figure S3. ^{1}H NMR spectrum of **2b** in $CDCl_3$ (500 MHz).

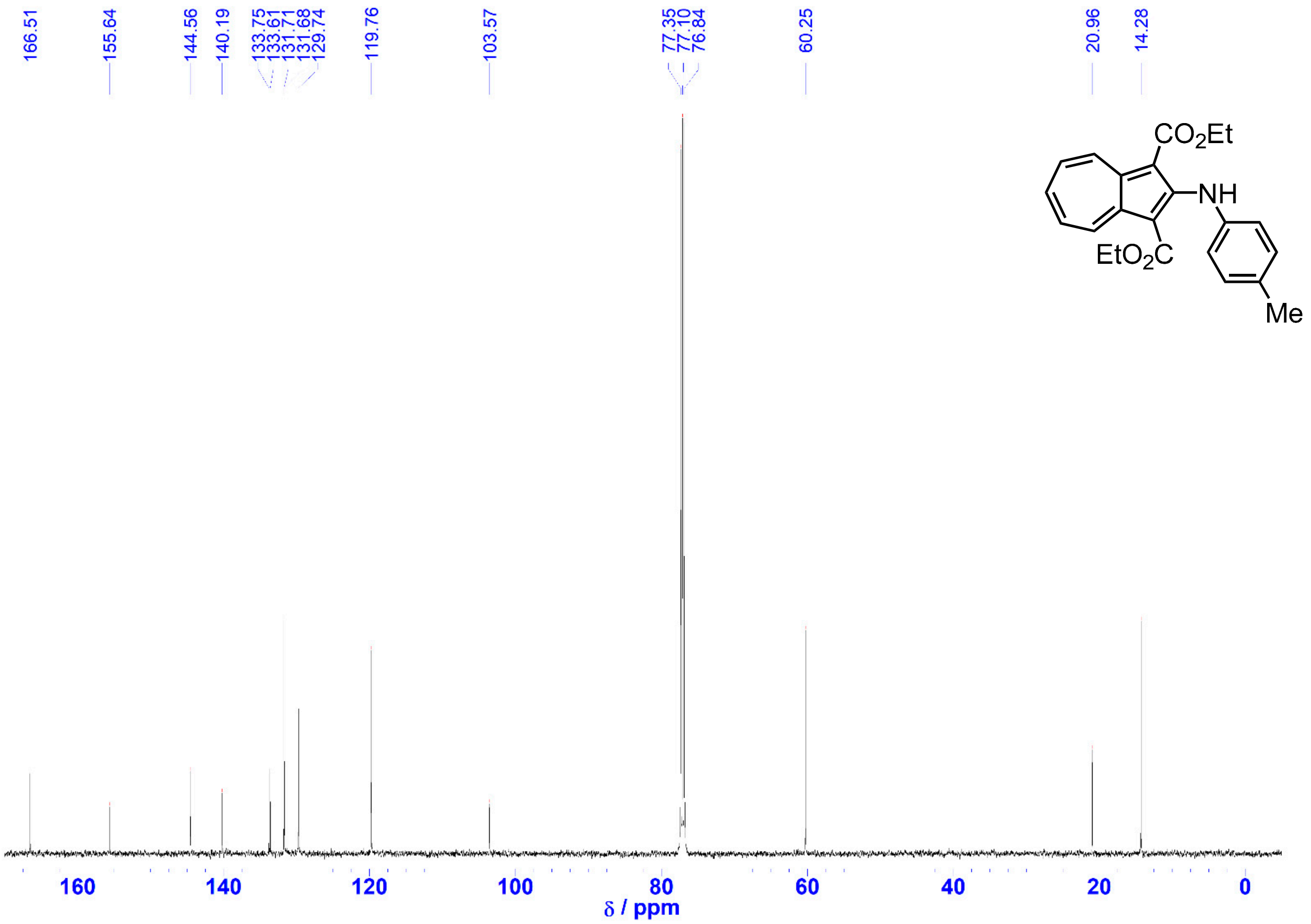

Figure S4. ^{13}C NMR spectrum of **2b** in $CDCl_3$ (125 MHz).

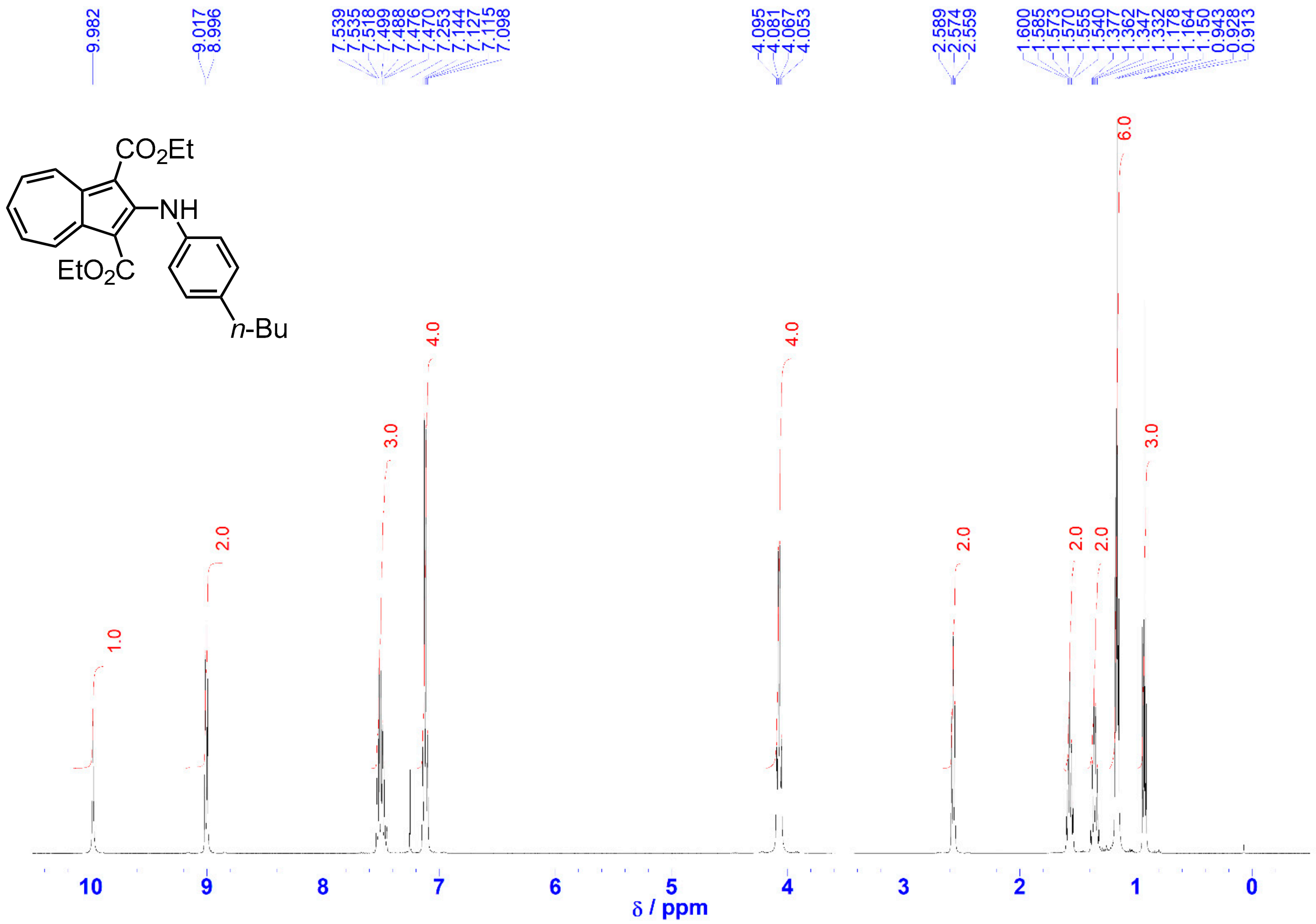

Figure S5. ^{1}H NMR spectrum of **2c** in $CDCl_3$ (500 MHz).

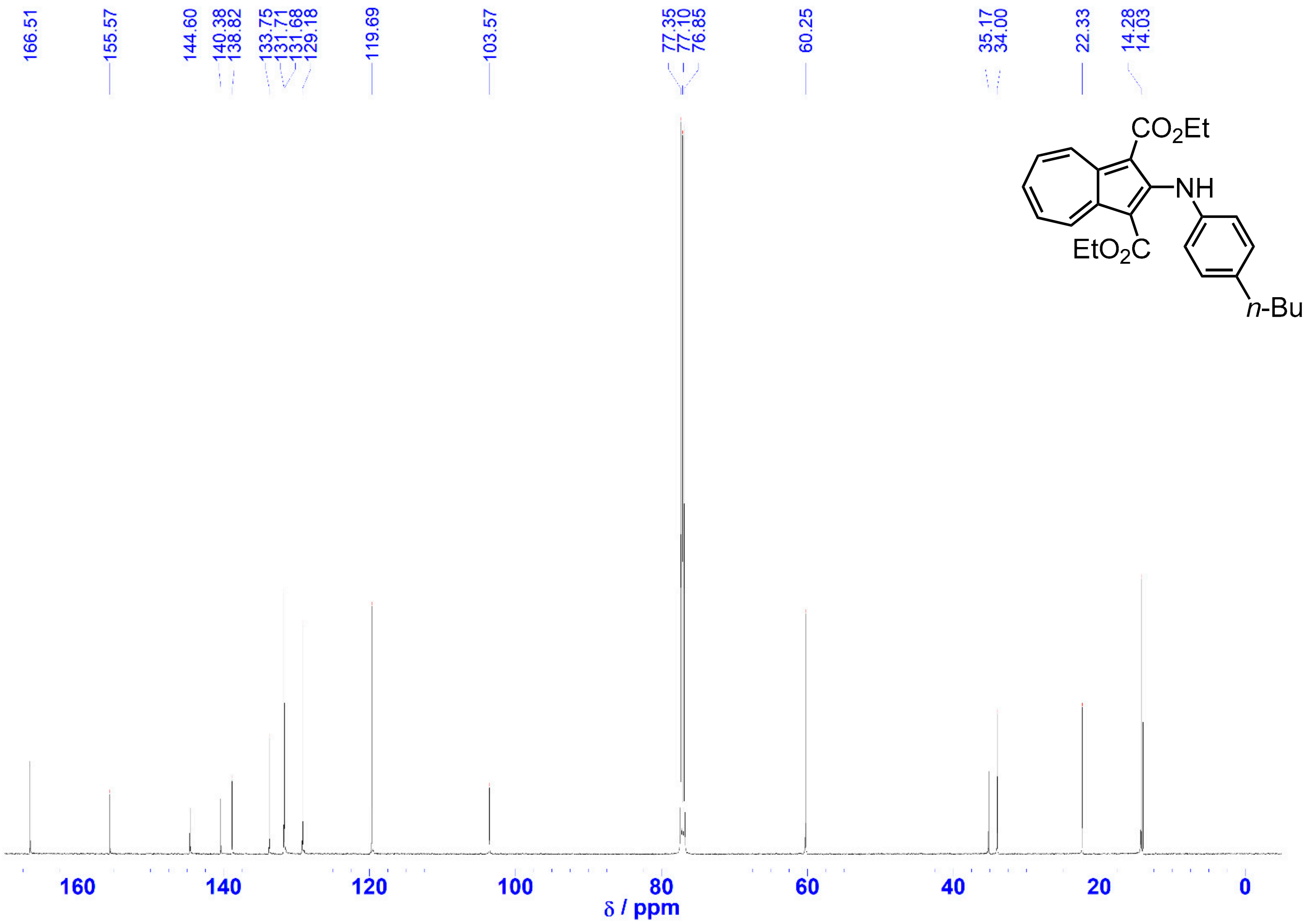

Figure S6. ^{13}C NMR spectrum of **2c** in $CDCl_3$ (125 MHz).

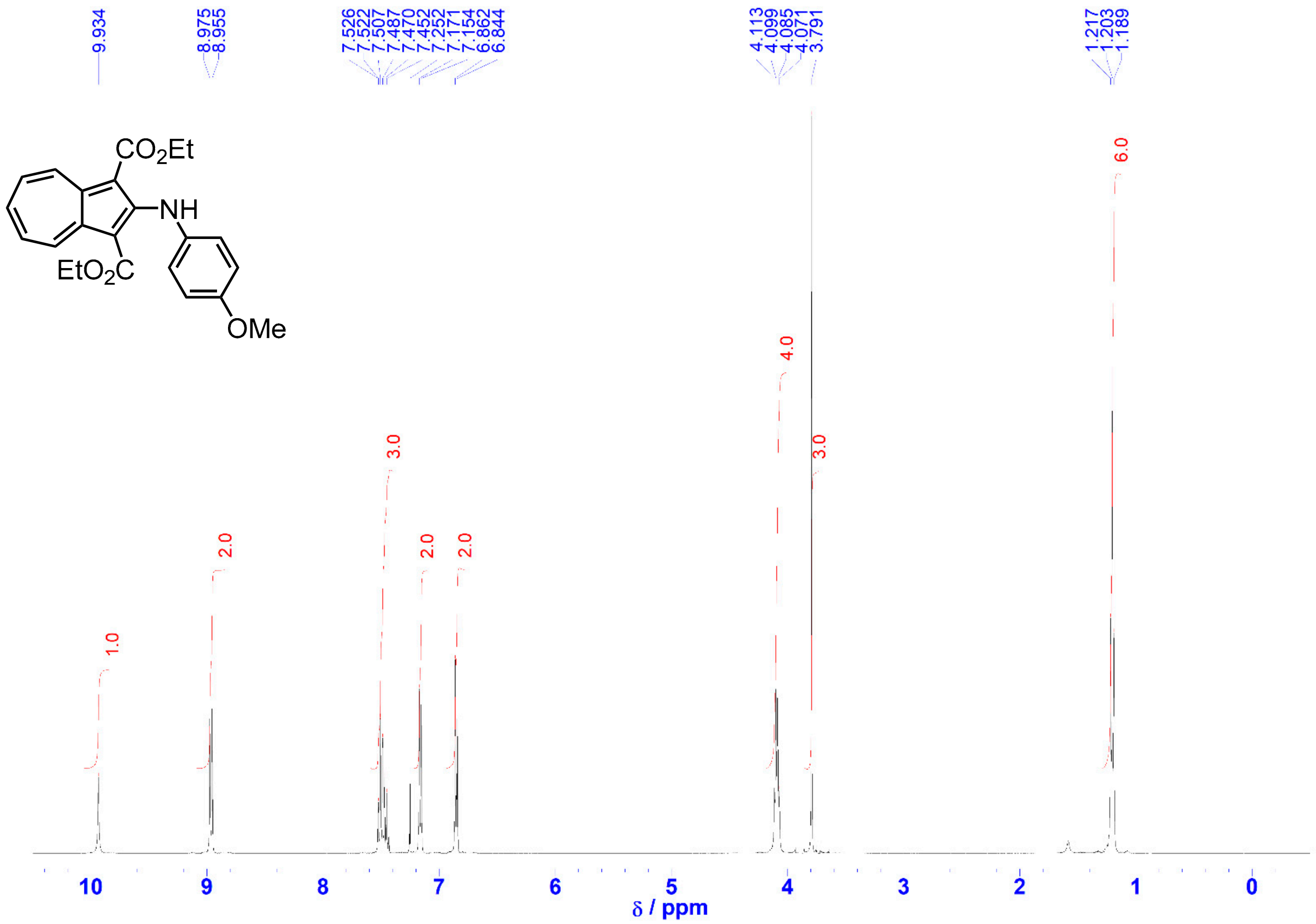

Figure S7. ^{1}H NMR spectrum of **2d** in $CDCl_3$ (500 MHz).

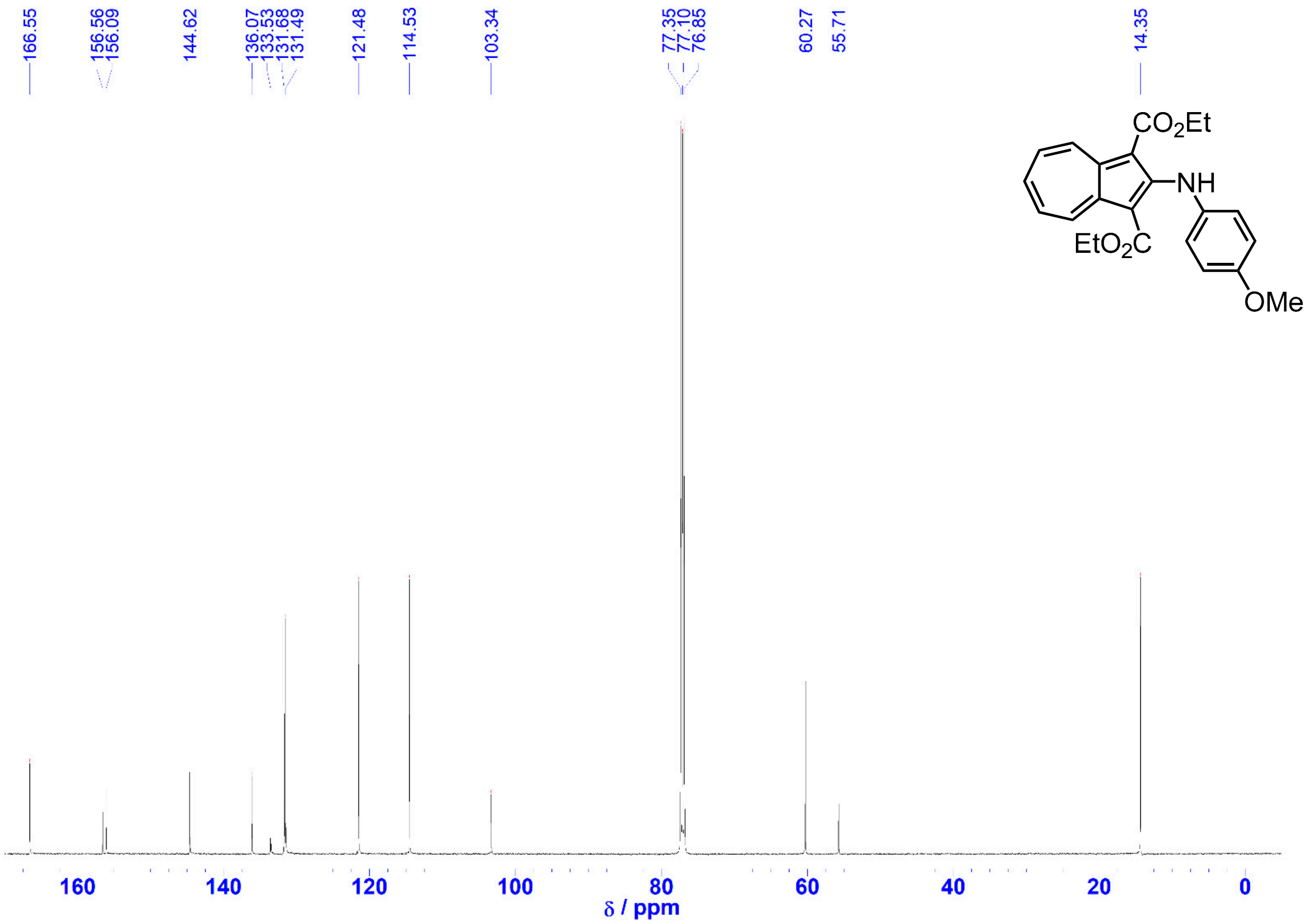

Figure S8. ^{13}C NMR spectrum of **2d** in $CDCl_3$ (125 MHz).

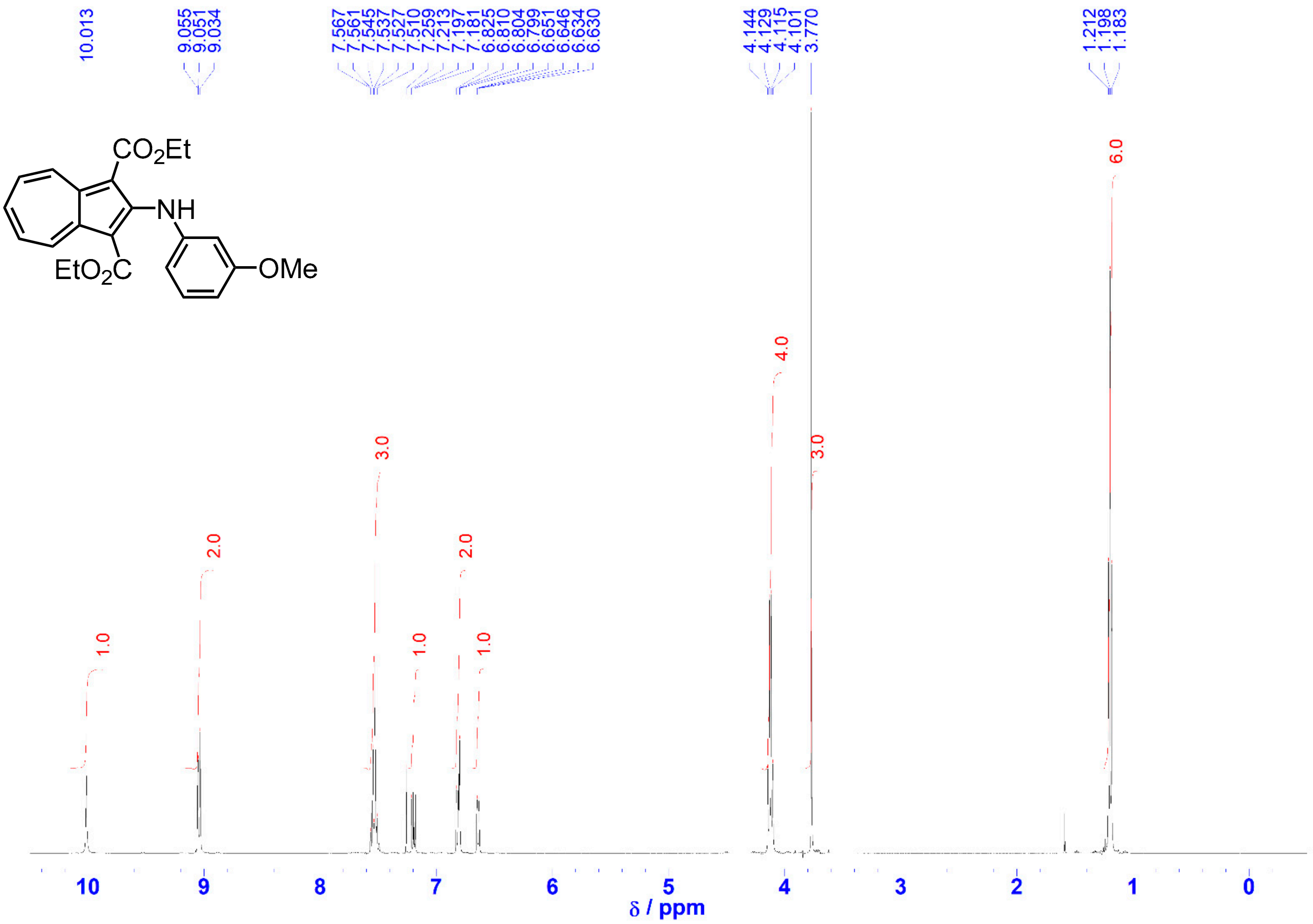

Figure S9. ^{1}H NMR spectrum of **2e** in $CDCl_3$ (500 MHz).

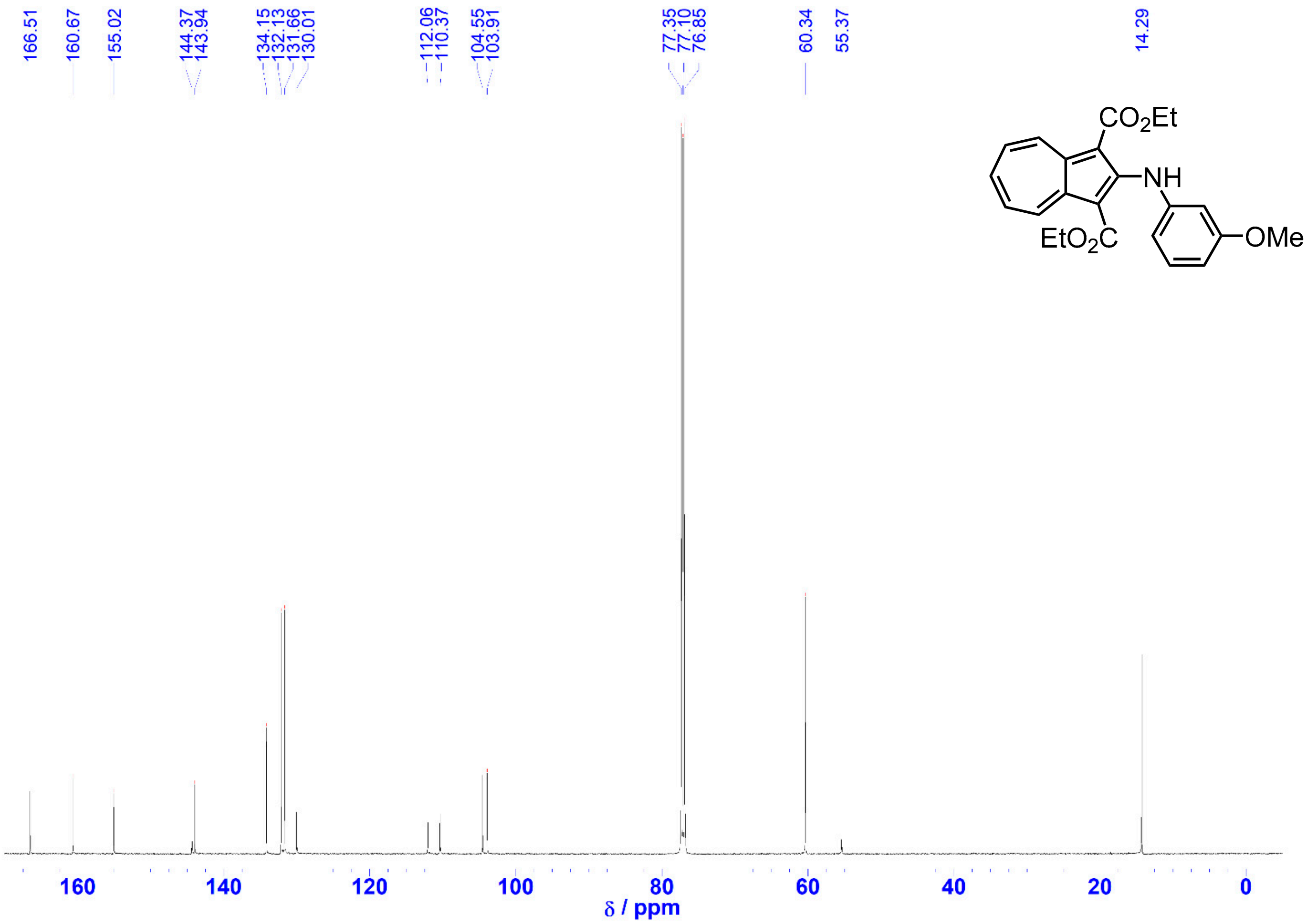

Figure S10. ^{13}C NMR spectrum of **2e** in $CDCl_3$ (125 MHz).

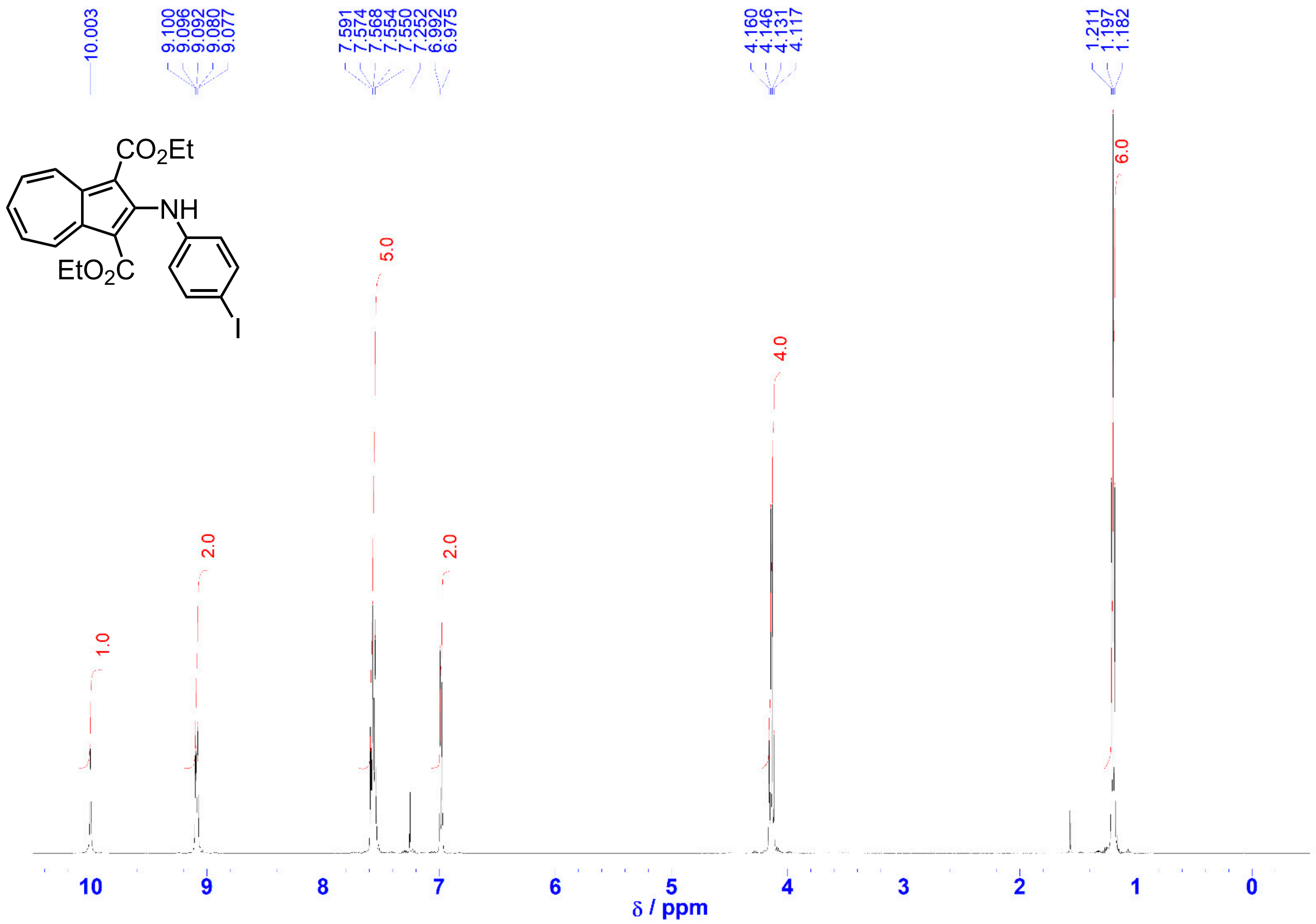

Figure S11. ^{1}H NMR spectrum of **2f** in $CDCl_3$ (500 MHz).

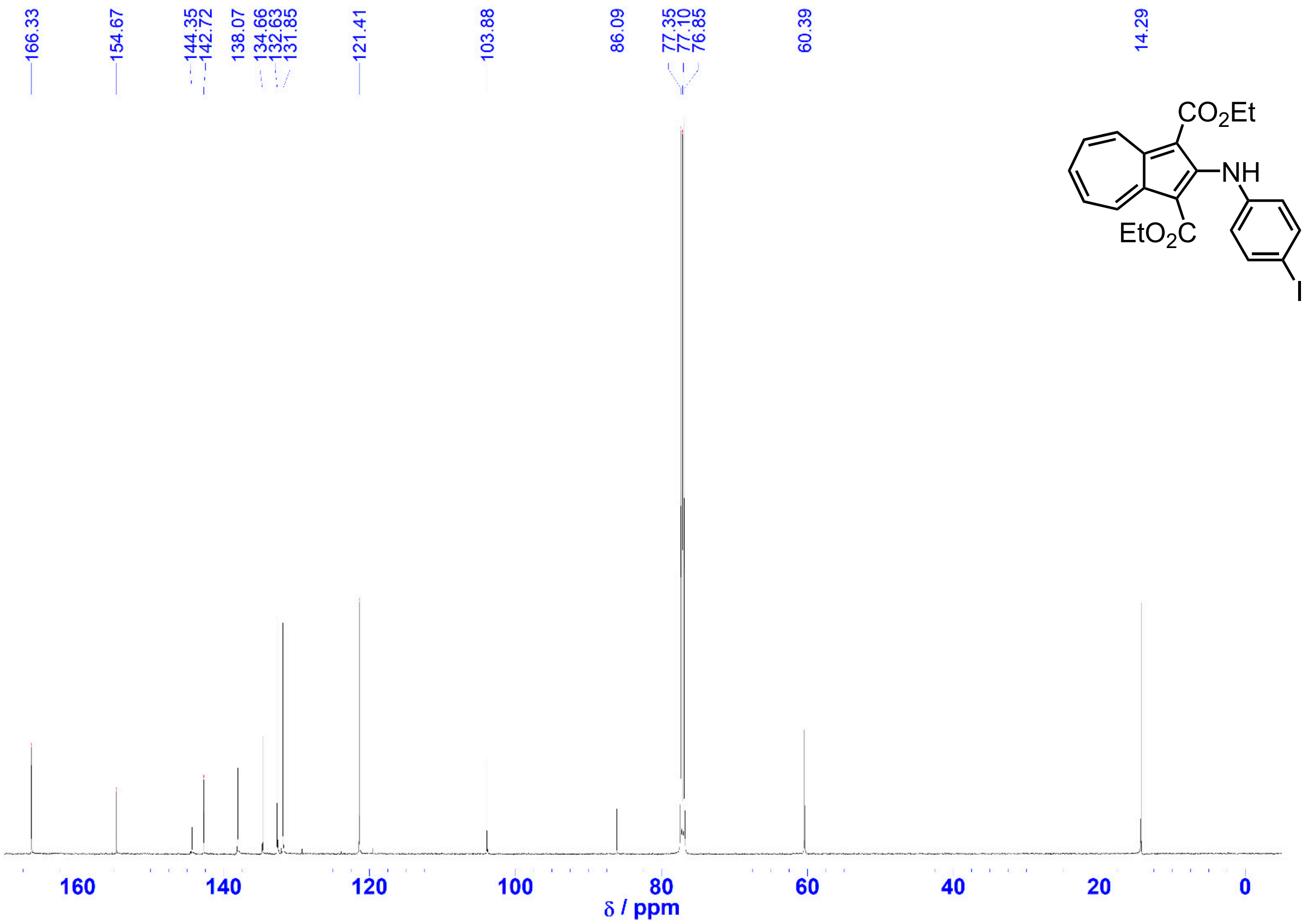

Figure S12. ^{13}C NMR spectrum of **2f** in $CDCl_3$ (125 MHz).

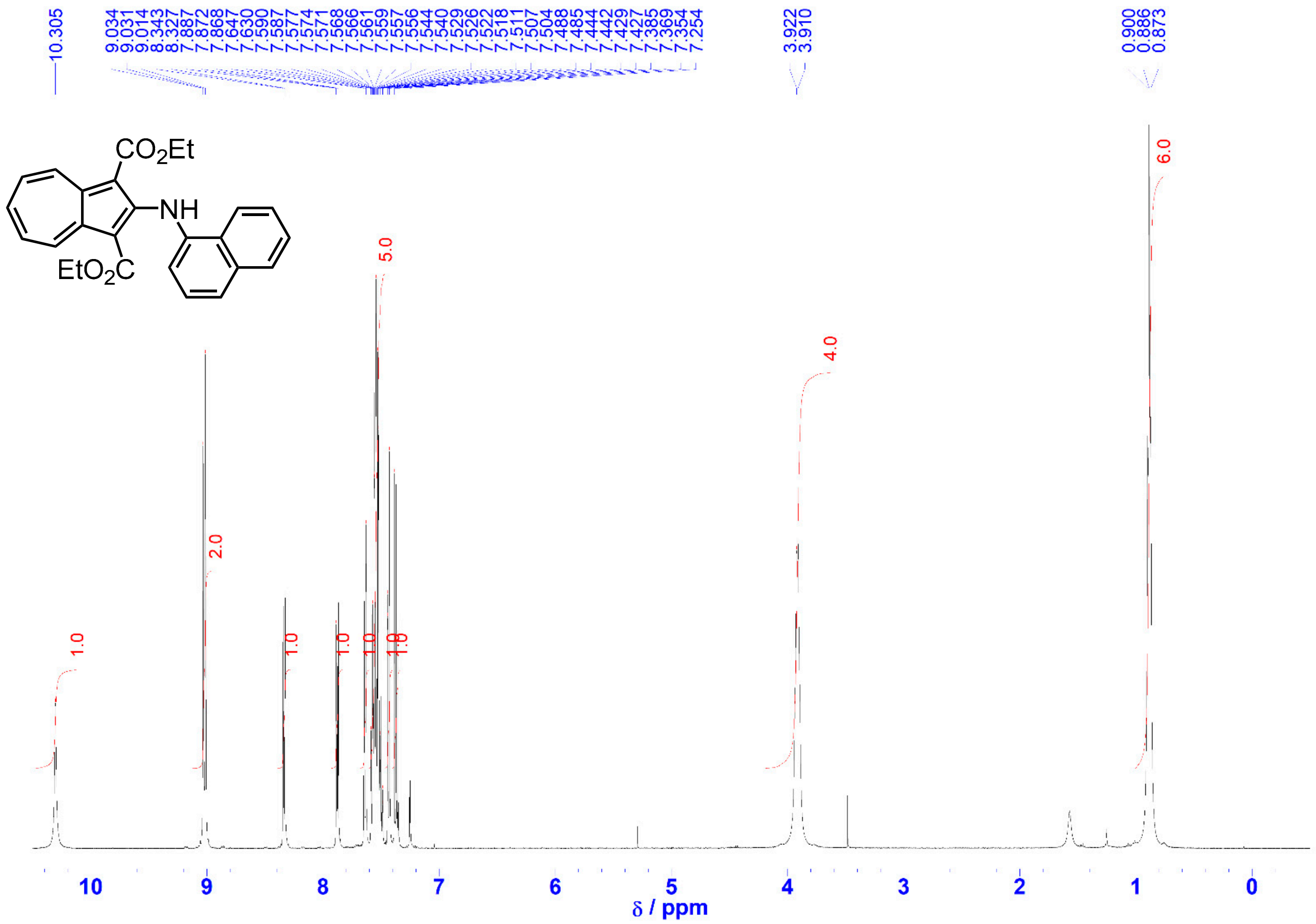

Figure S13. ^{1}H NMR spectrum of **2f** in $CDCl_3$ (500 MHz).

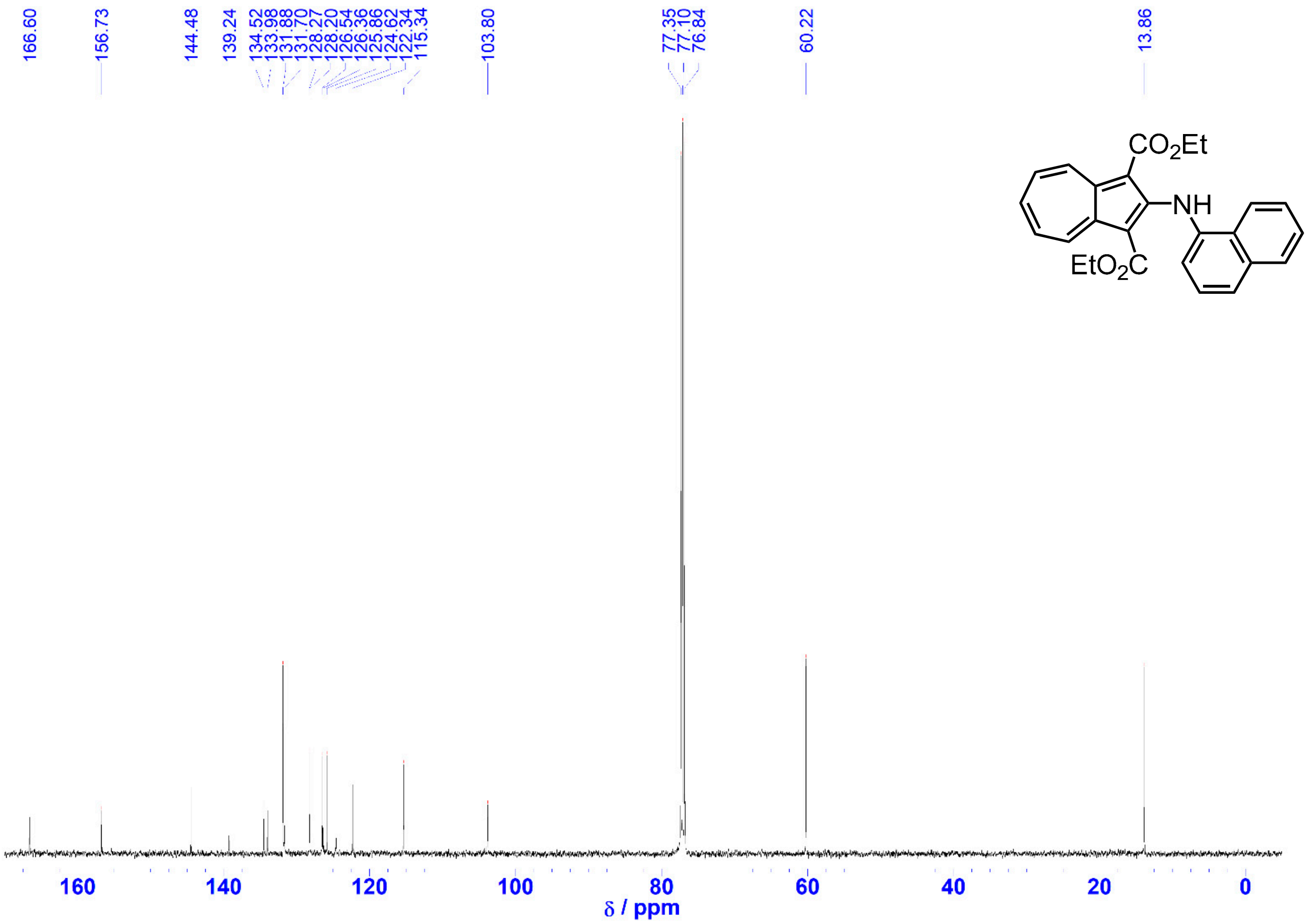

Figure S14. ^{13}C NMR spectrum of **2f** in $CDCl_3$ (125 MHz).

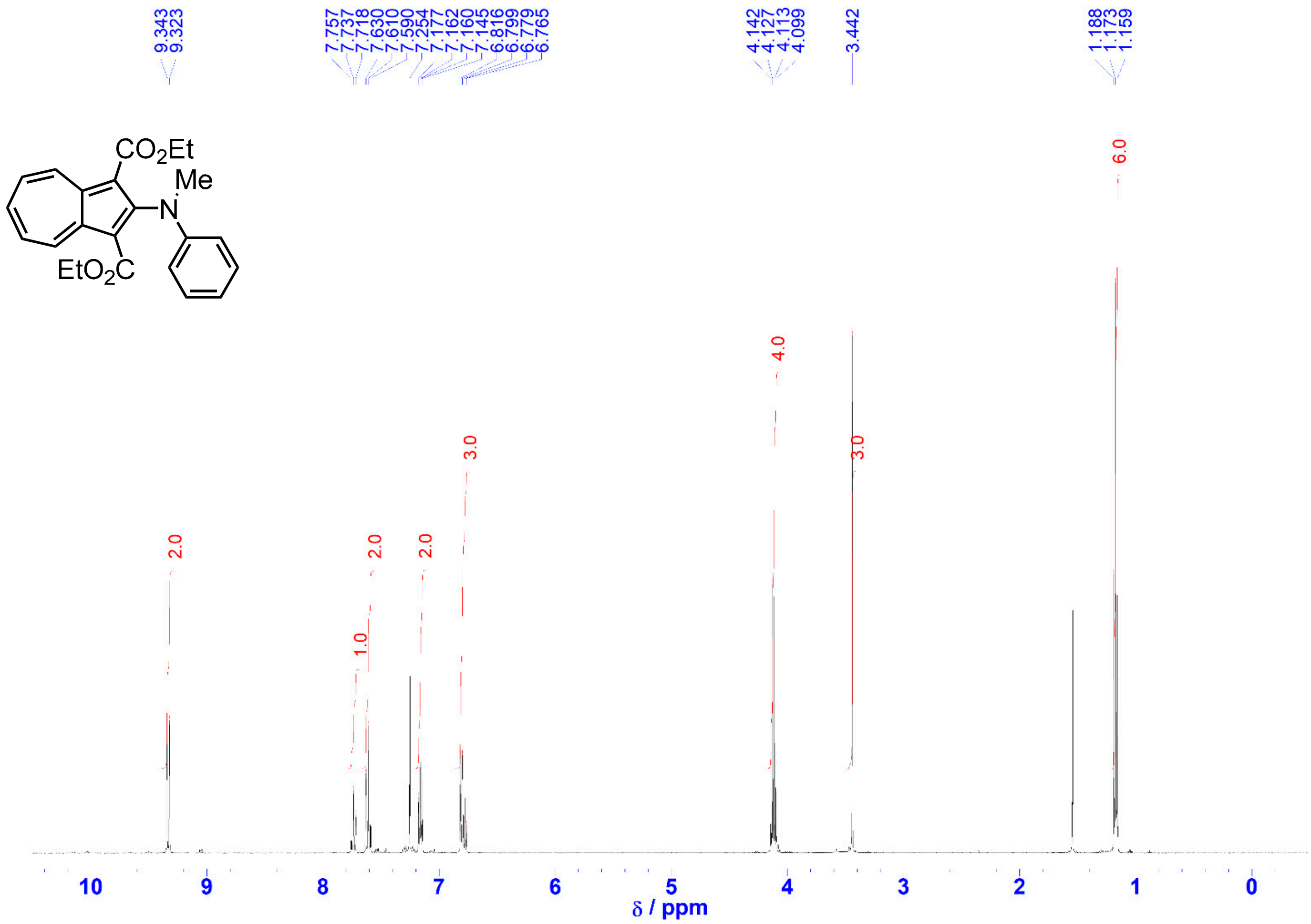

Figure S15. ^{1}H NMR spectrum of **2g** in $CDCl_3$ (500 MHz).

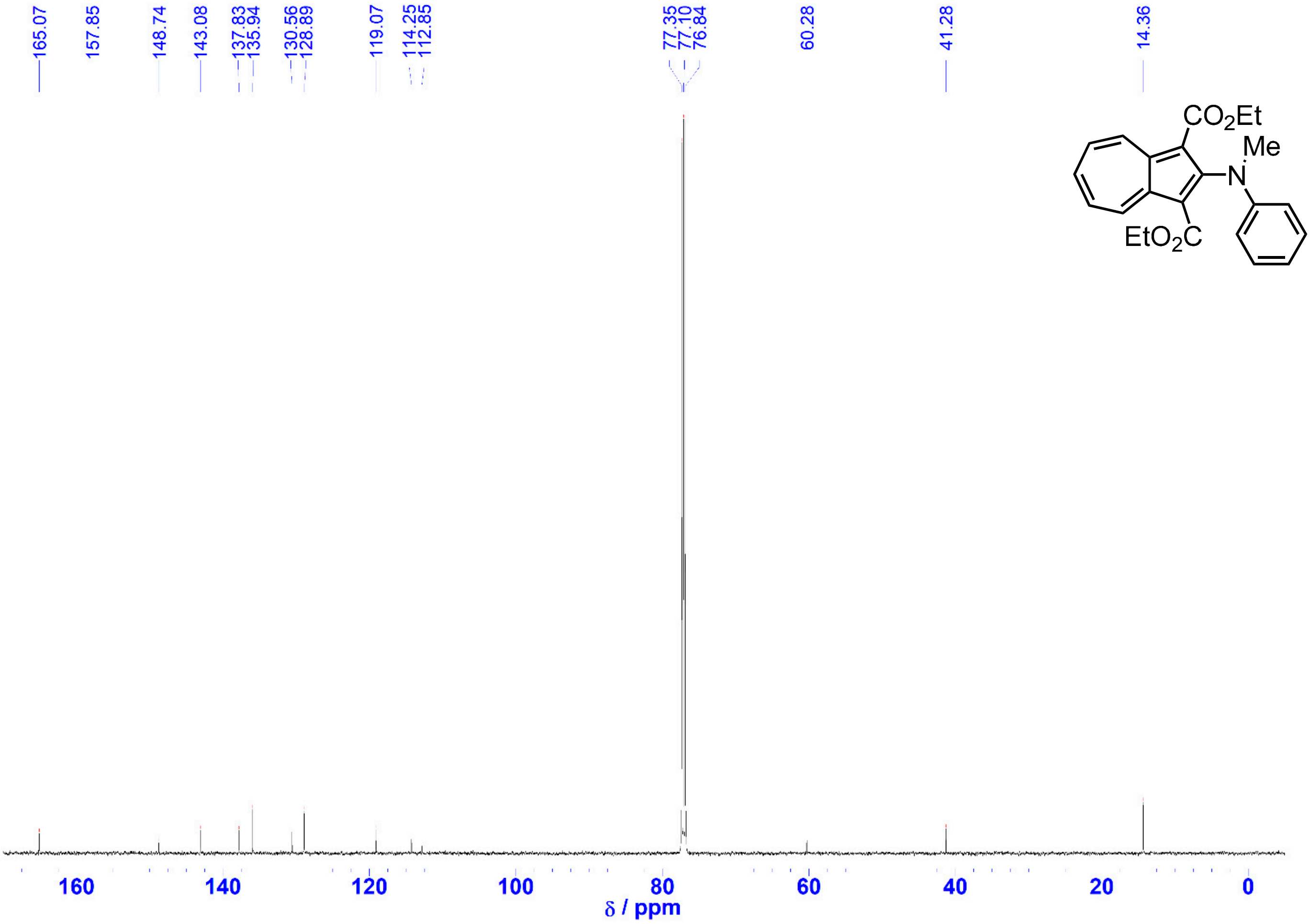

Figure S16. ^{13}C NMR spectrum of **2g** in $CDCl_3$ (125 MHz).

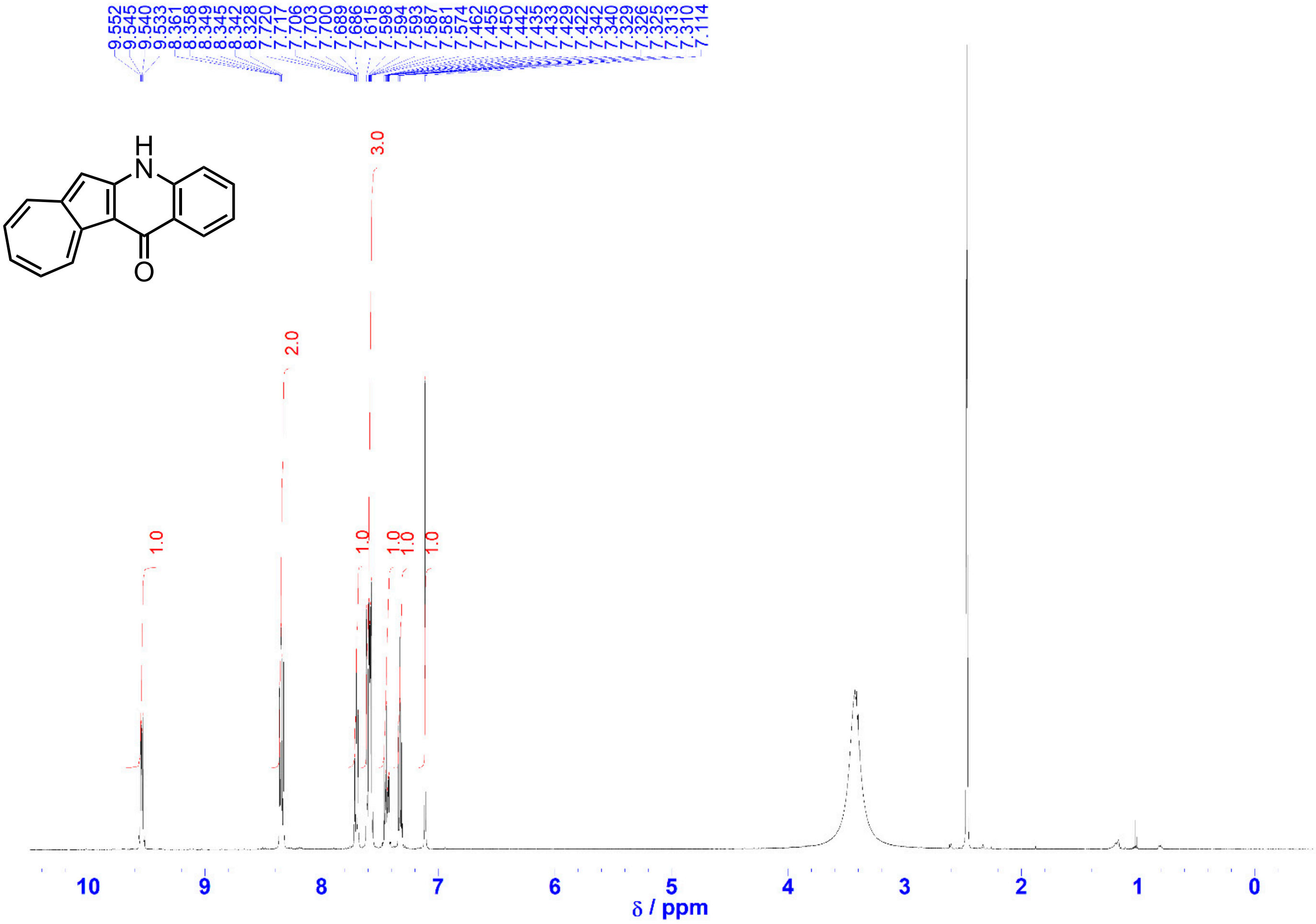

Figure S17. ^{1}H NMR spectrum of **3a** in DMSO-d_6 (500 MHz).

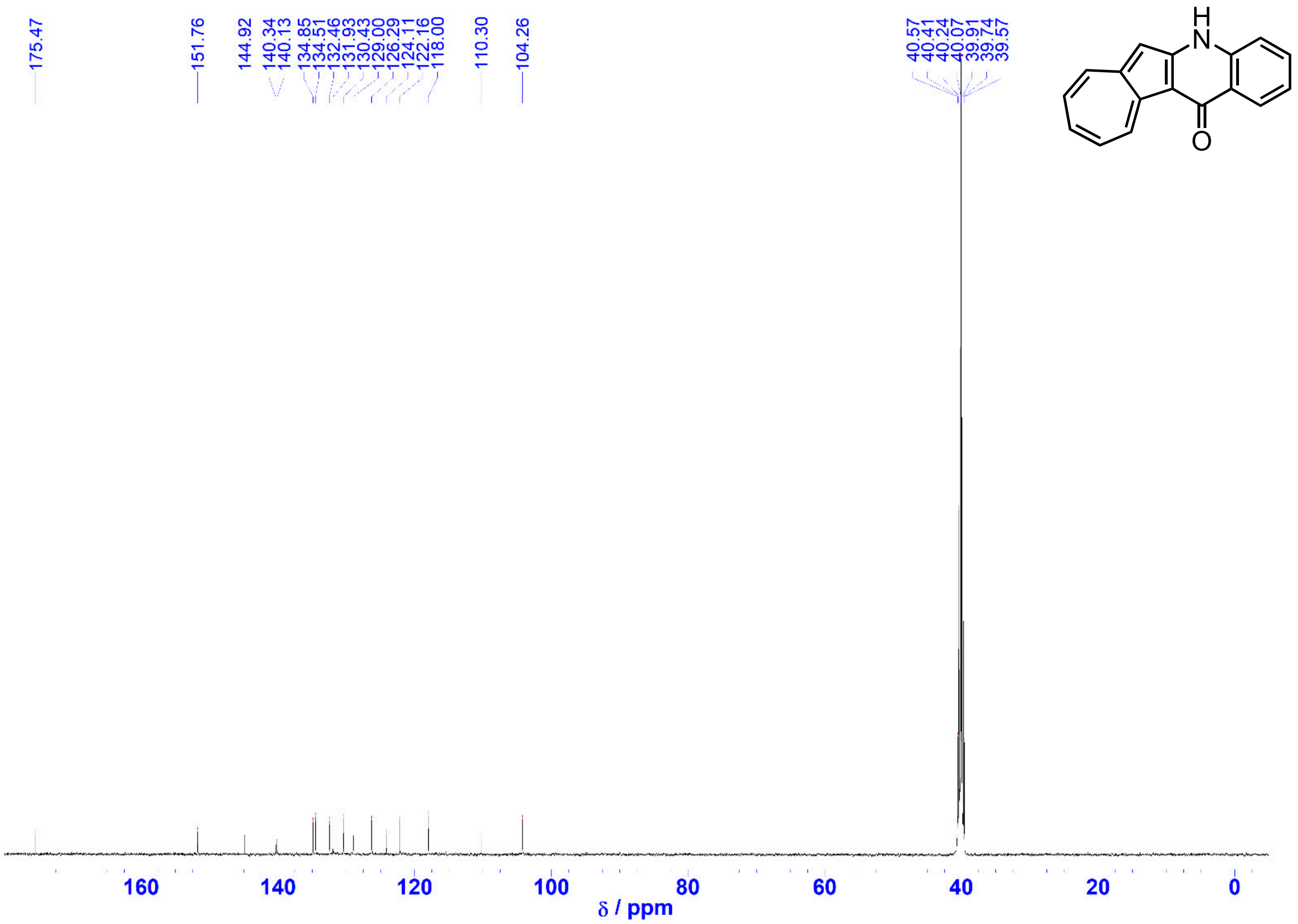

Figure S18. ^{13}C NMR spectrum of **3a** in DMSO-d_6 (125 MHz).

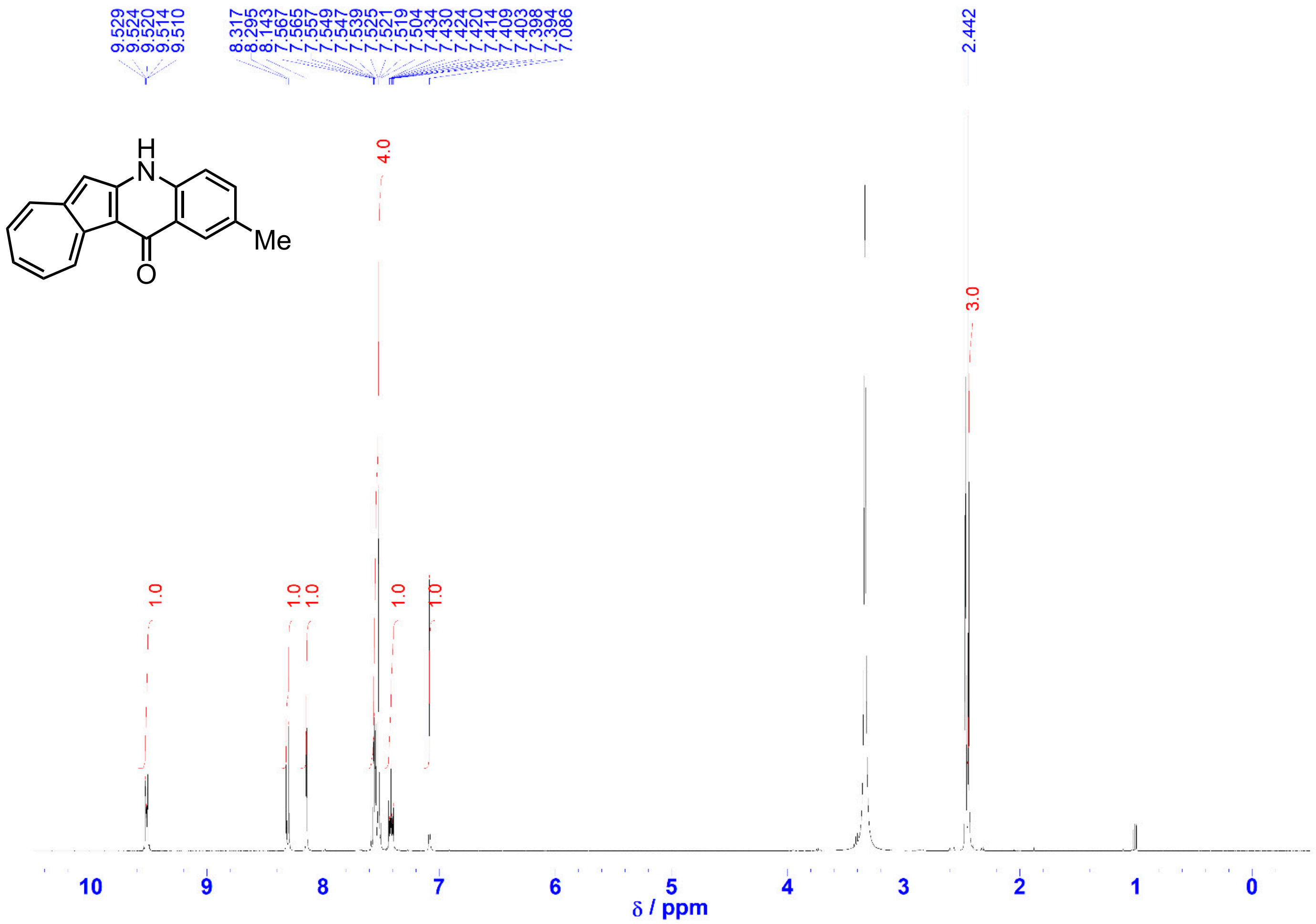

Figure S19. ^{1}H NMR spectrum of **3b** in DMSO-d_6 (500 MHz).

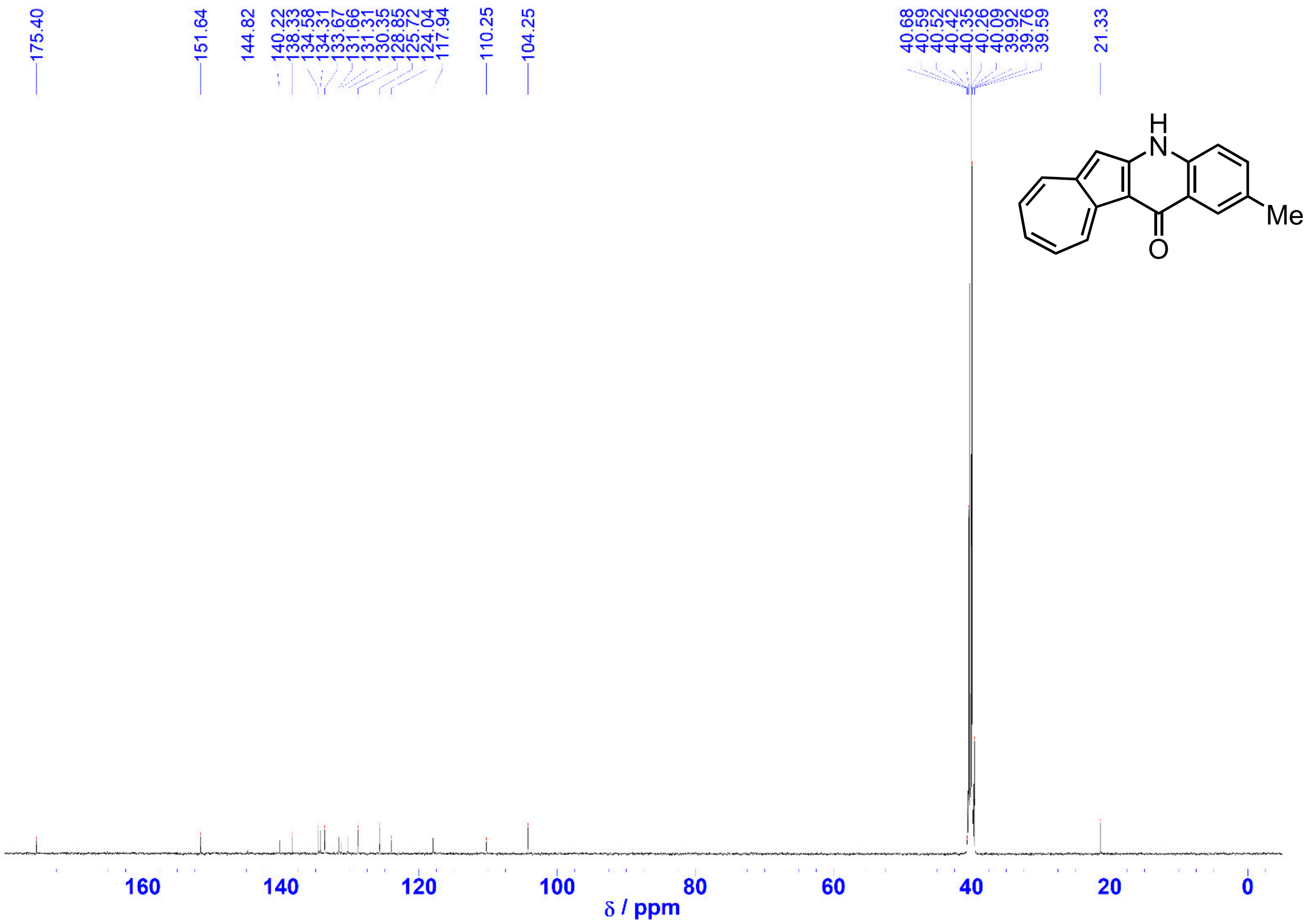

Figure S20. ^{13}C NMR spectrum of **3b** in DMSO-d_6 (125 MHz).

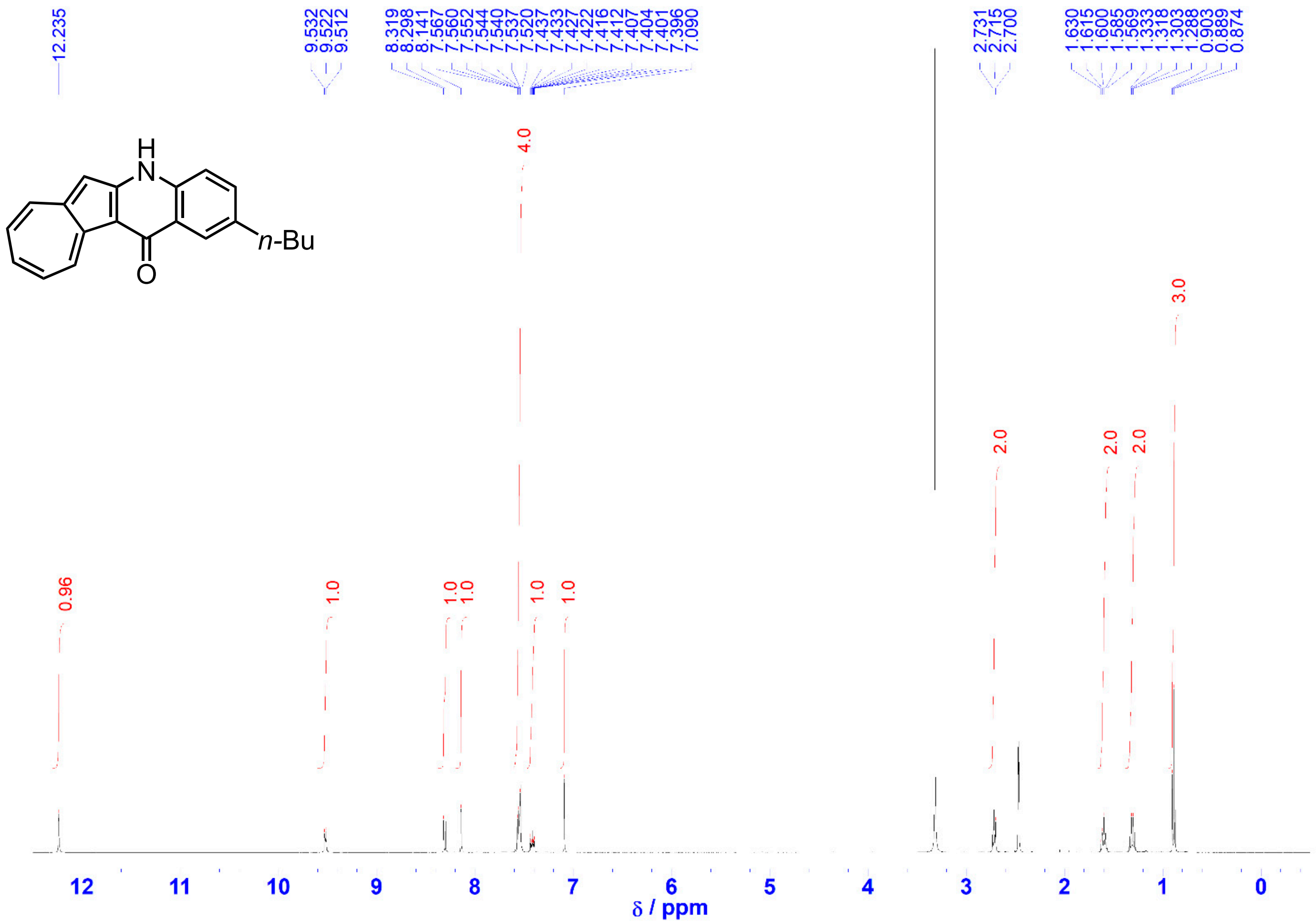

Figure S21. ^{1}H NMR spectrum of **3c** in DMSO-d_6 (500 MHz).

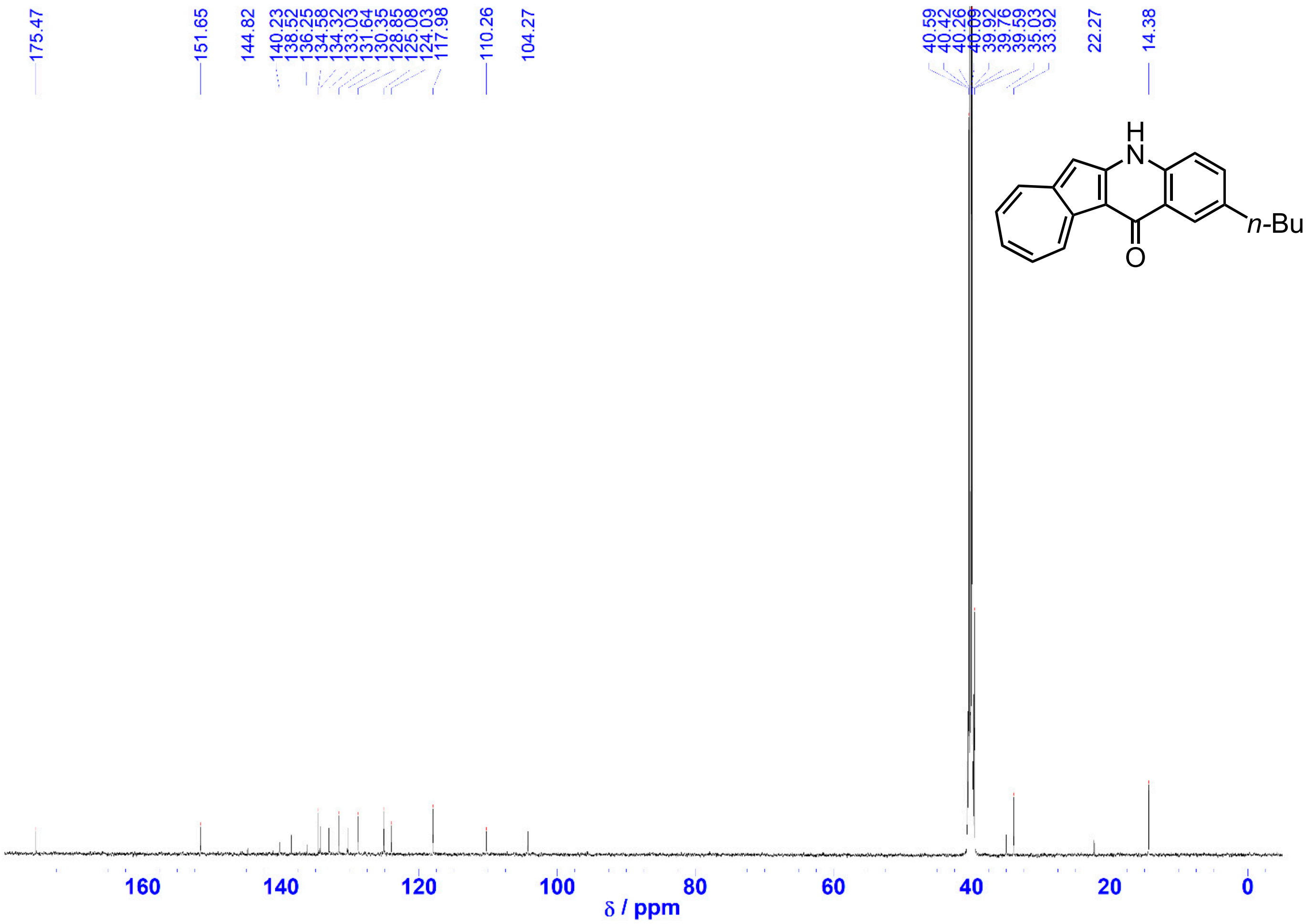

Figure S22. ^{13}C NMR spectrum of **3c** in DMSO-d_6 (125 MHz).

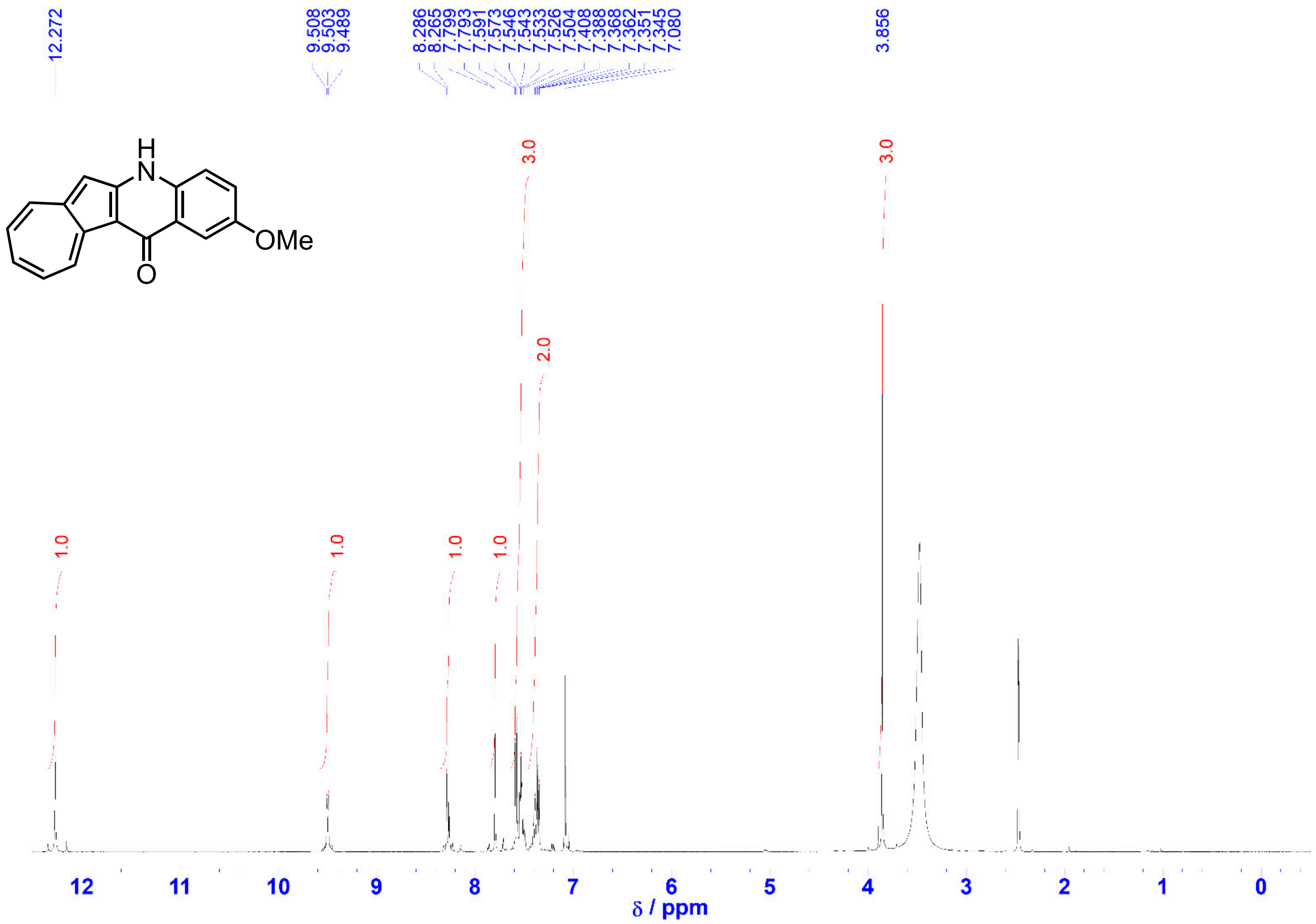

Figure S23. ^{1}H NMR spectrum of **3d** in DMSO-d_6 (500 MHz).

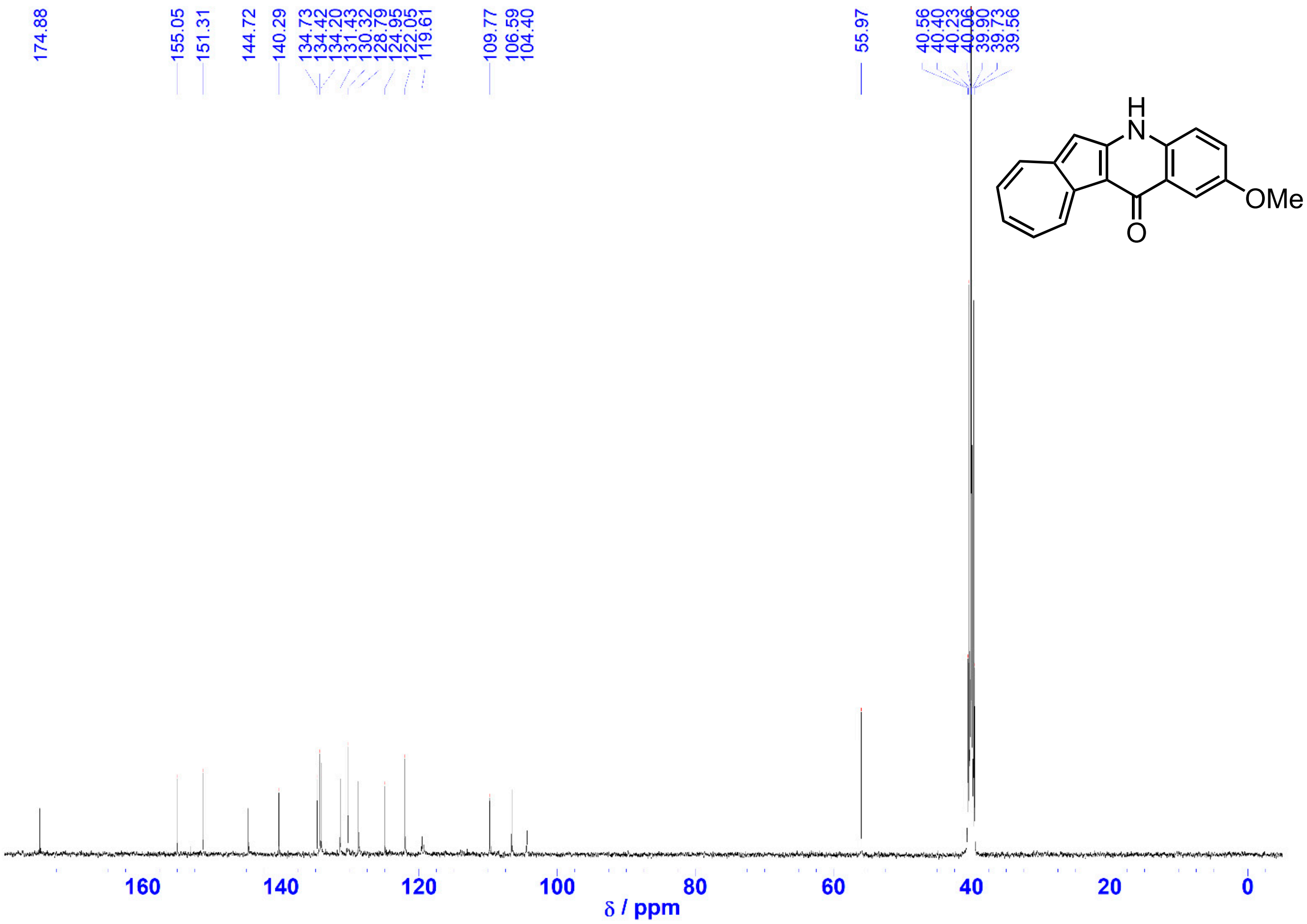

Figure S24. ^{13}C NMR spectrum of **3d** in DMSO-d_6 (125 MHz).

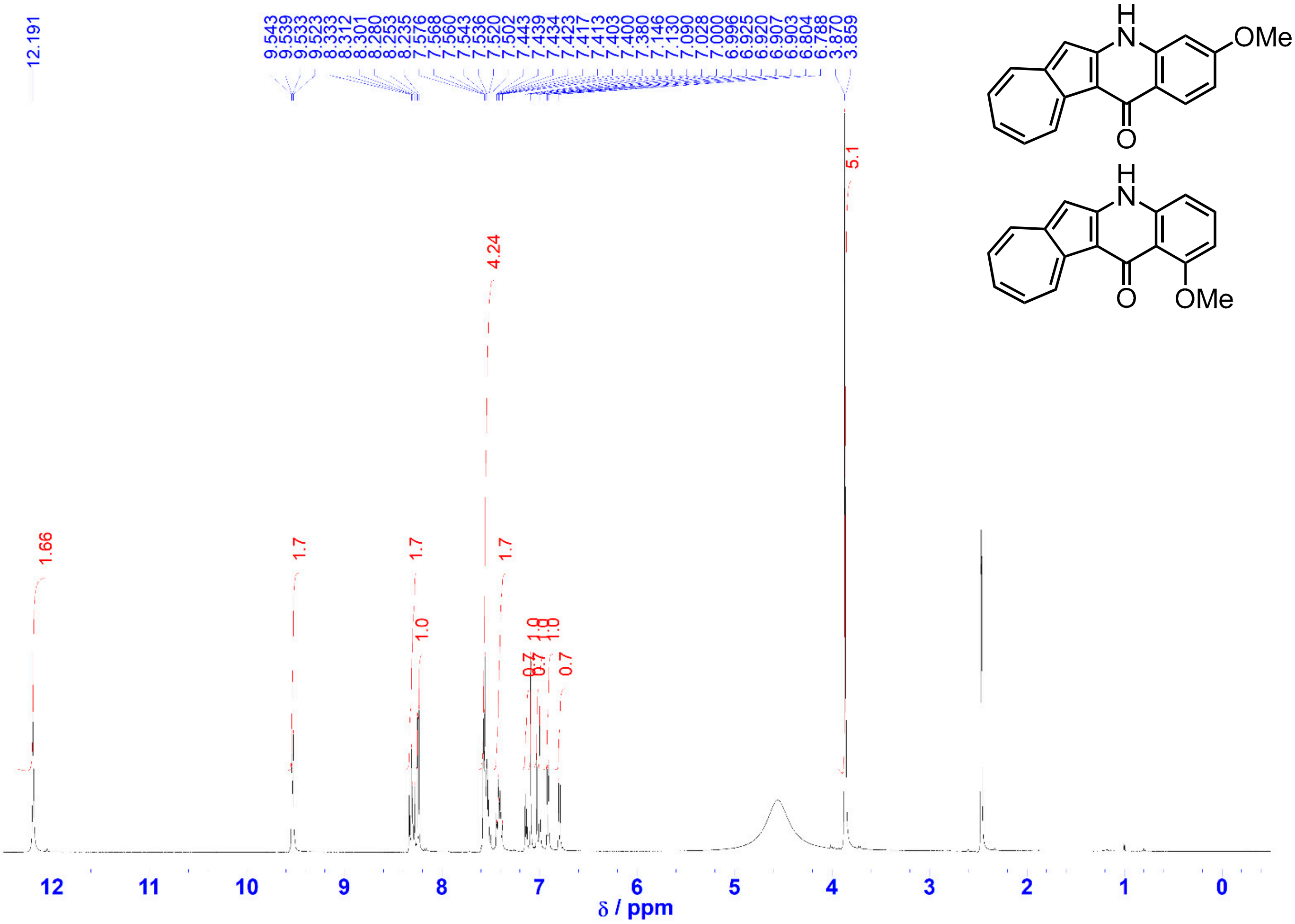

Figure S25. ^{1}H NMR spectrum of **3e** and **3e′** in DMSO-d_6 (500 MHz).

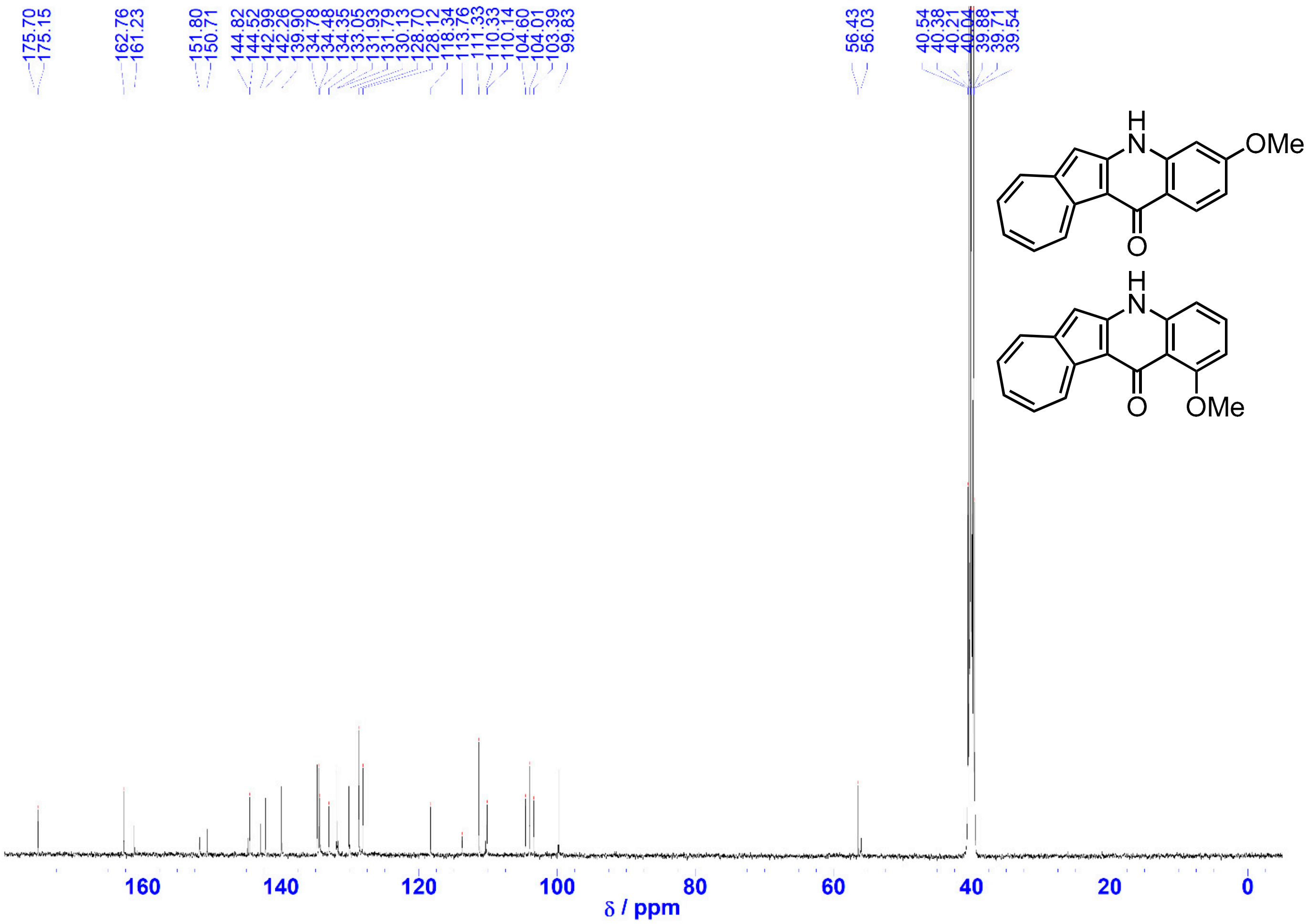

Figure S26. ^{13}C NMR spectrum of **3e** and **3e′** in DMSO-d_6 (125 MHz).

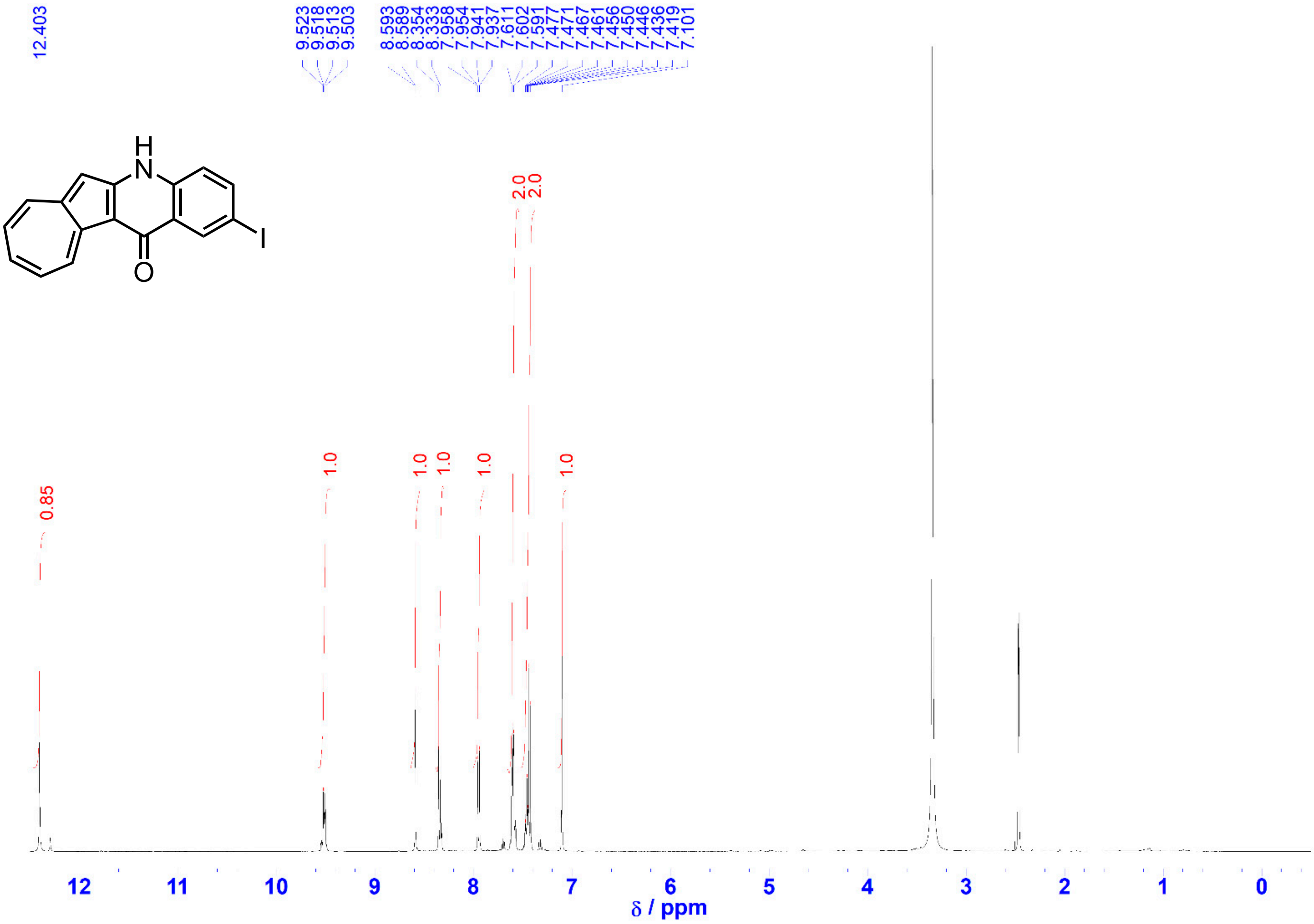

Figure S27. ^{1}H NMR spectrum of **3f** in DMSO-d_6 (500 MHz).

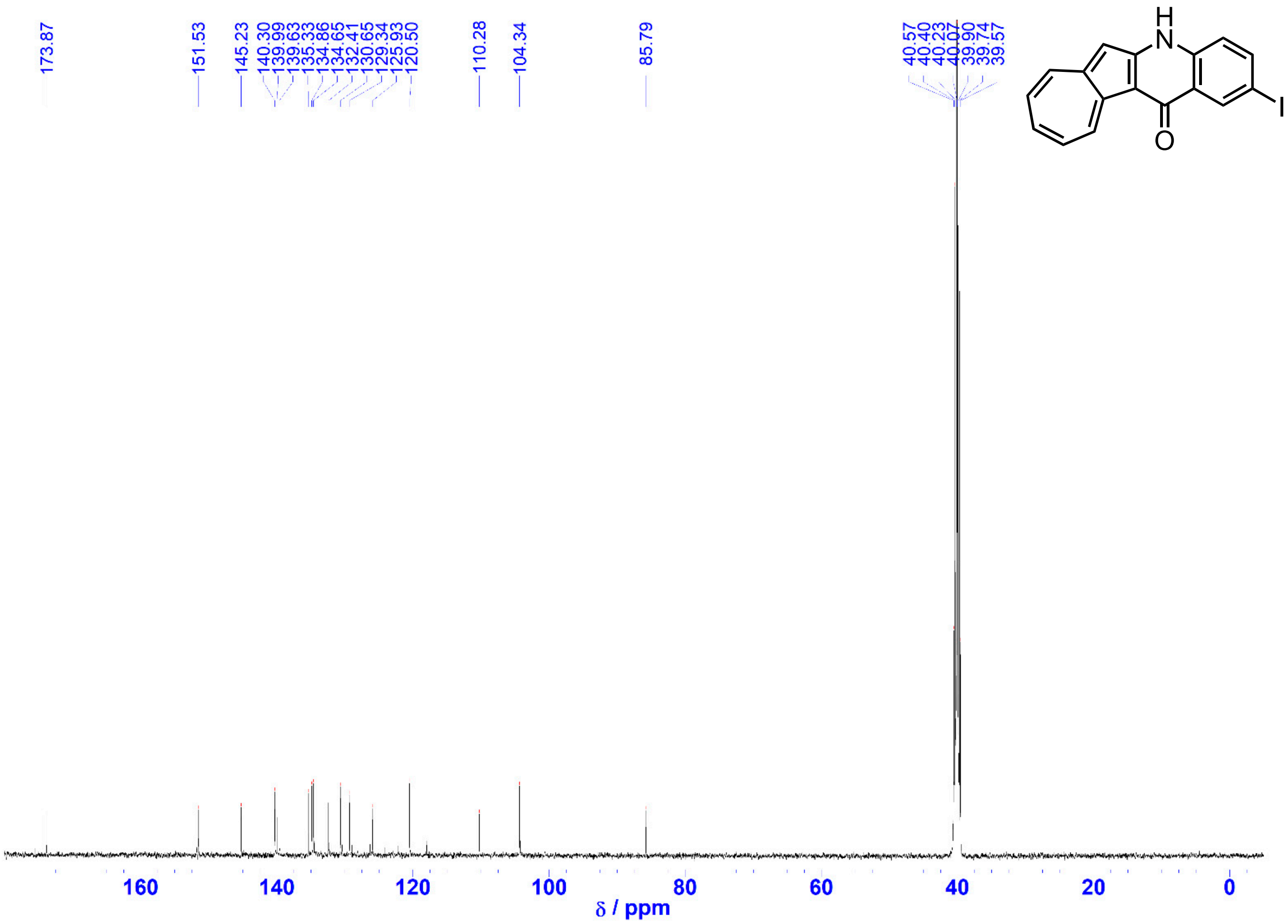

Figure S28. ^{13}C NMR spectrum of **3f** in DMSO-d_6 (125 MHz).

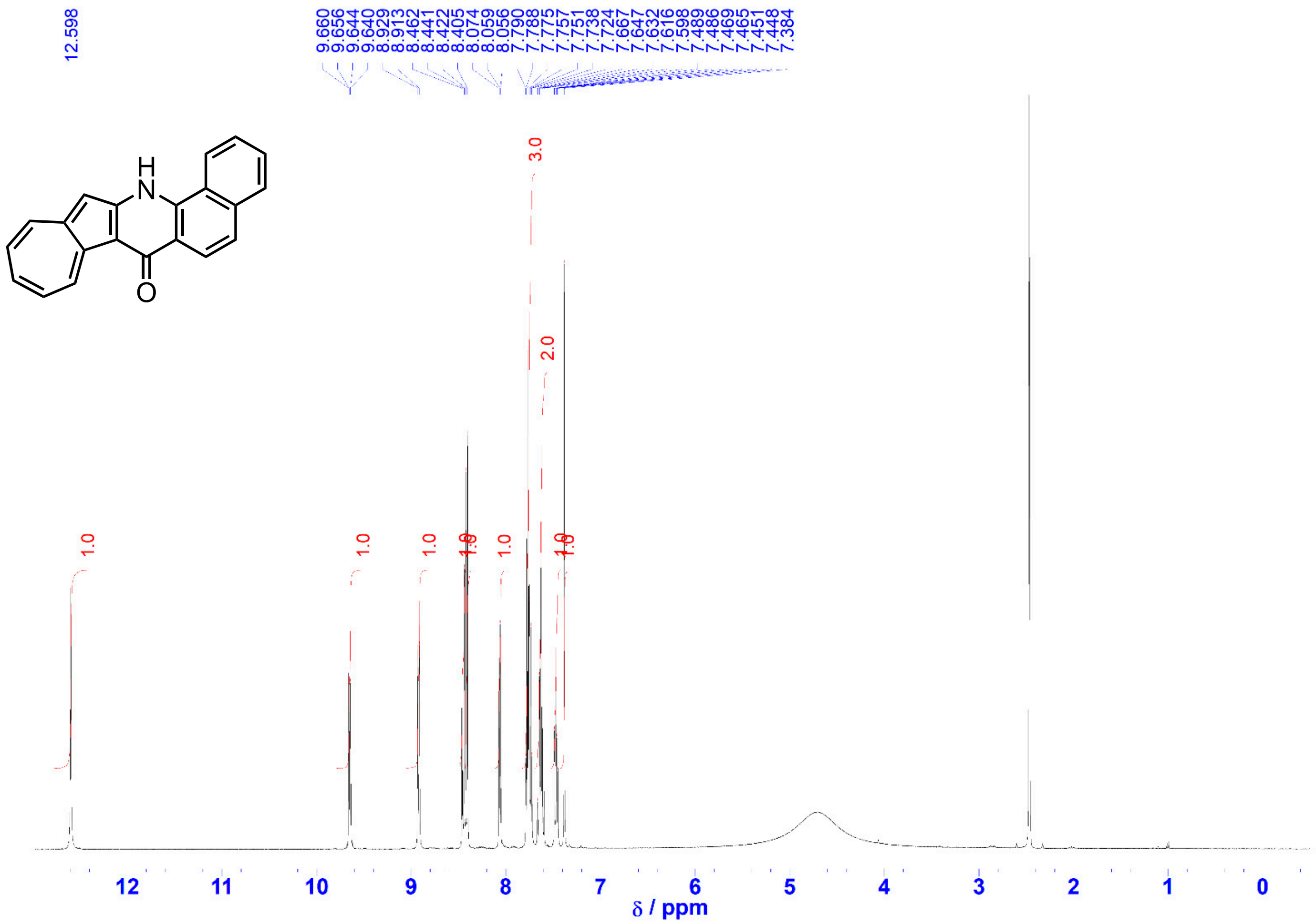

Figure S29. ¹H NMR spectrum of **3g** in DMSO-d_6 (500 MHz).

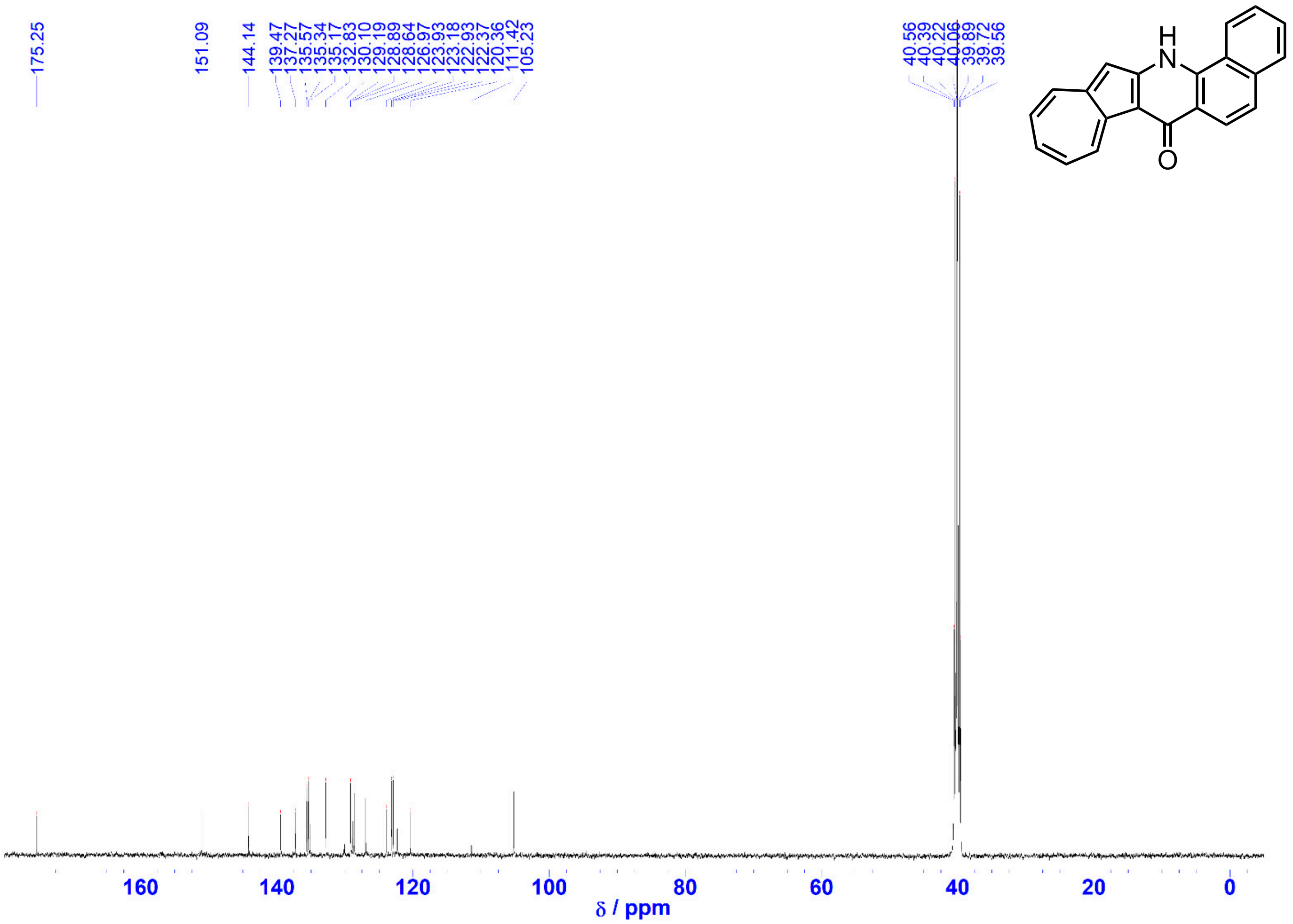

Figure S30. ^{13}C NMR spectrum of **3g** in DMSO-d_6 (125 MHz).

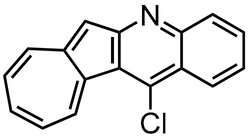

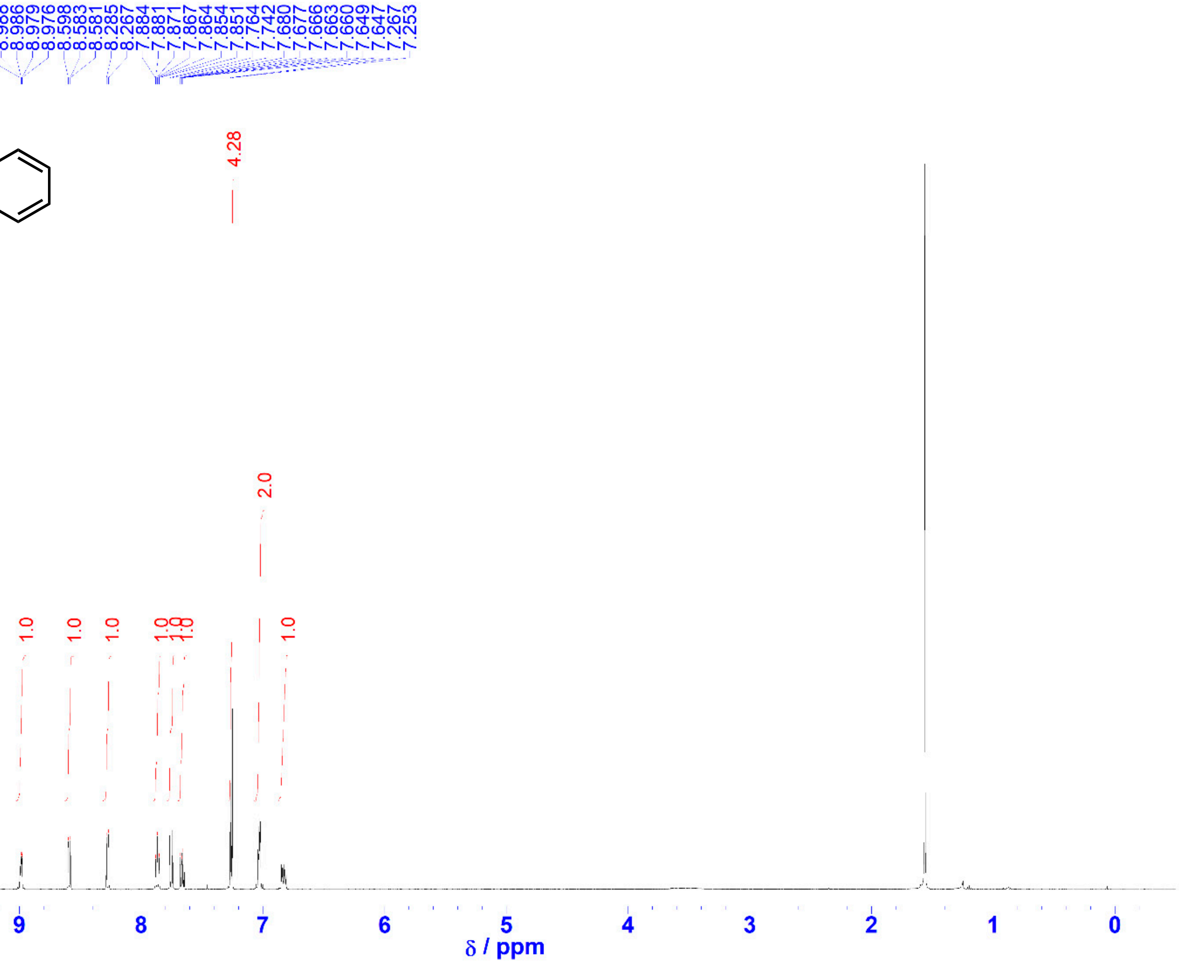

Figure S31. ^{1}H NMR spectrum of **4a** in $CDCl_3$ (500 MHz).

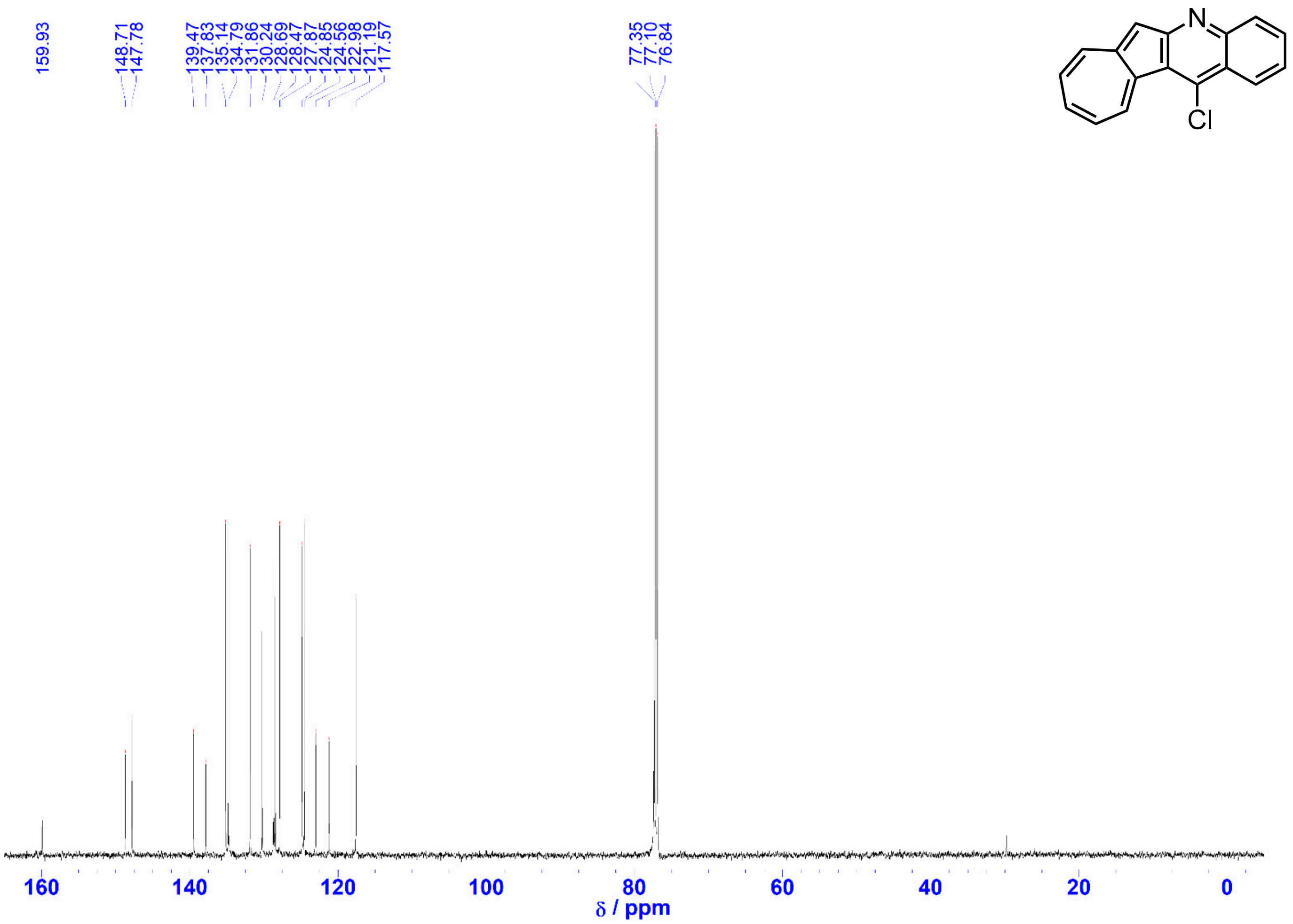

Figure S32. ^{13}C NMR spectrum of **4a** in $CDCl_3$ (125 MHz).

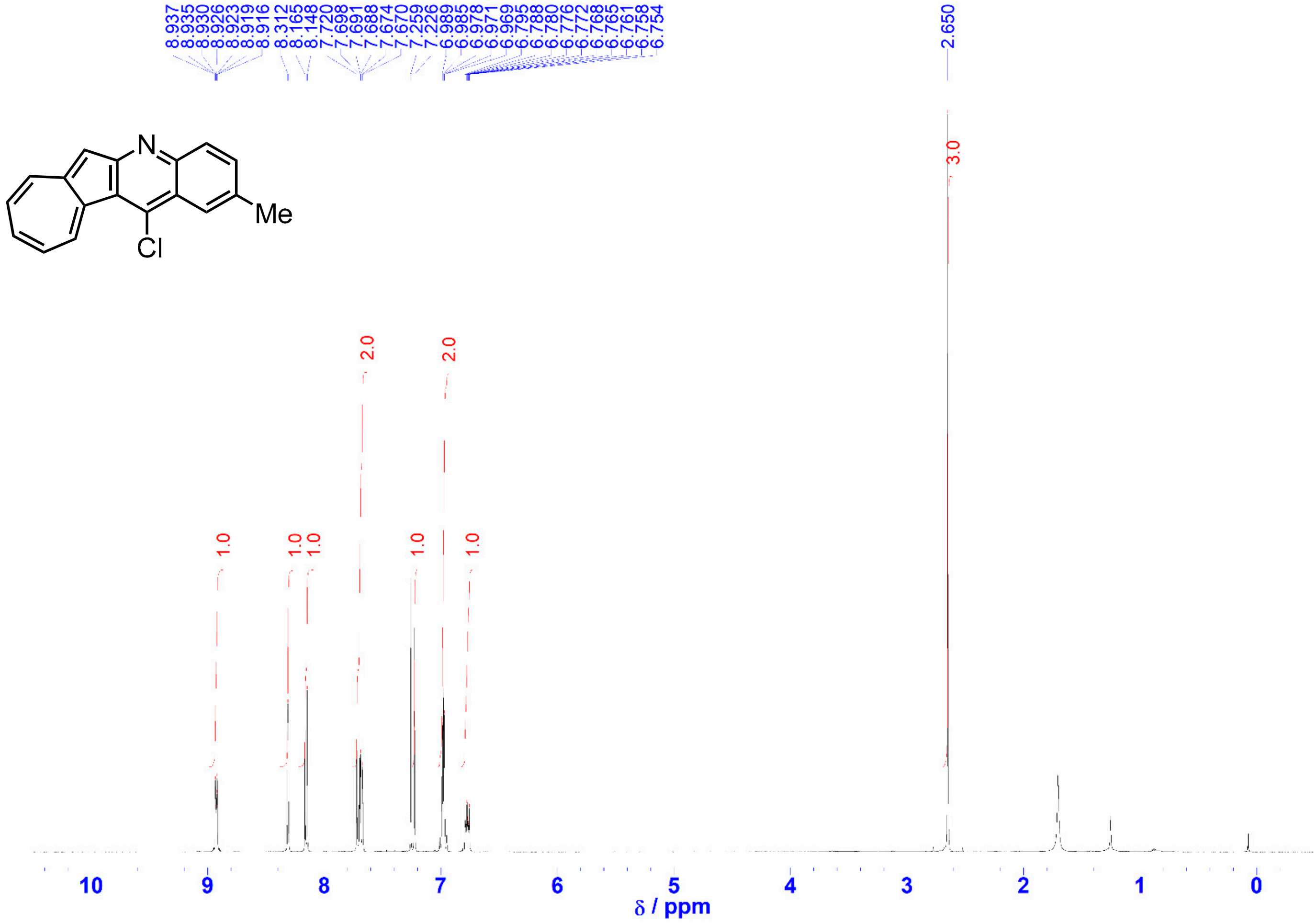

Figure S33. ^1H NMR spectrum of **4b** in $CDCl_3$ (500 MHz).

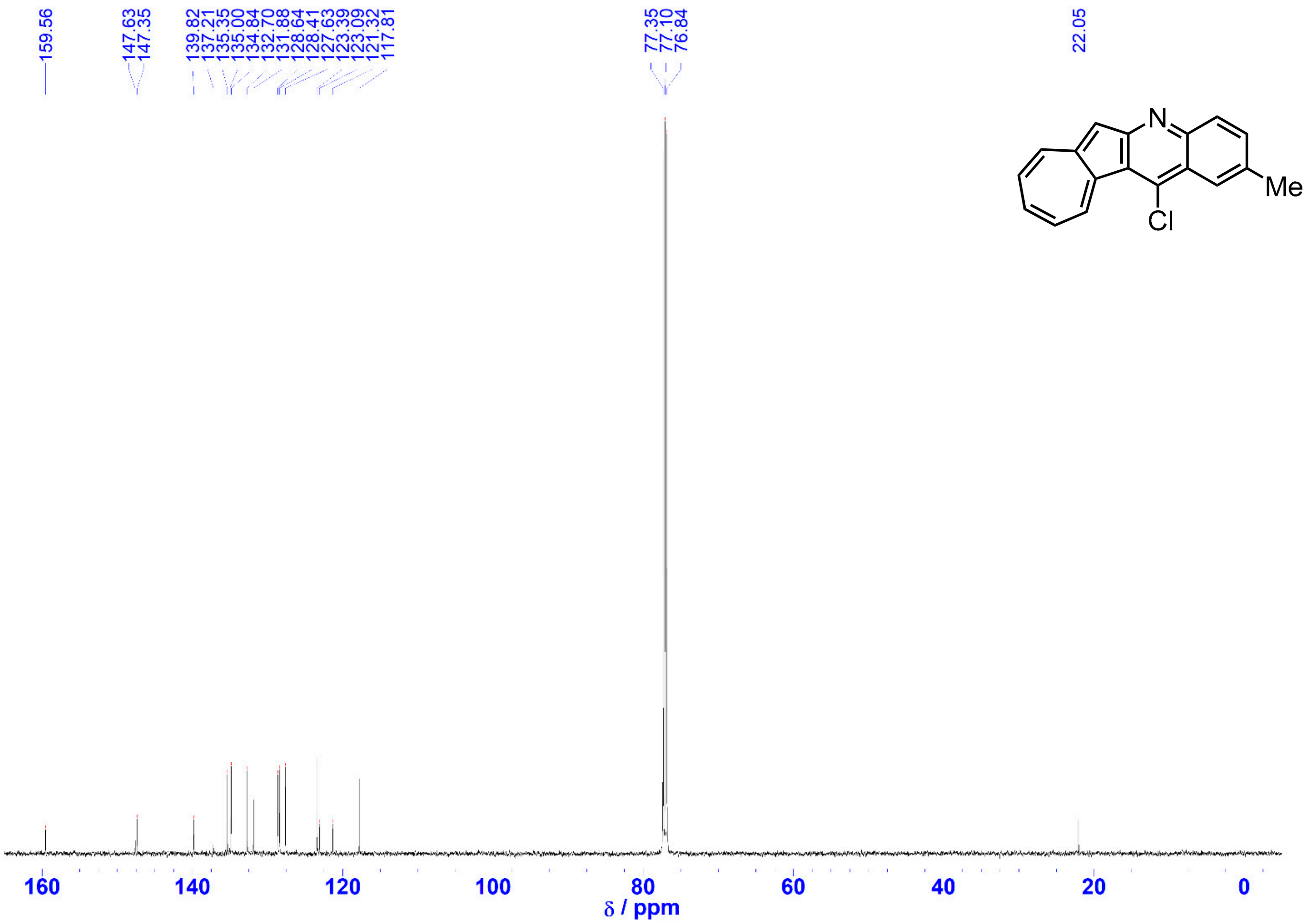

Figure S34. ^{13}C NMR spectrum of **4b** in $CDCl_3$ (125 MHz).

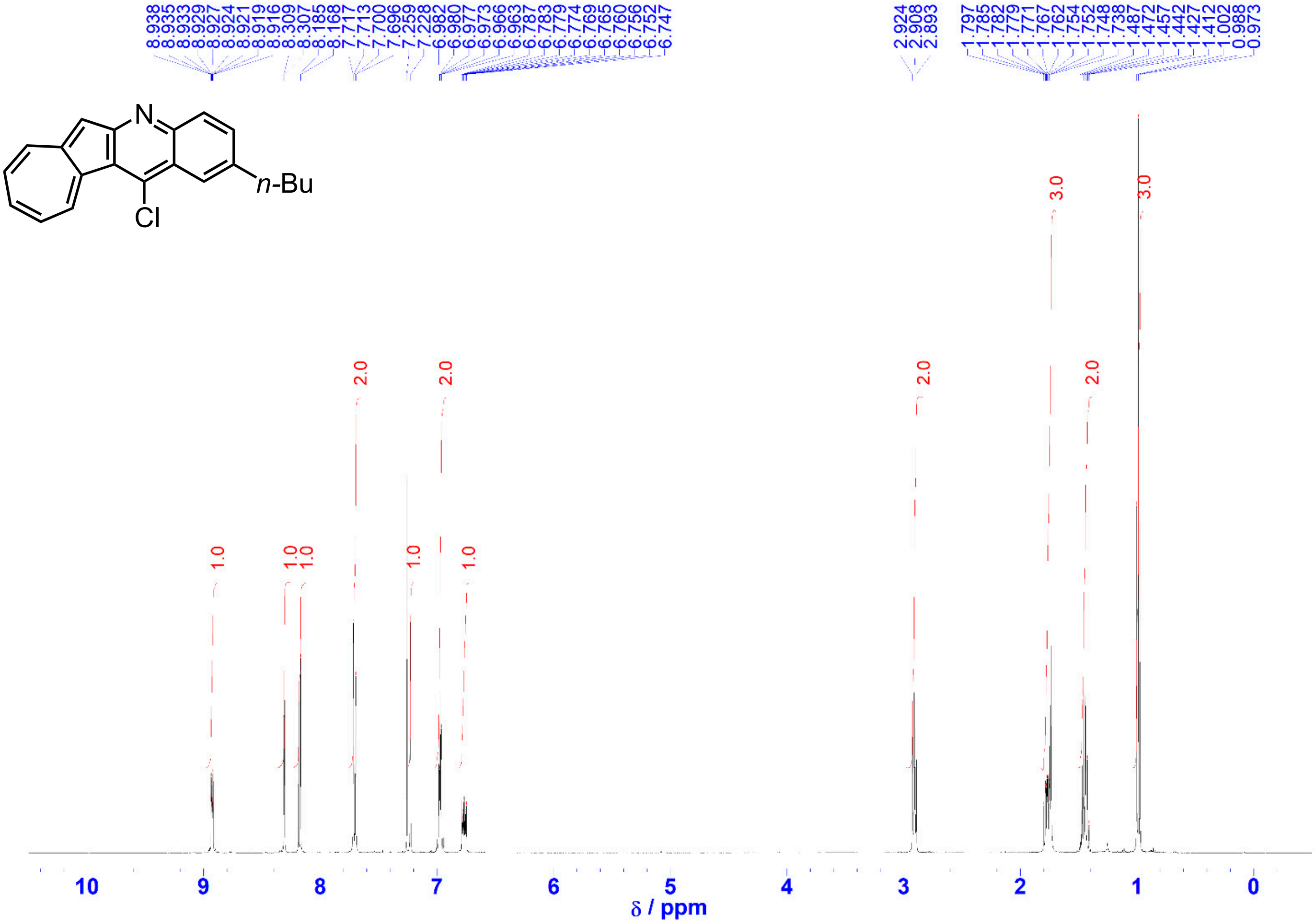

Figure S35. ^{1}H NMR spectrum of **4c** in $CDCl_3$ (500 MHz).

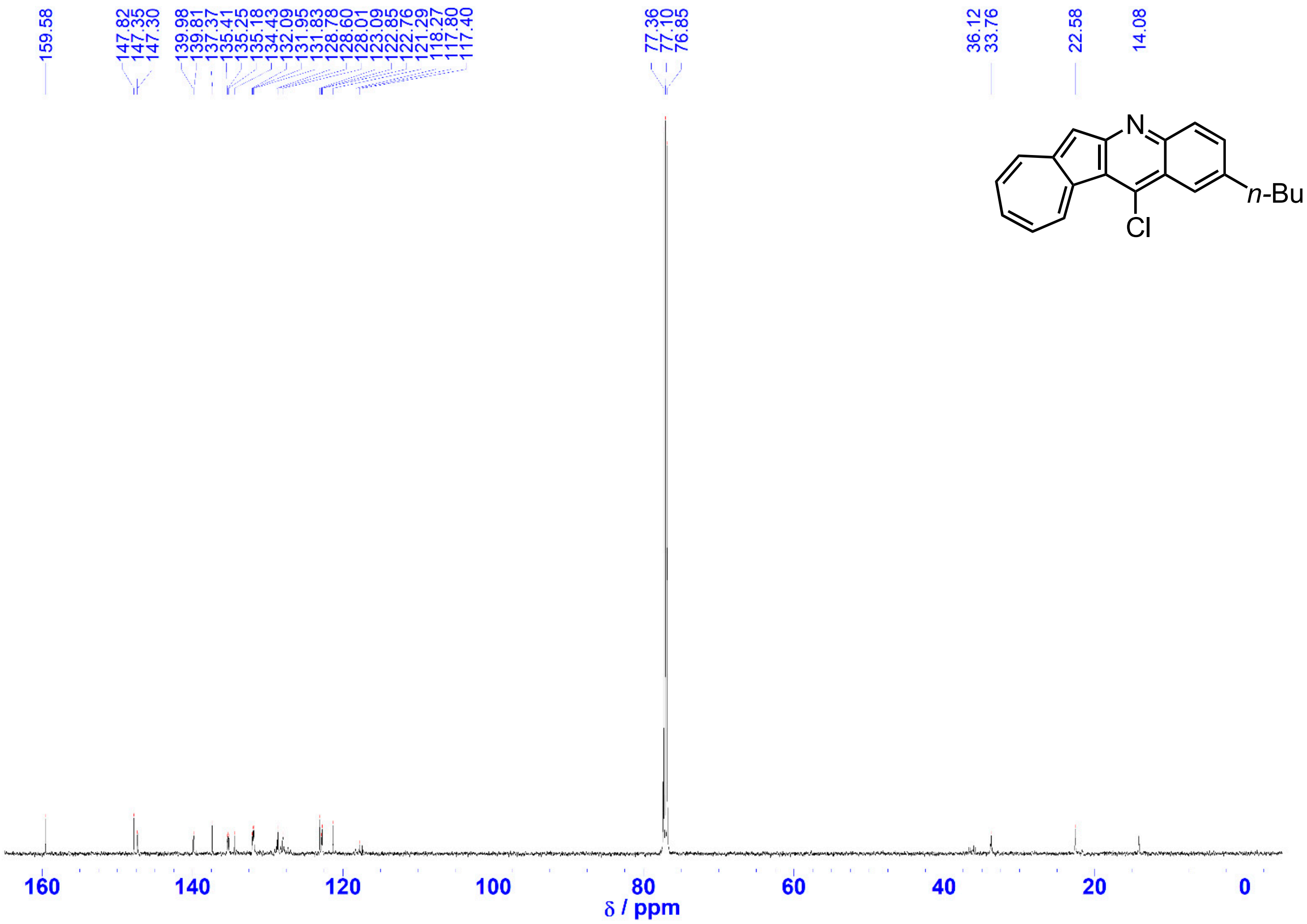

Figure S36. ^{13}C NMR spectrum of **4c** in $CDCl_3$ (125 MHz).

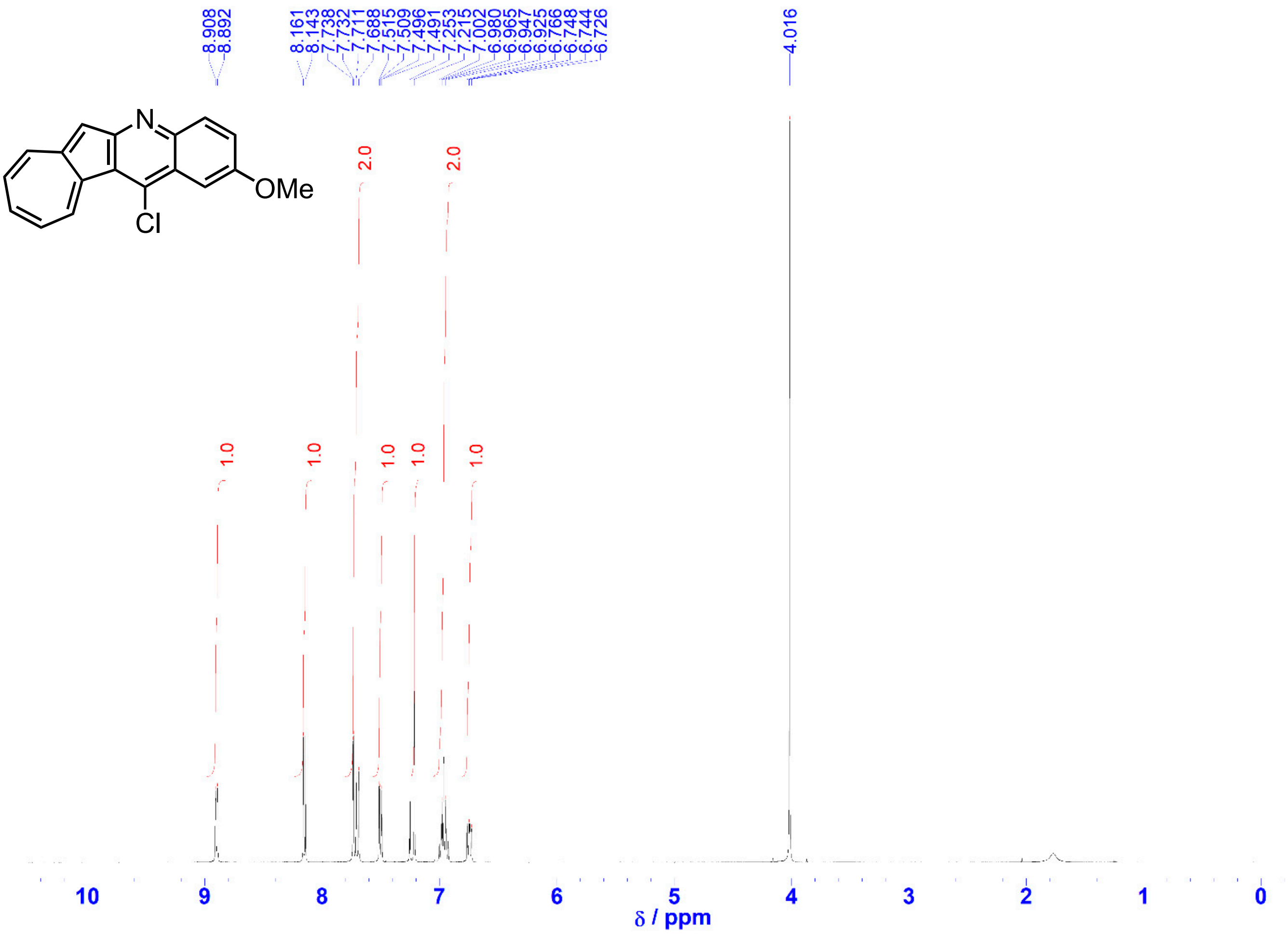

Figure S37. ^{1}H NMR spectrum of **4d** in $CDCl_3$ (500 MHz).

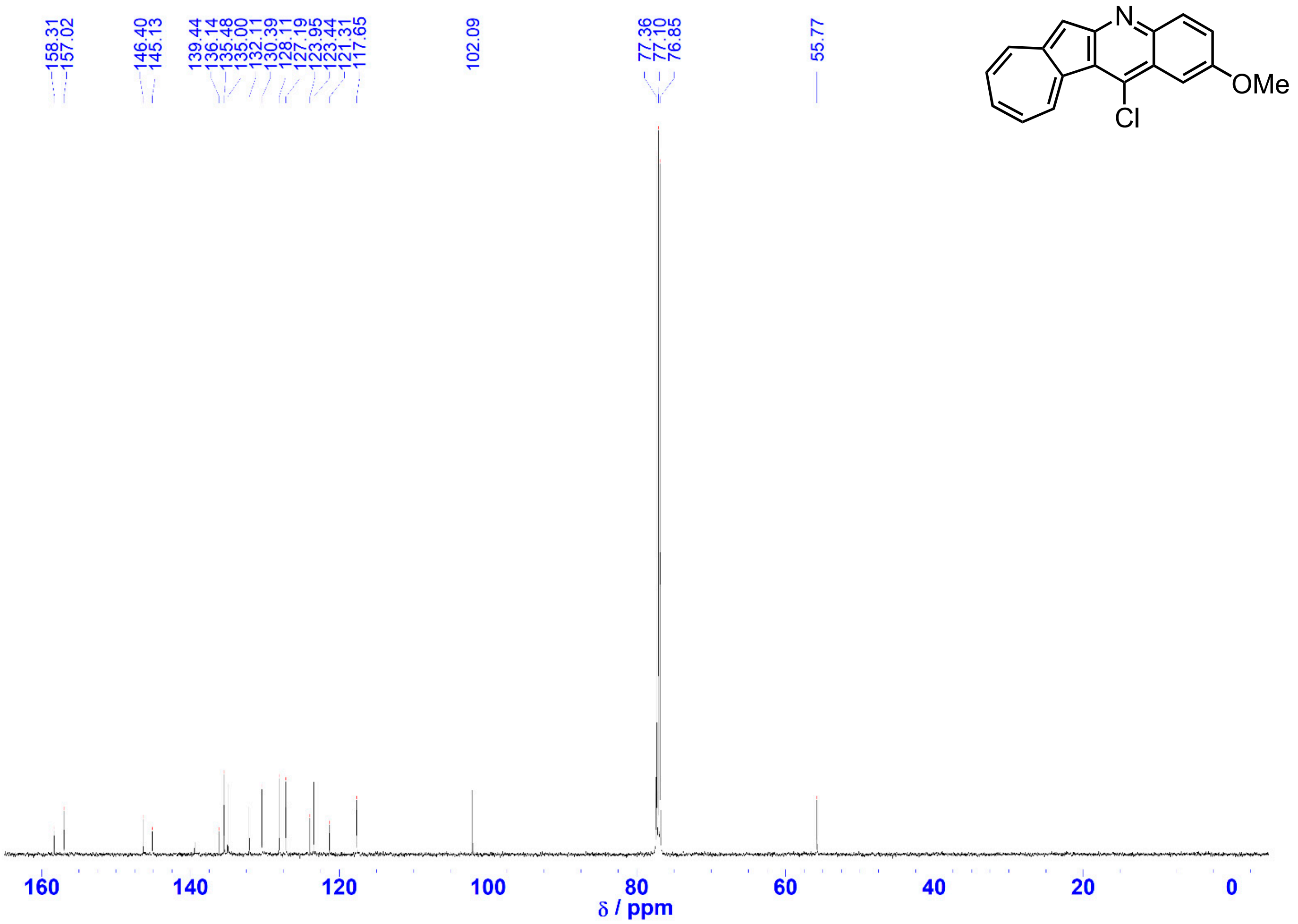

Figure S38. ^{13}C NMR spectrum of **4d** in $CDCl_3$ (125 MHz).

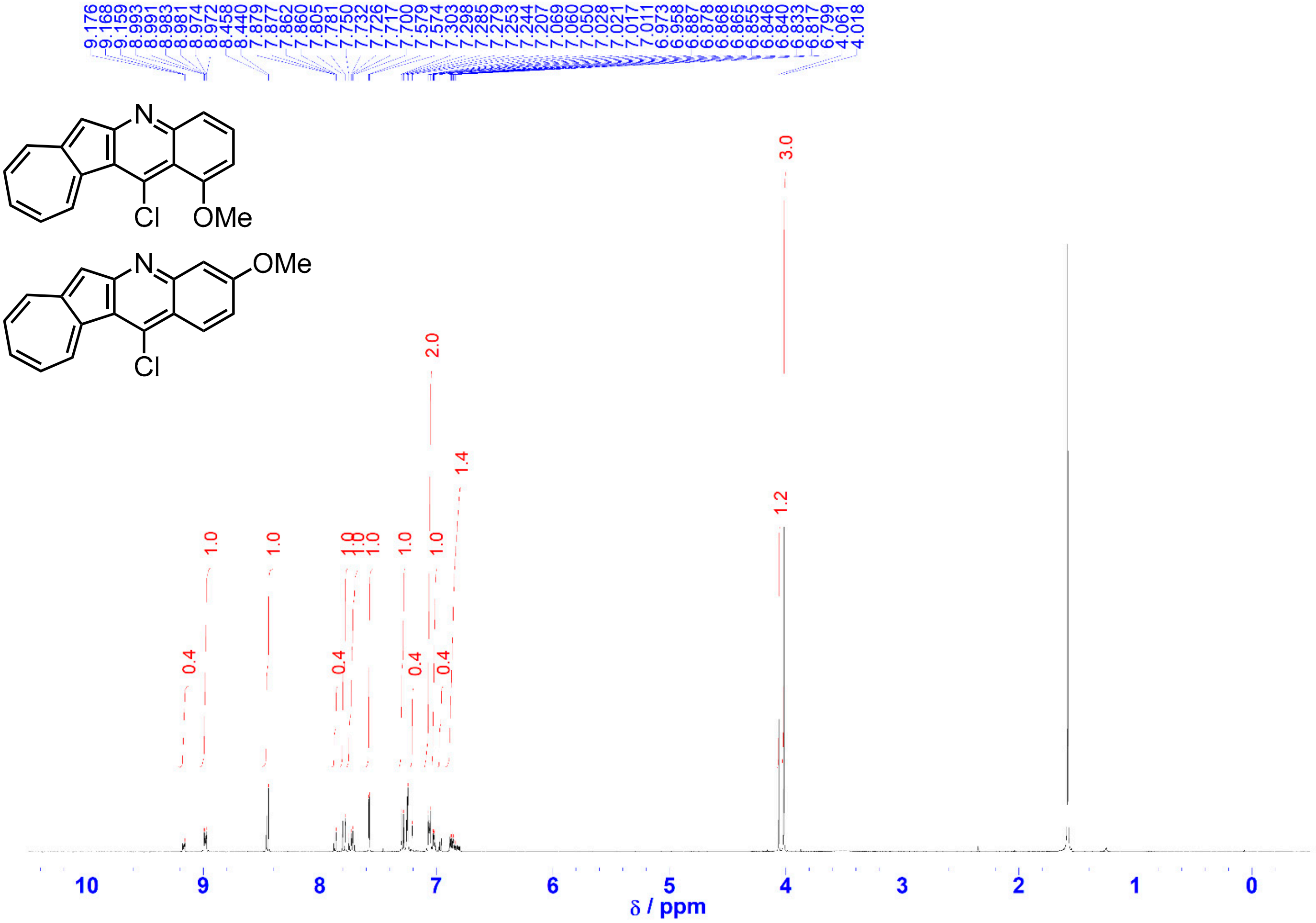

Figure S39. ^{1}H NMR spectrum of **4e** and **4e′** in $CDCl_3$ (500 MHz).

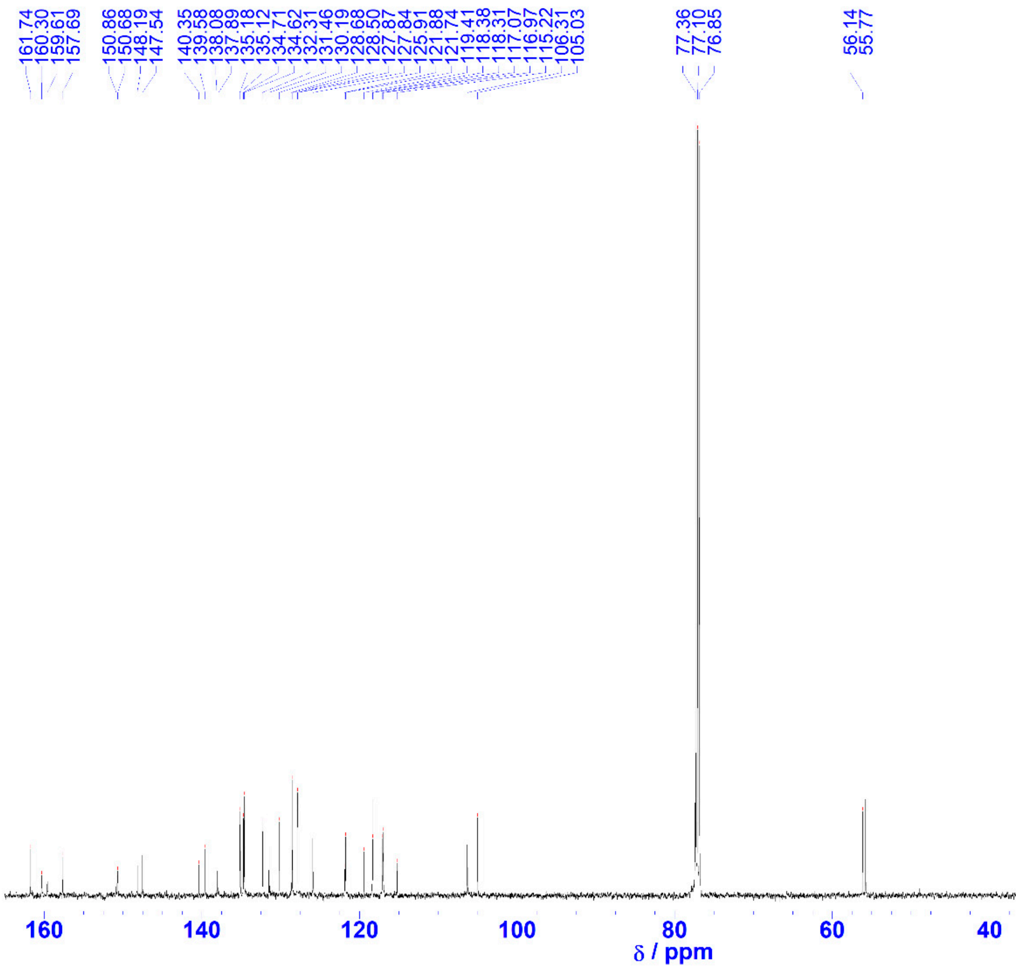

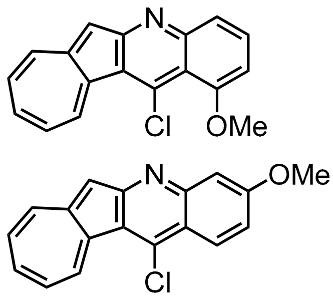

Figure S40. ^{13}C NMR spectrum of **4e** and **4e'** in $CDCl_3$ (125 MHz).

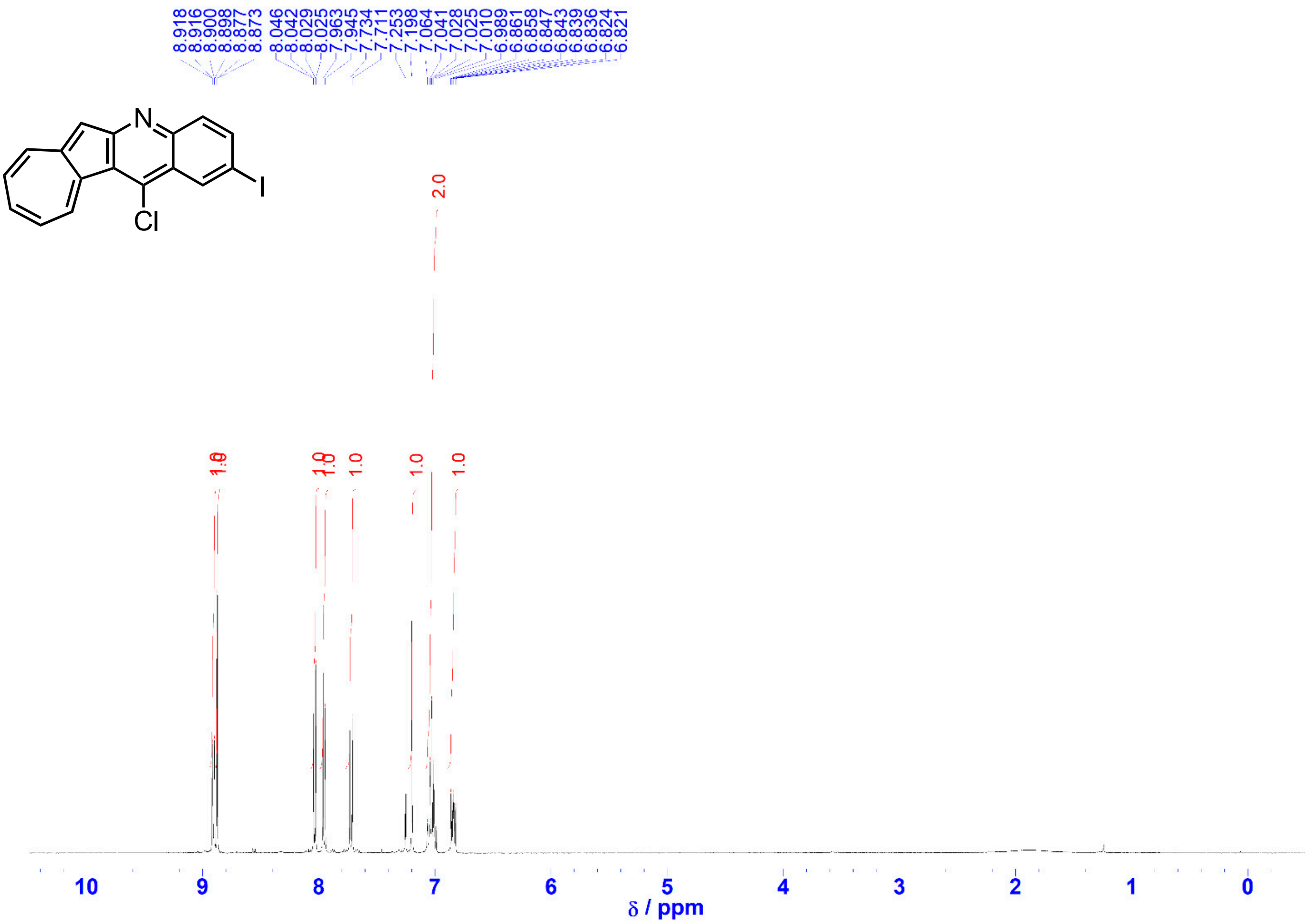

Figure S41. ^1H NMR spectrum of **4f** in $CDCl_3$ (500 MHz).

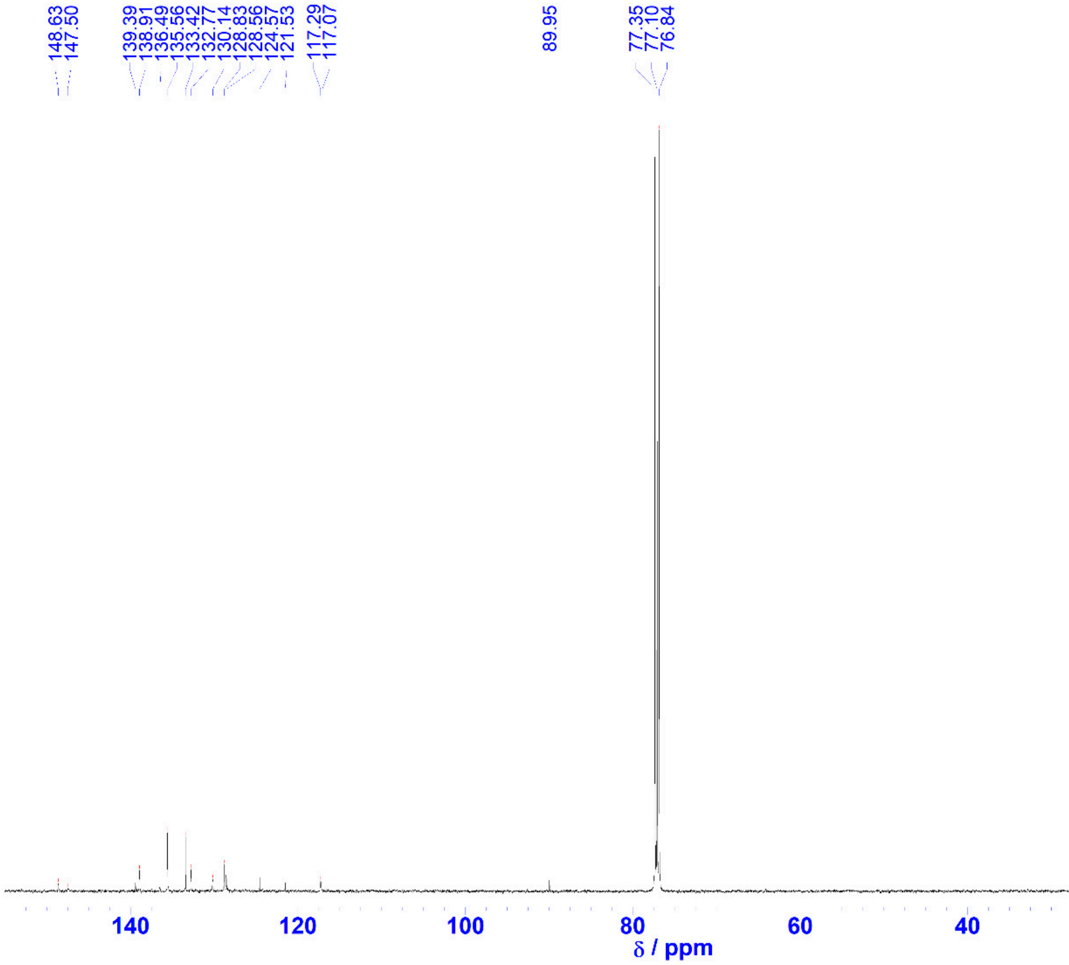

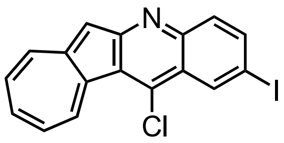

Figure S42. ^{13}C NMR spectrum of **4f** in $CDCl_3$ (125 MHz).

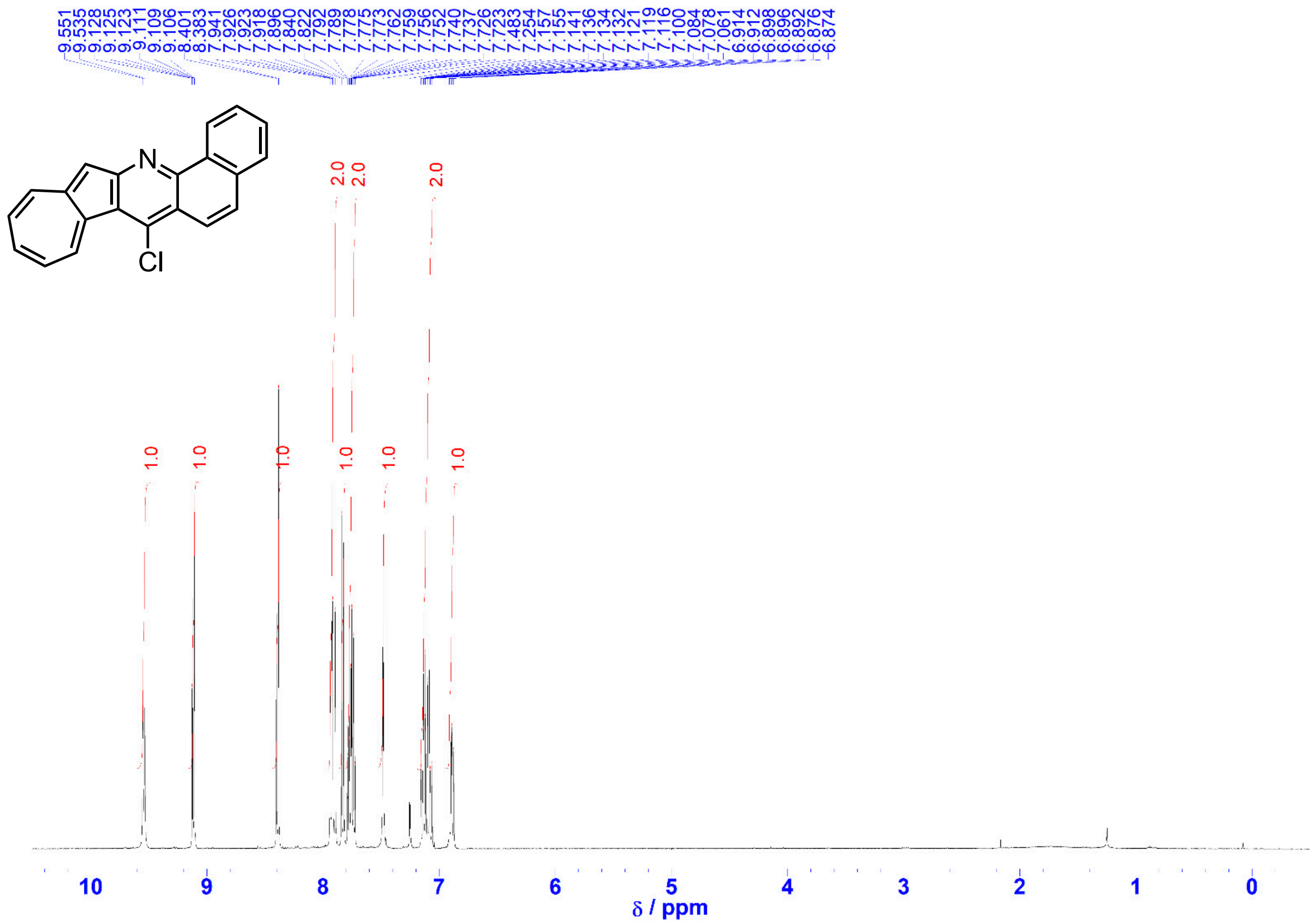

Figure S43. ^{1}H NMR spectrum of **4g** in $CDCl_3$ (500 MHz).

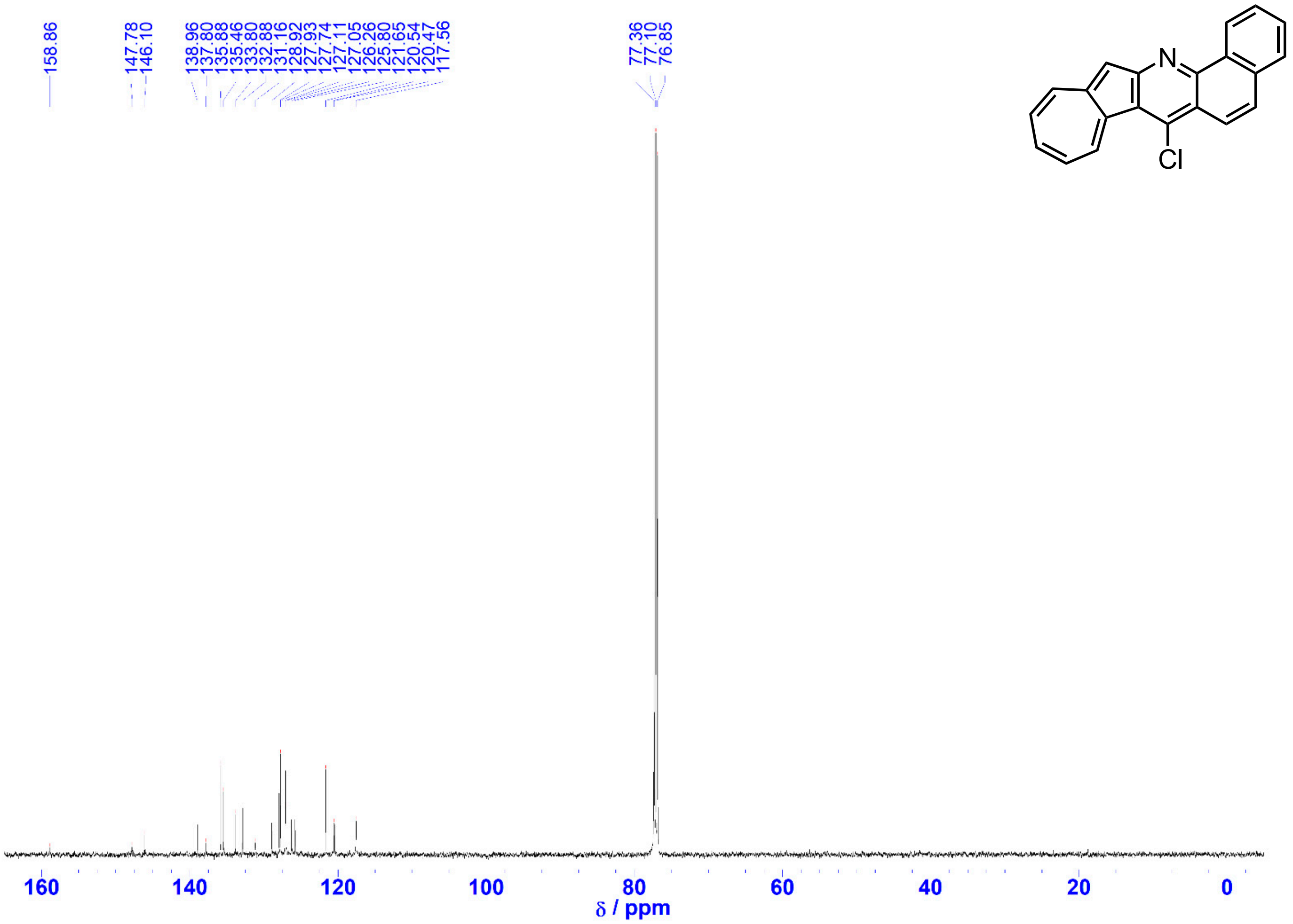

Figure S44. ^{13}C NMR spectrum of **4g** in $CDCl_3$ (125 MHz).

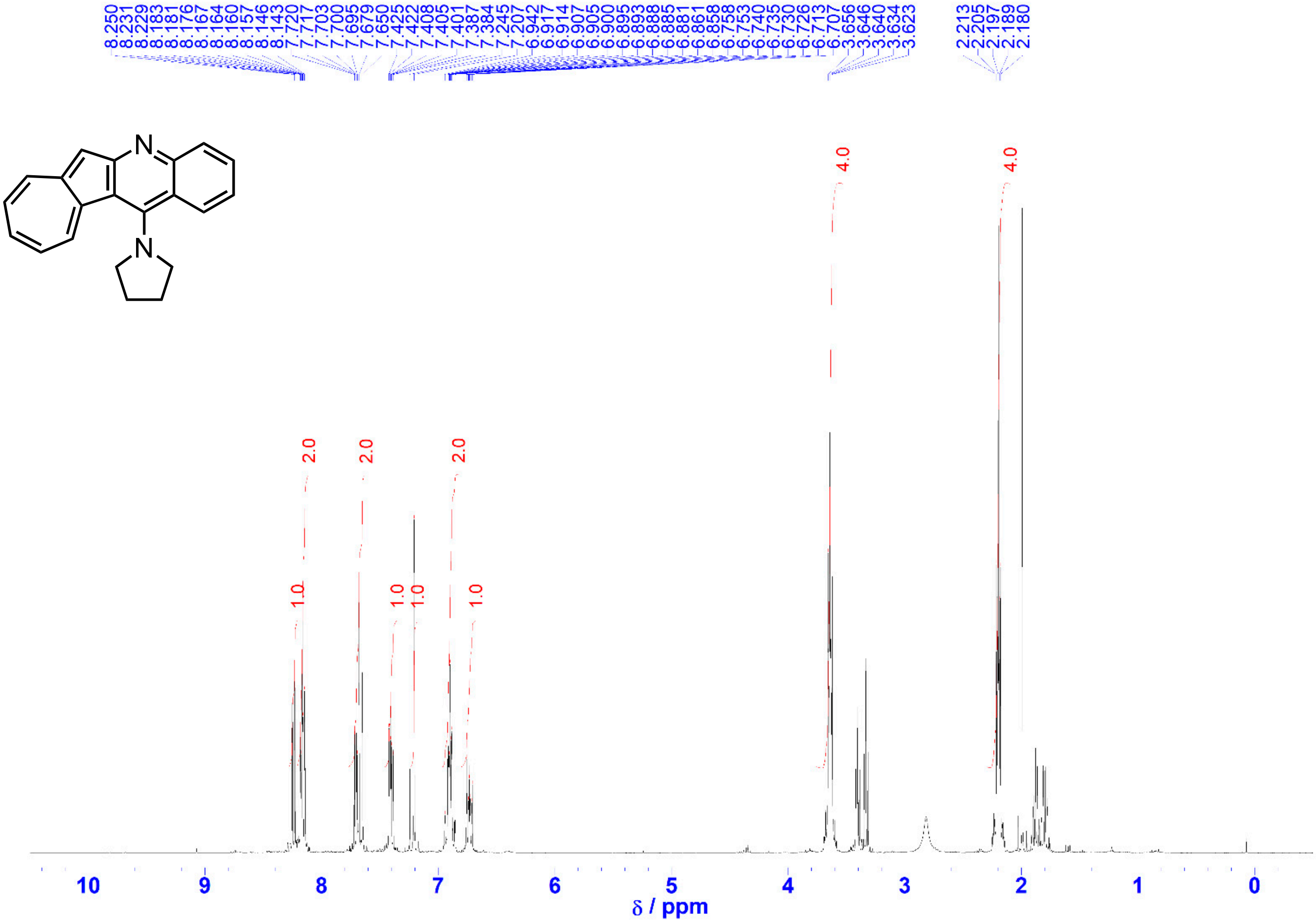

Figure S45. ^{1}H NMR spectrum of **5a** in $CDCl_3$ (500 MHz).

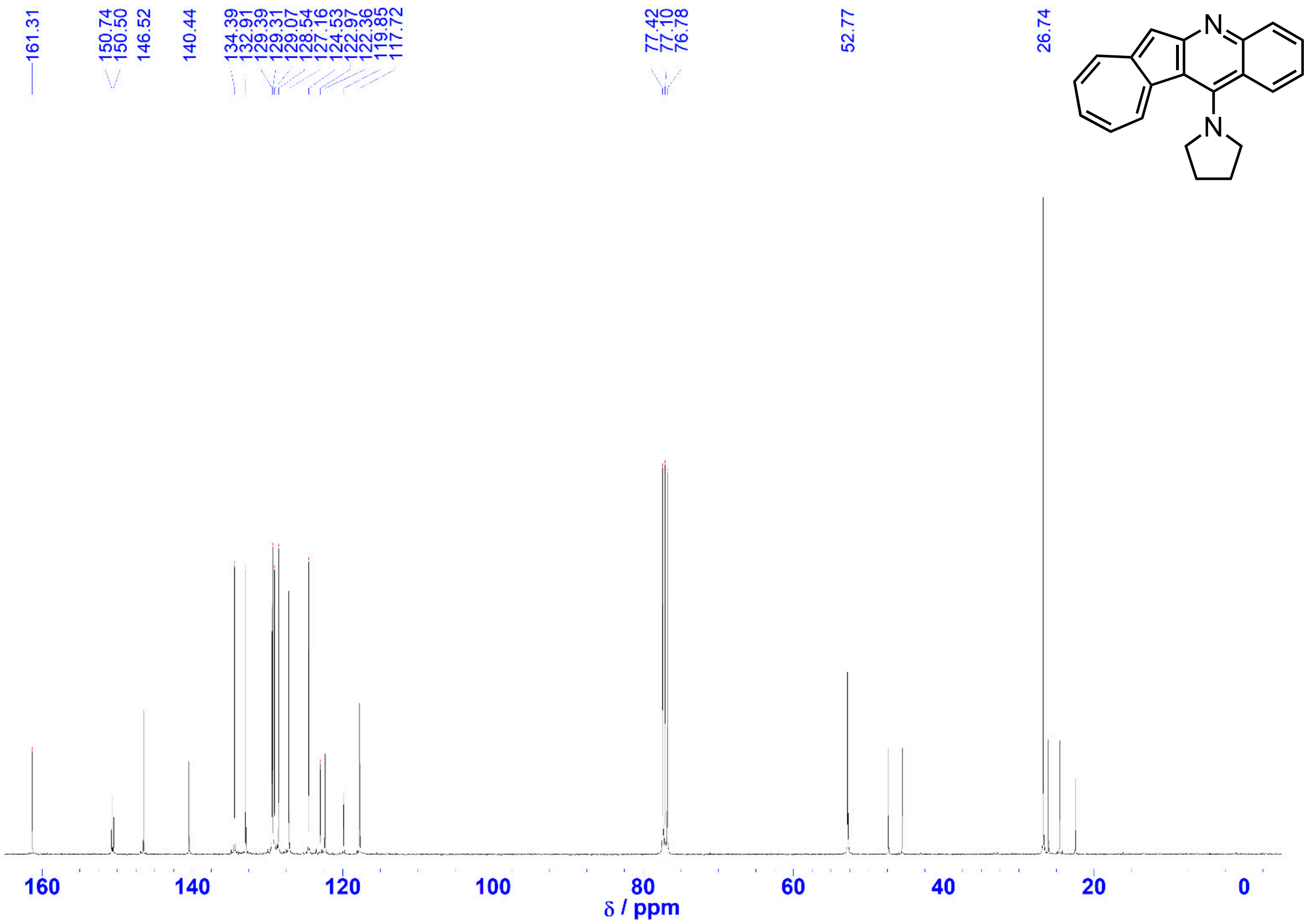

Figure S46. ^{13}C NMR spectrum of **5a** in $CDCl_3$ (125 MHz).

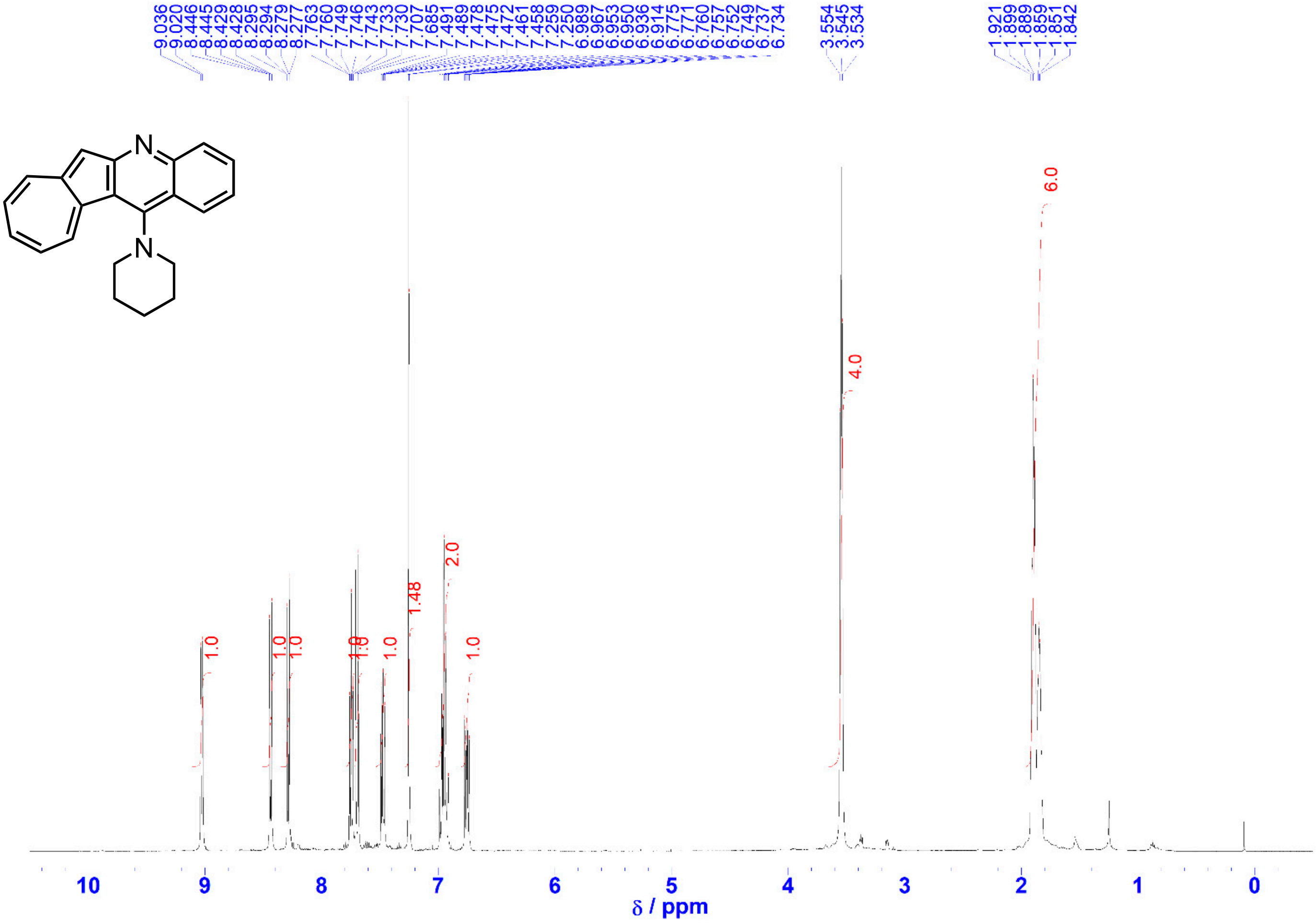

Figure S47. ^1H NMR spectrum of **5a** in $CDCl_3$ (500 MHz).

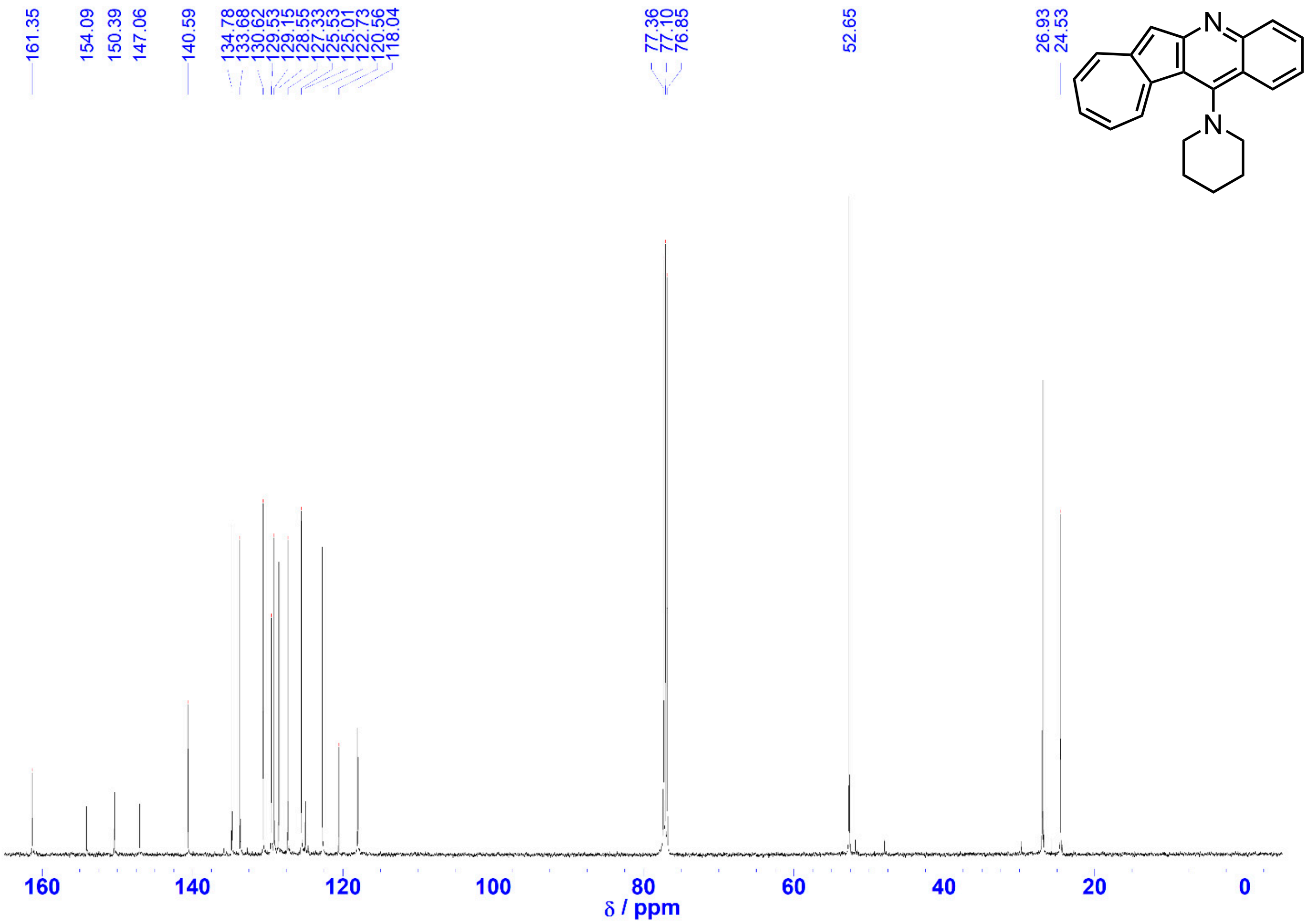

Figure S48. ^{13}C NMR spectrum of **5a** in $CDCl_3$ (125 MHz).

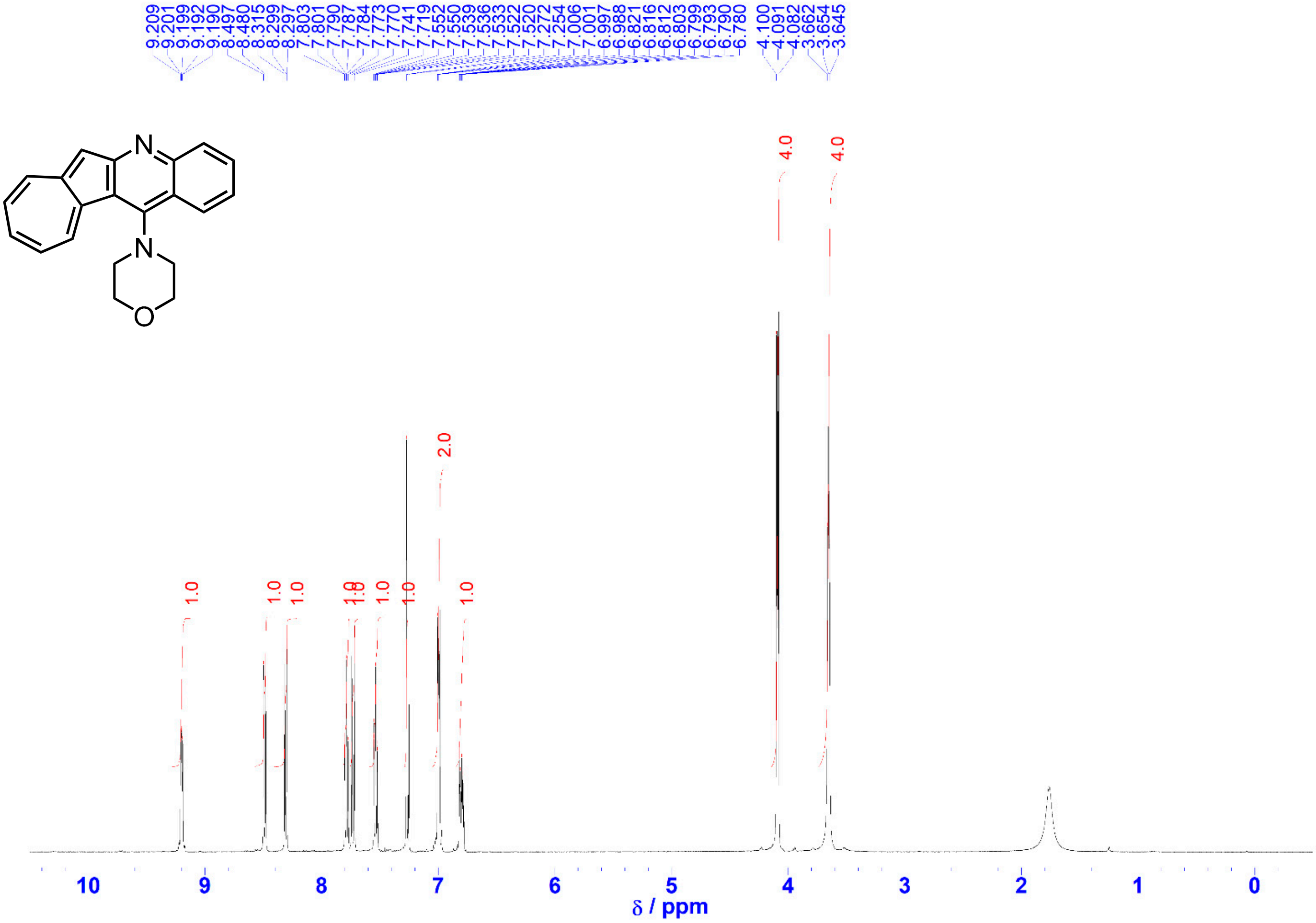

Figure S49. ^{1}H NMR spectrum of **5a** in $CDCl_3$ (500 MHz).

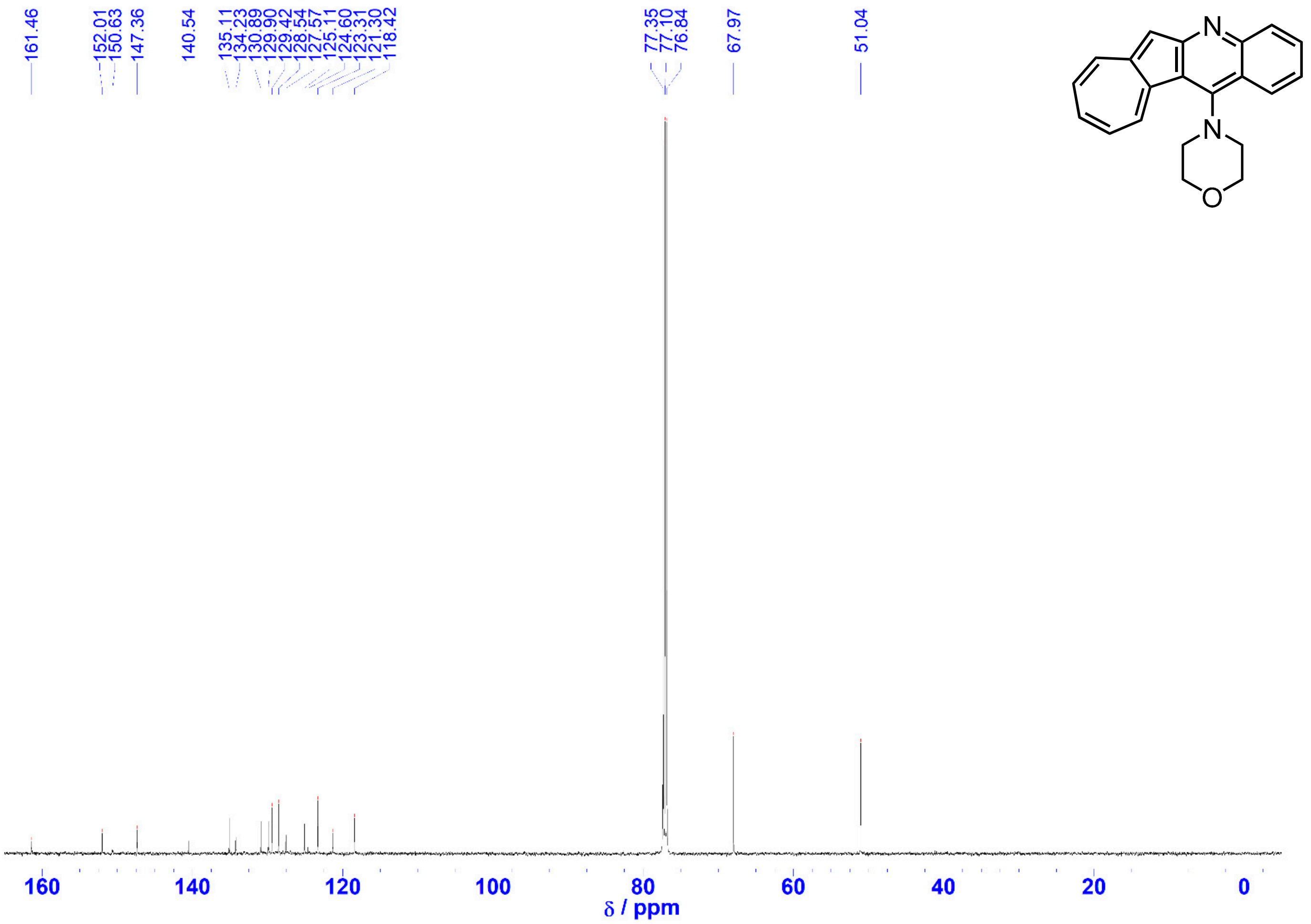

Figure S50. ^{13}C NMR spectrum of **5a** in $CDCl_3$ (125 MHz).
